# The increasing burden of diabetes and variations among the states of India: the Global Burden of Disease Study 1990–2016

**DOI:** 10.1016/S2214-109X(18)30387-5

**Published:** 2018-09-12

**Authors:** India State-Level Disease Burden Initiative Diabetes Collaborators, Nikhil Tandon, Ranjit M Anjana, Viswanathan Mohan, Tanvir Kaur, Ashkan Afshin, Kanyin Ong, Satinath Mukhopadhyay, Nihal Thomas, Eesh Bhatia, Anand Krishnan, Prashant Mathur, R S Dhaliwal, D K Shukla, Anil Bhansali, Dorairaj Prabhakaran, Paturi V Rao, Chittaranjan S Yajnik, G Anil Kumar, Chris M Varghese, Melissa Furtado, Sanjay K Agarwal, Megha Arora, Deeksha Bhardwaj, Joy K Chakma, Leslie Cornaby, Eliza Dutta, Scott Glenn, N Gopalakrishnan, Rajeev Gupta, Panniyammakal Jeemon, Sarah C Johnson, Tripti Khanna, Sanjay Kinra, Michael Kutz, Pallavi Muraleedharan, Nitish Naik, Chrisopher M Odell, Anu M Oommen, Jeyaraj D Pandian, Sreejith Parameswaran, Sanghamitra Pati, Narayan Prasad, D Sreebhushan Raju, Ambuj Roy, Meenakshi Sharma, Chander Shekhar, Sharvari R Shukla, Narinder P Singh, J S Thakur, Ranjit Unnikrishnan, Santosh Varughese, Denis Xavier, Geevar Zachariah, Stephen S Lim, Mohsen Naghavi, Rakhi Dandona, Theo Vos, Christopher J L Murray, K Srinath Reddy, Soumya Swaminathan, Lalit Dandona

## Abstract

**Background:**

The burden of diabetes is increasing rapidly in India but a systematic understanding of its distribution and time trends is not available for every state of India. We present a comprehensive analysis of the time trends and heterogeneity in the distribution of diabetes burden across all states of India between 1990 and 2016.

**Methods:**

We analysed the prevalence and disability-adjusted life-years (DALYs) of diabetes in the states of India from 1990 to 2016 using all available data sources that could be accessed as part of the Global Burden of Diseases, Injuries, and Risk Factors Study 2016, and assessed heterogeneity across the states. The states were placed in four groups based on epidemiological transition level (ETL), defined on the basis of the ratio of DALYs from communicable diseases to those from non-communicable diseases and injuries combined, with a low ratio denoting high ETL and vice versa. We assessed the contribution of risk factors to diabetes DALYs and the relation of overweight (body-mass index 25 kg/m^2^ or more) with diabetes prevalence. We calculated 95% uncertainty intervals (UIs) for the point estimates.

**Findings:**

The number of people with diabetes in India increased from 26·0 million (95% UI 23·4–28·6) in 1990 to 65·0 million (58·7–71·1) in 2016. The prevalence of diabetes in adults aged 20 years or older in India increased from 5·5% (4·9–6·1) in 1990 to 7·7% (6·9–8·4) in 2016. The prevalence in 2016 was highest in Tamil Nadu and Kerala (high ETL) and Delhi (higher-middle ETL), followed by Punjab and Goa (high ETL) and Karnataka (higher-middle ETL). The age-standardised DALY rate for diabetes increased in India by 39·6% (32·1–46·7) from 1990 to 2016, which was the highest increase among major non-communicable diseases. The age-standardised diabetes prevalence and DALYs increased in every state, with the percentage increase among the highest in several states in the low and lower-middle ETL state groups. The most important risk factor for diabetes in India was overweight to which 36·0% (22·6–49·2) of the diabetes DALYs in 2016 could be attributed. The prevalence of overweight in adults in India increased from 9·0% (8·7–9·3) in 1990 to 20·4% (19·9–20·8) in 2016; this prevalence increased in every state of the country. For every 100 overweight adults aged 20 years or older in India, there were 38 adults (34–42) with diabetes, compared with the global average of 19 adults (17–21) in 2016.

**Interpretation:**

The increase in health loss from diabetes since 1990 in India is the highest among major non-communicable diseases. With this increase observed in every state of the country, and the relative rate of increase highest in several less developed low ETL states, policy action that takes these state-level differences into account is needed urgently to control this potentially explosive public health situation.

**Funding:**

Bill & Melinda Gates Foundation; and Indian Council of Medical Research, Department of Health Research, Ministry of Health and Family Welfare, Government of India.

## Introduction

The burden of diabetes has steadily increased over the past quarter century in India and across the globe, with India contributing a major part of the global burden.^1^, ^2^, ^3^ Diabetes was identified as one of four priority non-communicable diseases (NCDs) targeted for action by the United Nations due to its growing disease burden.^2^, ^4^ In 2013, WHO developed targets for prevention and control of NCDs by 2025, which included a 25% reduction in mortality from NCDs, halting the rise in diabetes and obesity, and ensuring that at least 80% of patients have access to affordable basic technologies and essential medicines for NCDs.^5^, ^6^ The Sustainable Development Goals (SDGs) also include a target to reduce the proportion of premature deaths due to NCDs, including diabetes, by one third by 2030.^7^, ^8^ The National Health Policy 2017 of India aims to increase screening and treatment of 80% of people with diabetes and reduce premature deaths from diabetes by 25% by 2025.^9^

Overall diabetes burden estimates for the 1·3 billion population of India mask wide variations across the states of the country, many of which are comparable to large countries in terms of population. Attempts have been made previously to compile the trends of diabetes from studies done over several decades in different parts of India.^10^, ^11^, ^12^, ^13^, ^14^ However, no comprehensive analysis is available that reports diabetes estimates for every state of India over a long period of time. Health is a state subject in India, with the state budget contributing two-thirds towards overall government spending on health care and the central budget contributing the remainder.^15^, ^16^ It is therefore imperative to have robust and comprehensive estimates on the magnitude of diabetes and its risk factors in every state to enable planning for targeted policy and interventions. The India State-Level Disease Burden Initiative recently reported a varied epidemiological transition among the states of India between 1990 and 2016 as part of the Global Burden of Diseases, Injuries, and Risk Factors Study (GBD) 2016.^17^, ^18^ Here, we report the time trends and heterogeneity in diabetes burden across all states of India between 1990 and 2016.

**Research in context**

**Evidence before this study**

Existing evidence suggests that as part of the epidemiological transition from communicable diseases to non-communicable diseases, the burden of diabetes and its risk factors has been increasing in India. We searched PubMed and publicly available reports for estimates of diabetes burden across the states of India using the search terms “burden”, “cause of death”, “DALY”, “death”, “diabetes”, “epidemiology”, “India”, “morbidity”, “mortality”, “prevalence”, and “trends” on March 30, 2018, without language or publication date restrictions. We found many studies on the prevalence of diabetes in several urban settings and states, but no comprehensive report on the prevalence, deaths, and disability-adjusted life-years (DALYs) from diabetes in every state of India over a long duration of time.

**Added value of this study**

This study provides comprehensive estimates of the burden due to diabetes in every state of India from 1990 to 2016, based on all available data sources that could be accessed, using the standardised Global Burden of Diseases, Injuries, and Risk Factors Study methodology. It documents that, although substantial heterogeneity exists in the distribution of diabetes among the states of India, its prevalence has increased in every state between 1990 and 2016, with the age-standardised increase among the highest in the relatively less advanced states. Diabetes has had the highest increase in DALY rate among the major non-communicable diseases in India since 1990. We report that the prevalence of overweight, the most important risk factor for diabetes, has increased substantially in every state of India since 1990. The finding that in 2016 there were twice the number of people with diabetes in India for every 100 overweight adults versus the global average highlights the higher risk of diabetes in India.

**Implications of all the available evidence**

This comprehensive assessment of the burden of diabetes in every state of India over a quarter century is an acute reminder of the need to halt the potentially explosive increase of diabetes across the country. The state-specific trends in the prevalence of and DALYs from diabetes, and the prevalence of overweight, can serve as a useful guide for planning diabetes control in every state of India.

## Methods

### Overview

The India State-Level Disease Burden Initiative has recently reported the overall trends of diseases, injuries, and risk factors from 1990 to 2016 for every state of India.^17^, ^18^ That analysis was done as part of GBD 2016, which estimated disease burden due to 333 diseases and injuries and 84 risk factors using all available data sources that could be accessed. Disease grouping and risk grouping in GBD 2016 were organised into three broad categories and four levels, respectively. The India State-Level Disease Burden Initiative was supported by the efforts of several expert groups and a vast network of collaborators to identify and access all available data sources, assess their scope and quality for inclusion, and participate in the analysis and interpretation of the findings. The Health Ministry Screening Committee at the Indian Council of Medical Research and the ethics committee of the Public Health Foundation of India approved the work of this initiative. A detailed description of the metrics and analytical approaches used in GBD 2016 has been reported elsewhere.^19^, ^20^, ^21^, ^22^, ^23^ In this paper, we report findings on the prevalence of diabetes, its disease burden, and its risk factors across the states of India from 1990 to 2016. The GBD 2016 methods relevant for this paper are described in the appendix (pp 3–21), and a summary of the key points follows.

### Estimation of prevalence and years lived with disability

Diabetes was defined as fasting plasma glucose (FPG) greater than 126 mg/dL (7 mmol/L) or being on diabetes treatment.^21^, ^24^ The prevalence of diabetes was estimated by location, age, sex, and year using DisMod-MR, version 2.1, a disease modelling computational tool that is the standard GBD modelling approach for non-fatal health outcomes. The prevalence estimation process involved identification of all available data sources that could be accessed and their assessment for data extraction based on inclusion criteria; estimation of cause-specific prevalence using DisMod-MR modelling; ascertainment of severity distributions of sequelae; incorporation of disability weights to quantify severity; comorbidity adjustment of sequelae; and computation of years lived with disability (YLDs) from prevalence and disability weights for each location, age, sex, and year. GBD 2016 included the following sequelae as part of the direct diabetes burden estimation: vision loss due to diabetic retinopathy, diabetic retinopathy, diabetic neuropathy, diabetic neuropathy with foot ulcer, and diabetic neuropathy with treated or untreated amputation. The contribution of high FPG to ischaemic heart disease, stroke, chronic kidney disease, tuberculosis, and other conditions was estimated separately in GBD, and therefore we report the burden from these conditions separately.

The major data inputs for the distribution of diabetes in India included national health surveys, population-representative surveys and cohort studies, and a variety of published and unpublished studies (appendix pp 22–30).

### Estimation of deaths, years of life lost, and disability-adjusted life-years

Among the all-cause mortality rates, mortality due to diabetes was estimated using a variation of the GBD Cause of Death Ensemble modelling approach to estimate deaths separately in younger age groups (<25 years) and in older age groups (≥25 years).^19^, ^20^ All available data sources that could be accessed, including covariates, were used to develop a series of plausible models and, eventually, the best ensemble predictive model to produce estimates of deaths and years of life lost (YLLs) due to premature mortality by location, age, sex, and year. YLLs were calculated from age at death and GBD normative standard life expectancy at each age. Disability-adjusted life-years (DALYs), a summary measure of total health loss, were calculated for diabetes by summing YLLs and YLDs for each location, age, sex, and year. The sequelae of diabetes mentioned above that are included directly in the estimation of diabetes burden were accounted for in the estimation of diabetes DALYs. For other conditions attributable to high FPG, we report their DALYs separately.

The major data inputs for mortality estimation in India included Sample Registration System cause of death data and some other studies (appendix pp 22–30).

### Estimation of risk factor exposure and attributable disease burden

The GBD comparative risk assessment framework was used to estimate diabetes-related risk factor exposure and attributable disease burden.^23^ Exposure data for risk factors with a categorical or continuous distribution were collated from all available data sources that could be accessed, adjusted using age-sex splitting, and strengthened with the incorporation of covariates. The modelling approach integrated multiple data inputs and borrowed information across age, time, and location to produce the best possible estimates of risk exposure by location, age, sex, and year. For each risk factor, the theoretical minimum risk exposure level was established as the lowest level of risk exposure below which its relation with a disease outcome is not supported by the available evidence. Estimates of mean risk factor exposure, strengthened by covariates, were used to calculate summary exposure values for each risk—a metric ranging from 0% to 100%—to describe the risk-weighted exposure for a population or risk-weighted prevalence of exposure.

Estimates of mean FPG were produced by age, sex, year, and location using the spatial-temporal Gaussian process regression framework, adjusting mean FPG using a correction factor to account for people with diabetes in a population. For the purpose of attributing disease burden to FPG, the theoretical minimum risk exposure level for FPG was estimated to range from 81 to 97 mg/dL (mean 90) or 4·5 to 5·4 mmol/L (mean 5·0) as a risk for ischaemic heart disease, stroke, and chronic kidney disease, with a continuous risk–outcome relation above this FPG level.^23^ FPG more than 126 mg/dL (7 mmol/L) was considered to have a categorical risk–outcome relation with tuberculosis, neoplasms, Alzheimer's disease and other dementias, cataract, and others.^23^ Similarly, for the purpose of attributing disease burden to high body-mass index (BMI), the theoretical minimum risk exposure level for BMI at age 20 years or more was estimated to range from 20·0 to 25·0 kg/m^2^ (mean 22·5), and for age up to 19 years was based on the International Obesity Task Force BMI cutoff values for normal weight.^23^, ^25^ The definitions and method descriptions of other risk factors are described elsewhere.^23^

Estimation of attributable disease burden included ascertainment of relative risk of disease outcomes for risk exposure–disease outcome pairs with sufficient evidence of a causal relation in randomised controlled trials, prospective cohorts, or case-control studies as assessed using an approach similar to the World Cancer Research Fund grading system, and then estimation of population-attributable fractions for diseases caused by each risk factor.^23^ Estimates of deaths, YLLs, YLDs, and DALYs attributable to each risk factor for diabetes were produced by location, age, sex, and year.

The major data inputs for the risk factors in India included dietary and nutrition surveys from the National Nutrition Monitoring Bureau, national household surveys such as the National Family Health Survey, District Level Household Survey and Annual Health Survey, youth and adult tobacco surveys, household consumer expenditure surveys of the National Sample Survey Organisation, and several large population-level surveys (appendix pp 22–30).

GBD uses covariates, which are explanatory variables that have a known association with the outcome of interest, to arrive at the best possible estimate of that outcome when data on the outcome are scarce but data on the covariates are available.^19^, ^20^, ^21^, ^22^, ^23^ This approach was part of the estimation process for the findings presented here.

### Analysis presented in this paper

Findings are reported for 31 geographical units in India: 29 states, Union Territory of Delhi, and the union territories other than Delhi (combining the six smaller union territories of Andaman and Nicobar Islands, Chandigarh, Dadra and Nagar Haveli, Daman and Diu, Lakshadweep, and Puducherry). The states of Chhattisgarh, Uttarakhand, and Jharkhand were created from existing larger states in 2000, and the state of Telangana was created in 2014. For trends from 1990 onward, the data for these four new states were disaggregated from their parent states on the basis of data from the districts that now constitute these states. The findings are also presented for four groups of states based on epidemiological transition level (ETL) as described previously.^17^ Briefly, ETL state groups were defined on the basis of the ratio of DALYs from communicable, maternal, neonatal, and nutritional diseases to those from NCDs and injuries combined in 2016, with a lower ratio indicating higher ETL: low ETL (ratio 0·56–0·75), lower-middle ETL (0·41–0·55), higher-middle ETL (0·31–0·40), and high ETL (<0·31).^17^ We have previously reported that epidemiological transition ratios of the states of India have a significant inverse relation with the Socio-demographic Index calculated by GBD on the basis of income, education, and fertility levels, which indicates broad correspondence of the ETL groups with sociodemographic development levels.^17^

We present prevalence, deaths, and DALYs for diabetes and age-specific and sex-specific prevalence of diabetes for India in 1990 and 2016. We present the proportion of diabetes DALYs that could be attributed to various risk factors in 2016. We report the changes in overweight in adults aged 20 years or older using the definition of BMI 25 kg/m^2^ or more.^26^ We also assessed the contribution of high FPG to ischaemic heart disease, chronic kidney disease, cerebrovascular disease, tuberculosis, neoplasms, Alzheimer's disease and other dementias, cataracts, and others. Given the strong association between diabetes and overweight, we calculated the number of adults with diabetes per 100 overweight adults in India, and compared this estimate with the global average.^3^ We compared the age-standardised DALY rate of diabetes in 2016 in India with the global average.^3^

We present both crude and age-standardised estimates as relevant. Crude estimates provide the actual situation in each state that is useful for policy makers, and age-standardised estimates allow comparisons over time and between states after adjusting for the differences in the age structure of the population. The age-standardised rates were based on the GBD global reference population.^19^ The estimates are reported with 95% uncertainty intervals (UIs) where relevant. These intervals were based on 1000 runs of the models for each quantity of interest, with the mean considered as the point estimate and the 2·5th and 97·5th percentiles considered as the 95% UI (appendix p 21).^19^, ^20^, ^21^, ^22^, ^23^

### Role of the funding source

Some staff of the Indian Council of Medical Research are coauthors on this paper because they contributed to various aspects of the study and this analysis. The other funder of the study had no role in the study design, data collection, data analysis, data interpretation, or writing of this paper. The corresponding author had full access to all the data in the study, and had final responsibility for the decision to submit for publication.

## Results

**Figure 1:**
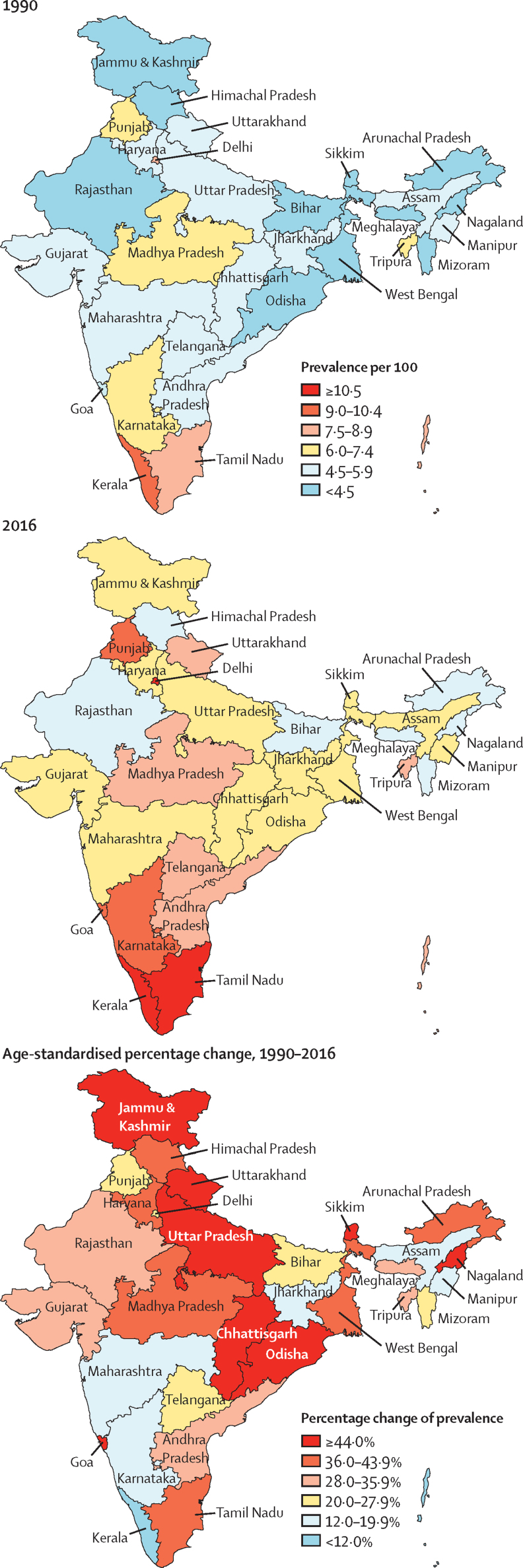
Crude prevalence of diabetes in adults aged 20 years or older in the states of India in 1990 and 2016 and change in age-standardised prevalence

**Figure 2:**
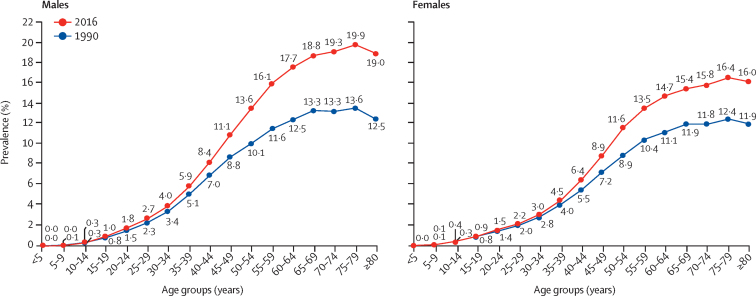
Age-sex-specific prevalence of diabetes in India, 1990 and 2016

There were 65·0 million (95% UI 58·7–71·1) prevalent cases of diabetes in India in 2016 (appendix p 31), compared with 26·0 million (23·4–28·6) in 1990.^27^ The crude prevalence of diabetes in adults aged 20 years or older in India increased by 39·4% from 5·5% (4·9–6·1) in 1990 to 7·7% (6·9–8·4) in 2016, with an increase in every state of India from 1990 to 2016. Age-standardised prevalence increased by 29·7% (26·5–32·6), with the highest increase in states with relatively low crude prevalence in 1990, most of which belonged to the low and lower-middle ETL groups (table 1; figure 1; appendix p 32). There was a 2·5 times variation in the prevalence of diabetes between the states in 2016, with the highest prevalence in the south Indian states of Tamil Nadu and Kerala (high ETL) and Delhi (higher-middle ETL), followed by Punjab and Goa (high ETL), and Karnataka (higher-middle ETL; figure 1; appendix p 32). Himachal Pradesh was a notable exception in the high ETL group, with a relatively low prevalence (appendix p 32). The age-specific prevalence of diabetes in India increased with increasing age in both 1990 and 2016. The divergence between the prevalence in 1990 and 2016 started in young adults, becoming statistically significant for men at 50–54 years (from 10·1% [95% UI 8·7–11·5] in 1990 to 13·6% [11·8–15·4] in 2016) and for women at 55–59 years (from 10·4% [9·1–11·7] in 1990 to 13·5% [11·9–15·2] in 2016) and remained significant in all older age groups (figure 2; appendix p 33).

**Table 1:** Prevalence of diabetes in adults aged 20 years or older by sex in the states of India grouped by ETL, 1990–2016

	**Crude prevalence per 100 (95% UI)**				**Age-standardised prevalence per 100 (95% UI)**			
	1990	2016	Absolute change, 1990–2016	Percentage change, 1990–2016	1990	2016	Absolute change, 1990–2016	Percentage change, 1990–2016
Both sexes*								
Low ETL (626 million)	4·7 (4·1–5·2)	6·6 (5·9–7·3)	1·9	42·5% (38·0–47·2)	5·7 (5·1–6·3)	7·7 (6·9–8·5)	2·0	35·8% (31·6–39·9)
Lower-middle ETL (92 million)	4·8 (4·3–5·4)	7·0 (6·3–7·6)	2·1	43·9% (37·6–50·1)	6·1 (5·4–6·8)	8·1 (7·4–8·9)	2·0	33·6% (27·9–40·0)
Higher-middle ETL (446 million)	5·5 (5·0–6·1)	7·5 (6·8–8·3)	2·0	36·4% (32·5–40·5)	6·8 (6·2–7·5)	8·6 (7·7–9·4)	1·7	25·4% (21·8–28·8)
High ETL (152 million)	8·3 (7·5–9·0)	11·8 (10·9–12·8)	3·6	43·6% (38·4–49·1)	9·9 (9·0–10·7)	12·5 (11·5–13·4)	2·6	26·9% (22·5–31·6)
India (1316 million)	5·5 (4·9–6·1)	7·7 (6·9–8·4)	2·2	39·4% (35·7–43·3)	6·7 (6·1–7·4)	8·7 (7·9–9·5)	2·0	29·7% (26·5–32·6)
**Men**								
Low ETL	5·0 (4·4–5·6)	7·3 (6·5–8·2)	2·3	46·3% (40·2–52·3)	6·1 (5·5–6·8)	8·6 (7·7–9·5)	2·5	40·4% (34·8–46·0)
Lower-middle ETL	5·0 (4·4–5·6)	7·4 (6·7–8·1)	2·4	47·0% (38·7–55·7)	6·5 (5·8–7·2)	8·8 (8·1–9·7)	2·4	37·1% (29·8–45·5)
Higher-middle ETL	6·1 (5·4–6·7)	8·4 (7·6–9·2)	2·3	37·7% (32·8–42·5)	7·6 (6·8–8·3)	9·7 (8·8–10·6)	2·1	27·9% (23·4–32·2)
High ETL	8·2 (7·4–9·0)	12·1 (11·2–13·0)	3·9	47·5% (40·9–54·7)	9·9 (9·0–10·8)	13·0 (12·0–13·9)	3·1	31·5% (25·5–37·4)
India	5·8 (5·2–6·5)	8·3 (7·5–9·1)	2·5	42·1% (37·9–46·8)	7·2 (6·5–7·9)	9·6 (8·8–10·5)	2·4	33·5% (29·5–37·5)
**Women**								
Low ETL	4·3 (3·8–4·7)	5·9 (5·3–6·5)	1·6	37·8% (32·8–42·5)	5·2 (4·7–5·8)	6·8 (6·1–7·6)	1·6	30·4% (26·0–34·6)
Lower-middle ETL	4·6 (4·1–5·2)	6·5 (5·8–7·2)	1·9	40·4% (33·3–47·1)	5·7 (5·1–6·4)	7·4 (6·7–8·2)	1·7	29·8% (23·0–36·2)
Higher-middle ETL	4·9 (4·4–5·4)	6·6 (5·9–7·4)	1·7	35·3% (30·6–40·0)	6·0 (5·4–6·7)	7·4 (6·7–8·2)	1·4	23·1% (19·1–29·8)
High ETL	8·3 (7·6–9·1)	11·6 (10·6–12·6)	3·3	39·7% (34·2–46·1)	9·8 (9·0–10·7)	12·1 (11·1–13·1)	2·2	22·6% (17·9–27·7)
India	5·1 (4·6–5·6)	7·0 (6·3–7·7)	1·9	36·2% (32·3–39·9)	6·3 (5·7–6·9)	7·9 (7·1–8·6)	1·6	25·6% (22·6–28·7)

ETL=epidemiological transition level. UI=uncertainty interval.

* Population in 2016 given in parentheses.

Diabetes contributed to 3·1% (95% UI 2·9–3·3) of the total deaths in India, with slight variation among men (2·9%, 95% UI 2·7–3·0) and women (3·4%, 3·0–3·7; appendix p 34). The crude death rate due to diabetes increased in India from 1990 to 2016 by 131% (111–150) from 10·0 (95% UI 8·9–11·0) to 23·1 (21·4–24·5) per 100 000, and the age-standardised death rate increased by 64% (48–79). Of the total deaths due to diabetes in 2016, 42·6% (41·6–43·9) were in people younger than 70 years, and this proportion was highest in the low ETL group (table 2). However, the crude death rate was highest in the high ETL group (table 2).

**Table 2:** Deaths from diabetes at age less than 70 years versus older age in the states of India grouped by ETL, 2016

	**Crude death rate per 100 000 (95% UI)**	**Deaths per 100 000 from diabetes at <70 years of age (95% UI)**	**Percentage of total deaths from diabetes at <70 years of age (95% UI)**	**Deaths per 100 000 from diabetes at ≥70 years of age (95% UI)**	**Percentage of total deaths from diabetes at ≥70 years of age (95% UI)**
Low ETL	18·3 (16·5–19·9)	8·8 (8·1–10·0)	46·6% (44·9–48·7)	318·2 (277·0–349·6)	53·4% (51·3–55·1)
Lower-middle ETL	21·2 (19·2–23·1)	9·1 (8·1–10·0)	41·3% (39·2–43·5)	374·3 (337·4–417·1)	58·7% (56·5–60·8)
Higher-middle ETL	23·4 (21·6–25·1)	10·0 (9·3–10·7)	41·0% (39·6–42·5)	371·7 (337·2–402·0)	59·0% (57·5–60·4)
High ETL	43·2 (38·7–47·2)	17·4 (15·8–19·2)	38·4% (36·6–40·4)	556·7 (495·4–614·3)	61·6% (59·6–63·4)
India	23·1 (21·4–24·5)	10·2 (9·6–10·7)	42·6% (41·6–43·9)	378·6 (344·5–404·3)	57·4% (56·1–58·4)

ETL=epidemiological transition level. UI=uncertainty interval.

**Figure 3:**
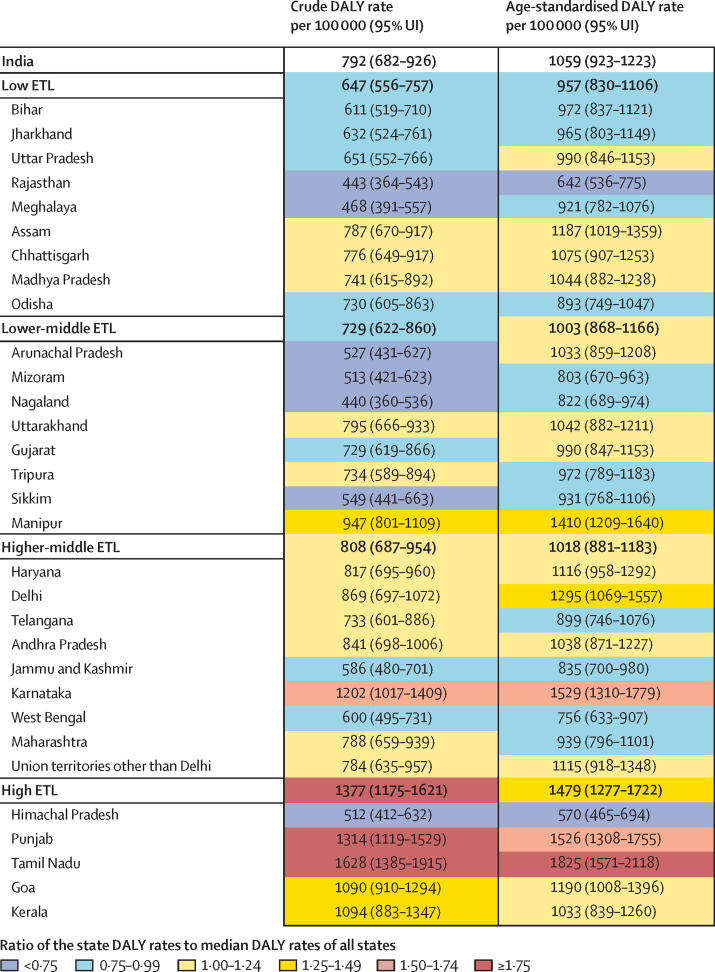
Crude and age-standardised DALY rates of diabetes in the states of India, 2016 DALY=disability-adjusted life-year. ETL=epidemiological transition level.

**Figure 4:**
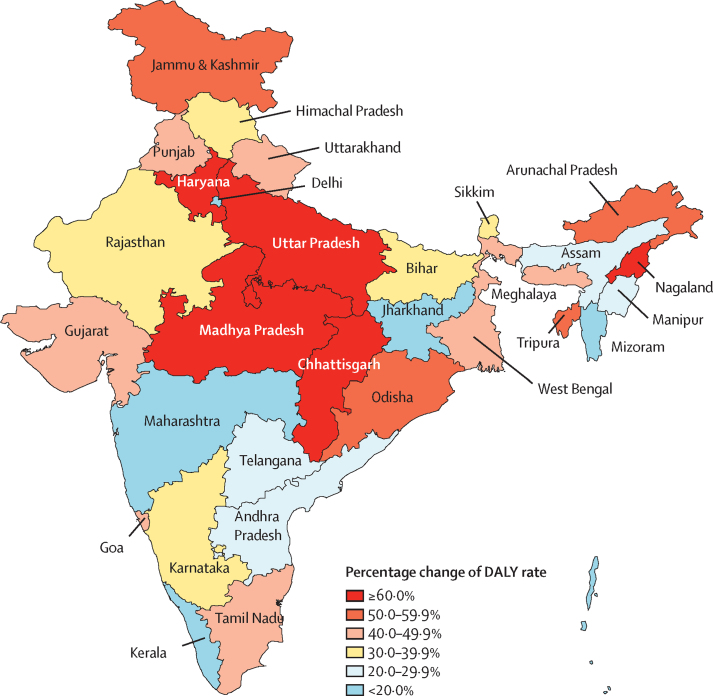
Percentage change in the age-standardised DALY rate of diabetes in the states of India, 1990–2016 DALY=disability-adjusted life-year.

Diabetes contributed to 2·2% (95% UI 2·1–2·4) of the total DALYs in India in 2016 (appendix p 34). Of the total diabetes DALYs in 2016, 57·2% were from YLLs and 42·8% from YLDs.^27^ The percentage of total DALYs due to diabetes was lowest in the low ETL group (1·7%, 95% UI 1·5–1·9) and highest in the high ETL group (4·3%, 4·0–4·7; appendix p 34). The crude DALY rate of diabetes in 2016 varied 3·7 times between the states, with the highest in Tamil Nadu and Punjab in the high ETL group, followed by Karnataka in the higher-middle ETL group; the age-standardised DALY rates were also highest in these three states (figure 3). The crude DALY rate of diabetes increased across all ETL state groups from 1990 to 2016. The increase in the age-standardised DALY rate since 1990 was generally the highest in states with a relatively low DALY rate in 1990 (figure 4; appendix p 35). The highest change exceeding 60% was in the states of Chhattisgarh, Uttar Pradesh, and Madhya Pradesh in the low ETL group; Nagaland in the lower-middle ETL group; and Haryana in the higher-middle ETL group. Among the major NCDs, diabetes had the highest increase in DALY rate in India from 1990 to 2016, with a crude increase of 80·0% (95% UI 71·6–88·5) and an age-standardised increase of 39·6% (32·1–46·7; appendix p 36).^17^ In 2016, the age-standardised DALY rate of diabetes in India was 1·3 times the global average.^3^

**Figure 5:**
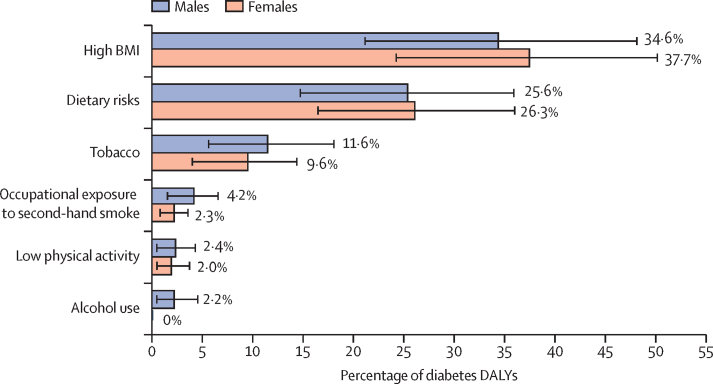
Percentage contribution of major risk factors to diabetes DALYs in India by sex, 2016 Error bars represent 95% uncertainty intervals. DALY=disability-adjusted life-year. BMI=body-mass index.

Among the risk factors contributing to diabetes in India in 2016, high BMI had the highest impact, with 36·0% (95% UI 22·6–49·2) of the diabetes DALYs attributed to it (figure 5). The other risk factors included dietary risks, tobacco use, occupational exposure to second-hand smoke, low physical activity, and alcohol use. It is important to note that the cumulative impact of the risk factors would be less than the sum of their individual contribution because the risk factors overlap. In addition, population-attributable fractions from components can add up to more than their sum even if they are independent. The contribution of high BMI was higher in females than in males, whereas the contribution of tobacco use, occupational exposure to second-hand smoke, and alcohol use were relatively higher in men than in women. The percentage of diabetes DALYs attributed to high BMI was relatively higher in the high ETL state group (50·4% [95% UI 35·3–63·1]; appendix p 37).

In addition to the 792 DALYs per 100 000 population from diabetes in India in 2016, high FPG also contributed substantially to DALYs for other conditions, which included ischaemic heart disease (607 DALYs [95% UI 384–918] per 100 000 population), chronic kidney disease (330 DALYs [285–383]), stroke (226 DALYs [149–324]), tuberculosis (103 DALYs [67–142]), cancers (30 DALYs [8–61]), Alzheimer's disease and other dementias (12 DALYs [3–26]), and cataract (11 DALYs [2–25]).

**Figure 6:**
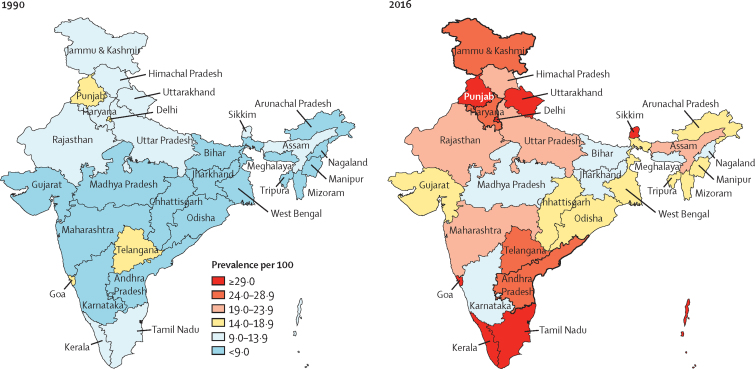
Prevalence of overweight in adults aged 20 years or older in the states of India, 1990 and 2016

The prevalence of overweight in adults aged 20 years or older in India increased from 9·0% (95% UI 8·7–9·3) in 1990 to 20·4% (19·9–20·8) in 2016. This increase occurred in every state of India, with a 3·5 times variation across the states in 2016 (figure 6; appendix pp 38–39). There were substantial variations in the magnitude of increase within each ETL state group: 58–147% in the low ETL group, 95–211% in the lower-middle ETL group, 36–311% in the higher-middle ETL group, and 101–184% in the high ETL group. The highest prevalence of overweight in 2016 was in the high ETL group (32·2%, 95% UI 31·1–33·4), and the lowest in the low ETL and lower-middle ETL groups (both 16·9%; appendix p 38). The states of Punjab, Goa, Kerala, and Tamil Nadu in the high ETL group; Uttarakhand and Sikkim in the lower-middle ETL group; and Delhi in the higher-middle ETL group had the highest prevalence of overweight in India.

Among every 100 overweight adults aged 20 years or older in India in 2016, there were 38 adults (95% UI 34–42) with diabetes, compared with the global average of 19 adults (17–21). This rate in India was higher for men (42 [95% UI 38–47] for every 100 overweight men) than for women (33 [30–37] for every 100 overweight women).

## Discussion

The findings in this report present a comprehensive picture of the rising trend of diabetes in every state of India over the past quarter century. The prevalence of diabetes in adults aged 20 years or older in India increased from 5·5% in 1990 to 7·7% in 2016. This prevalence was highest in the more developed states such as Tamil Nadu and Kerala and in Delhi. Previous prevalence studies on diabetes in India, although not strictly comparable with each other or with the present analysis on account of significant differences in sample selection and methodology, have also confirmed this increase in prevalence.^28^, ^29^, ^30^, ^31^ Studies have shown that Asian Indians have one of the highest incidence rates of diabetes among major ethnic groups, and that progression from prediabetes to diabetes appears to occur faster in this population.^32^ Our estimate of 65 million people with diabetes in India in 2016 is close to the 73 million estimate for 2017 reported by the International Diabetes Federation, with overlapping confidence intervals.^1^

The prevalence of diabetes increased in every state of India from 1990 to 2016, with a 2·5 times variation in prevalence across the states in 2016. Some of the highest increases in age-standardised prevalence from 1990 to 2016 were in the less developed states. This will pose novel challenges to the health-care systems in these states that are currently geared predominantly to the management of communicable diseases. However, the current relatively lower prevalence of diabetes in the less developed states offers a window of opportunity wherein measures can be planned and implemented in the less developed states to prevent further increases in the prevalence of diabetes and to strategically realign health-care services to meet the rising challenge of NCDs, especially diabetes. This is particularly important because many of the less developed states are among the most populous in India, and even small increases in diabetes prevalence in these states will have enormous implications for the magnitude of the diabetes burden in the country. However, it should also be noted that there is considerable heterogeneity in the trajectory of diabetes even within the ETL state groups. For instance, Himachal Pradesh, despite being a high ETL state, had a relatively low prevalence of diabetes, whereas Madhya Pradesh (low ETL state) and Tripura (lower-middle ETL state) had higher prevalence of diabetes than did other states in their ETL group. The magnitude of change in the prevalence of diabetes among the northeastern states of Manipur and Tripura was very different, even though both states belong to the lower-middle ETL group and are geographically adjacent to each other. This heterogeneity in the prevalence of diabetes within the ETL state groups highlights that individual NCDs do not necessarily follow the overall NCD trends that are used to define ETL. As compared with 1990, we observed higher age-specific prevalence of diabetes in 2016, starting in the young adult age groups, that reached statistical significance at age 50–54 years in men and 55–59 years in women. This finding is worrying because it portends further large increases in the burden of diabetes and its attendant complications in the productive age groups in the future.

Diabetes contributed to 3·1% of all deaths in India, with an increase in both crude (131%) and age-standardised (64%) death rates due to diabetes from 1990 to 2016. If this trend continues, it will pose challenges for meeting the national and global targets for reducing NCD deaths. The crude death rate was highest in the more developed high ETL state group. The increase in both the prevalence and death rates from diabetes points to possible shortfalls in the management of diabetes, such as delayed diagnosis, suboptimal glycaemic control, and failure to screen for early-stage complications, rendering people with diabetes more prone to the development of late-stage complications with attendant morbidity and mortality. A recent multistate study has reported that 47% of the diabetes cases in the population were undiagnosed, which highlights the poor awareness and detection of diabetes in India.^30^ It is important to note that 43% of deaths due to diabetes in India in 2016 were in people younger than 70 years. It is of concern that the states in the low ETL group, which had the lowest prevalence of diabetes, had the highest proportion of deaths due to diabetes among people younger than 70 years, again indicating possible inadequacies in diabetes management.

The increase in DALYs from diabetes since 1990 in India is the highest among major NCDs. The DALY rate of diabetes increased in every state of India from 1990 to 2016, with substantial heterogeneity of 3·7 times between the states in 2016. Diabetes did not feature among the top 30 causes of DALYs in India in 1990, but was the 13th leading cause of disease burden in 2016.^17^, ^18^, ^27^ Within India, the highest increases in diabetes DALYs occurred in states in the less developed low ETL group. This finding has important implications for the future economic progress of these states, many of which are among the least developed in India. It is also worrying that YLLs contributed more to the total diabetes DALYs than did YLDs; however, this is likely to change with improvements in sociodemographic parameters, as has occurred for other NCDs elsewhere in the world.^22^

Among risk factors contributing to DALYs across all causes in India, high FPG was the fifth leading risk factor.^17^, ^18^, ^27^ Besides contributing to diabetes, the contribution of high FPG to disease burden from other conditions is substantial in India, with DALYs attributable to high FPG for ischaemic heart disease, chronic kidney disease, stroke, tuberculosis, and other conditions together exceeding the DALYs attributable to high FPG for diabetes.

High BMI was the most important risk factor contributing to diabetes in India, suggesting that targeting overweight might offer the best results in slowing down the rising trend in diabetes across India. The prevalence of overweight in adults aged 20 years or older in India doubled from 1990 to 2016, with an increase observed in every state of India. This increase is attributable to the switch from traditional foodstuffs to energy-intense, nutrient-poor, high-carbohydrate diets; increasingly sedentary occupations; and low levels of recreational physical activity occurring as a consequence of urbanisation and socioeconomic transition.^27^, ^33^ The higher proportion of diabetes in India among overweight adults observed in this study compared with the global average highlights an increased risk of diabetes at lower levels of BMI among Indians. The possible explanations suggested for this phenomenon include low birthweight of Indians leading to low metabolic capacity coupled with high metabolic load that could be due to high levels of body fat, high dietary glycaemic load, and sedentary behaviour.^34^, ^35^ Overweight is related to dietary risks, which we also found to be a major risk factor for diabetes. In this GBD analysis, we found a relatively lower contribution of low physical activity and higher contribution of tobacco use to diabetes in India than in some previous reports.^35^, ^36^ These differences could be due to variations in exposure definitions of the risks and other aspects of study design. For example, the reason for the lower estimate of low physical activity could have been the inclusion of more cohort studies and quantification of total physical activity across all domains, whereas other studies might not have captured the full spectrum of physical activity, often limiting comparisons between leisure time or low physical activity with higher levels of physical activity.^37^ Similar reasons may be responsible for the differences in the association between tobacco use and diabetes in GBD versus the previous studies. These differences highlight the need for more research to understand the link between these risk factors and diabetes in the Indian context. In any case, physical activity has an important role in reducing overweight, and both physical activity and tobacco use control are important for preventing a variety of NCDs, and therefore should be promoted.

With the rising trajectory of diabetes in every part of India, more effective health policy interventions are needed to reverse this trend. The National Programme for Prevention and Control of Cancer, Diabetes, Cardiovascular Diseases and Stroke, launched in 2010, aims to prevent and control diabetes through behaviour and lifestyle changes, early diagnosis and management, and increasing health system capacity.^38^ Implementation of this programme across the states of India has been gradual, but is expected to help to reduce the burden of NCDs, including diabetes and its risk factors. Ayushman Bharat, the National Health Protection Mission, has been recently announced by the Indian Government, and includes health insurance for poor people and the establishment of 150 000 Health and Wellness Centres across India to provide compehensive primary health-care services that are aligned with the leading causes of disease burden, including diabetes.^39^, ^40^ Effective policy implementation, if combined with appropriate allocation of financial and human resources, and robust disease monitoring systems, would help in prevention, treatment, and reduction of diabetes deaths, which in turn would curb the growing disease burden in each state of India.

The general limitations of the GBD approach have been described elsewhere.^19^, ^20^, ^21^, ^22^, ^23^ Other limitations specific for India included an incomplete medically certified cause of death reporting system that covers only a small proportion of the population in India with variable coverage across the states. To address this limitation in the analysis, cause of death data obtained through verbal autopsy as part of the Sample Registration System from all states in India were used, which have been shown to be a reasonable alternative when cause of death data are not available from the vital registration system.^17^ Improvements are needed in the vital registration system of India for better data on cause of death. Additionally, data on sequelae of diabetes are not available from all parts of India, and although population-based FPG data have become available recently from nationwide surveys, these are not available for earlier years. Another challenge is the potential variability in the quality of different large-scale surveys that might lead to discrepant estimates from different sources. However, the use of multiple sources in the GBD approach minimises the possibility of erroneous trends. The strength of the findings in this report includes calculation of diabetes estimates and the major risk factors using all available data sources that could be accessed for each state of India, application of standard GBD methodology, and analysis and interpretation of findings over the past 26 years with inputs from leading health scientists and policy makers in India.

In conclusion, although the prevalence of diabetes remains higher in economically and epidemiologically advanced states, it has increased more rapidly in the less developed states, which are home to a large proportion of India's population. The increase in prevalence of and premature deaths due to diabetes highlighted here, along with other state-specific findings, underlines the need for policy and health-system action commensurate with disease burden in each state to ensure more effective prevention and management of diabetes. If uncontrolled, the health costs of diabetes and its complications are likely to take a heavy toll on India's health-care system in the coming decades. India should not lose the opportunity to address the major risks for diabetes before the situation gets further out of control.
