# Supplementary material for: The increasing burden of diabetes and variations among the states of India: the Global Burden of Disease Study 1990–2016
Source: Lancet Glob Health. 2018 Sep 12;6(12):e1352–62. doi: 10.1016/S2214-109X(18)30387-5 (PMC6227383; doi:10.1016/S2214-109X(18)30387-5)
Supplement: Supplementary appendix [file mmc1.pdf]

THE LANCET
Global Health

Supplementary appendix

This appendix formed part of the original submission and has been peer reviewed. We post it as supplied by the authors.

Supplement to: India State-Level Disease Burden Initiative Diabetes Collaborators. The increasing burden of diabetes and variations among the states of India: the Global Burden of Disease Study 1990–2016. *Lancet Glob Health* 2018; published online Aug 12. http://dx.doi.org/10.1016/S2214-109X(18)30387-5.

The increasing burden of diabetes and variations among the states of India: the Global Burden of Disease Study 1990-2016

India State-Level Disease Burden Initiative Diabetes Collaborators

Web Appendix

Correspondence to: Prof. Lalit Dandona, lalit.dandona@phfi.org

Table of Contents

1. GBD 2016 diabetes burden estimation methods 3
2. GBD 2016 India data inputs for diabetes mortality, morbidity, risk factors, and covariates 22
3. Number of persons with diabetes in the states of India, 2016 31
4. Prevalence of diabetes in adults 20 years of age or more in the states of India, 1990 and 2016 32
5. Age-sex-specific prevalence of diabetes in India, 1990 and 2016 33
6. Percent of total deaths and DALYs due to diabetes in the states of India grouped by epidemiological transition level, 2016 34
7. Change in DALY rate of diabetes in the states of India, 1990 to 2016 35
8. Change in DALY number and percent change in rates for the leading 30 causes in India, 1990 to 2016 36
9. Percent contribution of major risk factors to diabetes DALYs in the states of India grouped by epidemiological transition level, 2016 37
10. Prevalence of overweight in adults 20 years of age or more in the states of India, 1990 and 2016 38

1. GBD 2016 diabetes burden estimation methods

The material presented here is adapted from the following sources:

- GBD 2016 Disease and Injury Incidence and Prevalence Collaborators. Global, regional, and national incidence, prevalence, and years lived with disability for 328 diseases and injuries for 195 countries, 1990–2016: a systematic analysis for the Global Burden of Disease Study 2016. Lancet 2017; 390: 1211–59.
- GBD 2016 Causes of Death Collaborators. Global, regional, and national age-sex specific mortality for 264 causes of death, 1980–2016: a systematic analysis for the Global Burden of Disease Study 2016. Lancet 2017; 390: 1151–210.
- GBD 2016 Risk Factors Collaborators. Global, regional, and national comparative risk assessment of 84 behavioural, environmental and occupational, and metabolic risks or clusters of risks, 1990–2016: a systematic analysis for the Global Burden of Disease Study 2016. Lancet 2017; 390: 1345–422.

The GBD cause list is organised hierarchically into four levels. At each level of the hierarchy, the set of causes is mutually exclusive and collectively exhaustive. Diabetes is a level 3 cause, which falls under level 2 cause of "diabetes, urogenital, blood, and endocrine diseases" belonging to level 1 cause of "non-communicable diseases".

A. GBD case definitions of diabetes mellitus and inclusions

Diabetes mellitus parent
Diabetes mellitus (DM) was defined as fasting plasma glucose (FPG) > 126 mg/dL (7 mmol/L) or being on treatment for diabetes.

Uncomplicated diabetes mellitus
Cases of DM that do not have any of the following complications: neuropathy, foot ulcer, leg amputation, or vision loss.

Diabetic neuropathy
Cases of DM that experience diagnosable neuropathy.

Diabetic foot due to neuropathy
Cases of DM that currently have a foot ulcer.

Diabetic neuropathy and amputation with treatment
Cases of DM that have had a leg amputation above or below the knee, with treatment consisting of a prosthetic limb.

Diabetic neuropathy and amputation without treatment
Cases of DM that have had a leg amputation above or below the knee, with no prosthetic limb.

Moderate vision impairment due to diabetes mellitus
Cases of DM that have moderate vision loss due to diabetic retinopathy.

Severe vision impairment due to diabetes mellitus
Cases of DM that have severe vision loss due to diabetic retinopathy.

Blindness due to diabetes mellitus
Cases of DM that have blindness due to diabetic retinopathy.

The above are included in the direct burden estimation from diabetes. In addition, the following burden related to high FPG is assessed separately in GBD:

1. Chronic kidney disease, ischaemic heart disease, stroke, and peripheral vascular disease due to high FPG as a continuous variable.
2. Tuberculosis, liver cancer, pancreatic cancer, ovarian cancer, colorectal cancer, bladder cancer, lung cancer, breast cancer, glaucoma, cataract, and Alzheimer's disease and other dementias due to high FPG as a categorical variable.

B. List of ICD codes mapped to the GBD cause list

The codes used by GBD Study 2016 from the 9th and 10th revisions of the International Statistical Classification of Diseases and Related Health Problems (ICD) are listed below:

**Cause**	**ICD10**	**ICD9**
Diabetes mellitus due to underlying condition	E08.0-E08.9	
Drug or chemical induce diabetes mellitus	E09.0-E09.9	
Type 1 diabetes mellitus	E10.1-E10.9	
Type 2 diabetes mellitus	E11.0-E11.9	
Other specified diabetes mellitus	E13.0-E13.9	
Unspecified diabetes mellitus	E14.0-E14.9	
Syndrome of infant of mother with gestational diabetes	P70.0	775.0
Syndrome of infant of a diabetic mother	P70.1	
Neonatal diabetes mellitus	P70.2	775.1
Secondary diabetes mellitus		249.0-249.9
Diabetes mellitus		250.0– 250.9
Polyneuropathy in diabetes		357.2

C. GBD data and analysis framework

The overview of data inputs and analysis framework for GBD is shown in the following flowchart:

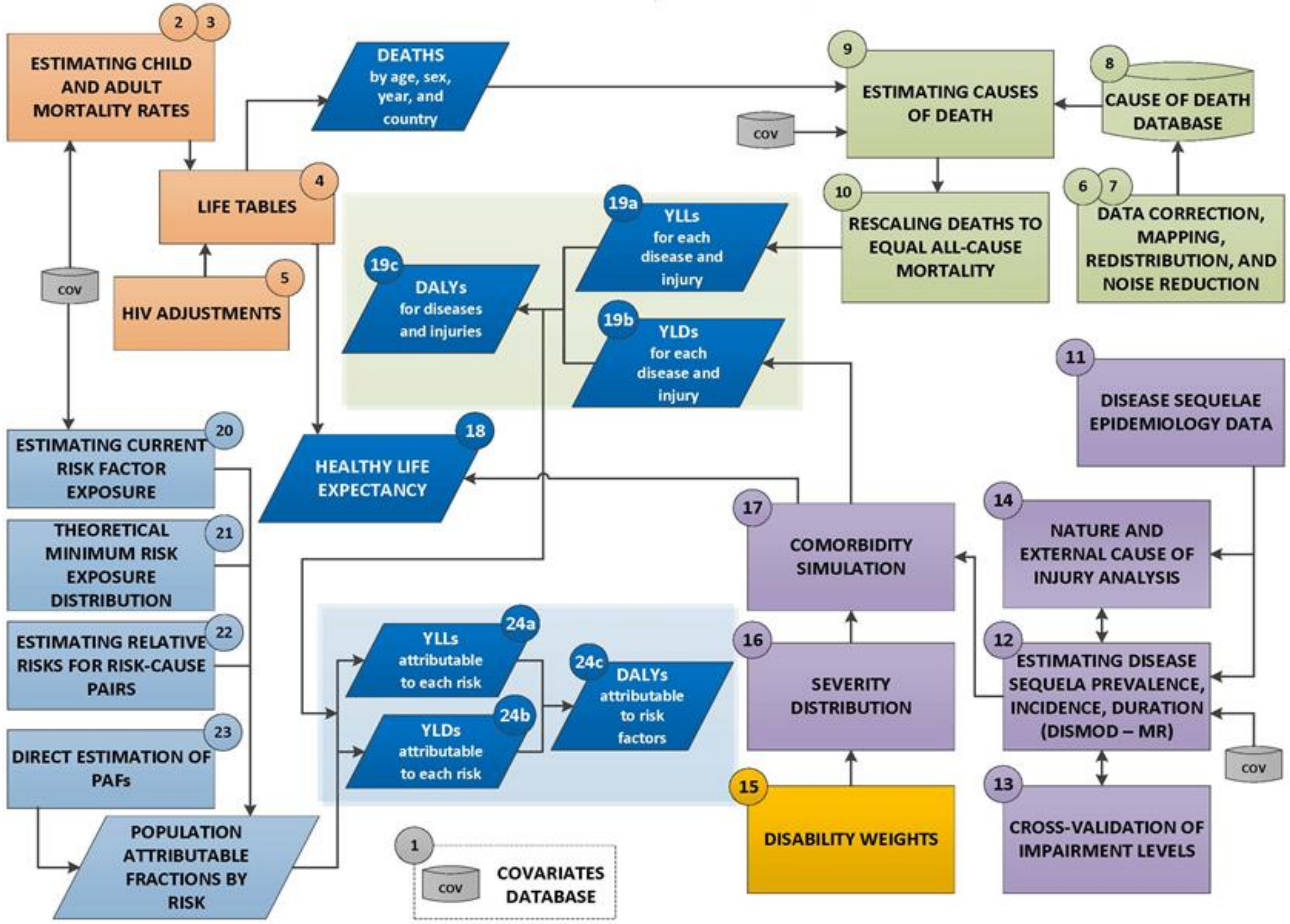

YLLs is years of life lost. YLDs is years lived with disability. DALYs is disability- adjusted life- years. PAFs is population attributable fractions. Rectangular boxes represent analytical steps, cylinders represent databases, and parallelograms represent intermediate and final results.

The flowchart above illustrates the flow of the key components of the GBD estimation process, including:

1. Incorporation of appropriate covariates (step 1)
2. All- cause mortality estimation (steps 2-5): the data come from sources such as censuses, surveys and vital registrations. The all-cause mortality estimation process (steps 2-4) can be divided into four distinct but interconnected areas: child mortality and adult mortality between ages 15 and 60, estimation of a complete set of age-specific death rates, estimation of HIV mortality and final estimates of age-specific mortality including HIV and fatal discontinuities (also known as mortality shocks) (step 5).
3. Causes of death estimation (steps 6-9): cause of death data are derived from vital registrations, verbal autopsy studies, mortality surveillance and, for selected causes, police records, crime reports and data collection systems for deaths due to conflict and natural disasters (step 7). Extensive data corrections and redistributions of ill-defined causes are made to correct for measurement bias between data sources. Cause of death ensemble modelling (CODEm), an ensemble model, is a systematized approach to analysing cause of death data for all but a few causes (step 9). CODEm explores a wide range of modelling approaches and varying predictive covariates to find an ensemble of best-performing models based on statistical tests. To do so, 30% of the data are withheld from each model and the model fit is evaluated by how well it covers the data that were left out. By repeating this process many times over the best performing models are selected. As all results in GBD are estimated 1,000 times over to propagate all sources of uncertainty, we end up with an ensemble of up to 100 or more different types of models and covariates that are selected among the 1,000 runs.
4. Rescaling deaths to equal all-cause mortality (step 10): as all these estimates are made separately for each disease and injury, the sum of these could exceed or fall below the all-cause mortality estimated from the demographic analyses of steps 2 to 5. Therefore, we rescale all deaths by age, sex, geography, year and cause to match the all-cause death estimates (this process is called CoDCorrect).
5. Estimation of disease sequelae prevalence, incidence, and duration (steps 11-12): population surveys, cohort studies, administrative records of hospitalisations and other health service encounters, disease registries, notifications, surveillance systems are the main data sources for non-fatal estimation (step 11). Extensive corrections of data to deal with measurement bias arising from study design or case definitions are applied. DisMod-MR 2.1 is the main analytical tool for non-fatal estimation (step 12). It is a Bayesian meta-regression software program that uses a lognormal model. The meta-regression component allows corrections for known sources of measurement error. Its core function is to make estimates of prevalence and incidence of disease that are consistent with data on mortality risk and remission (defined in GBD as the ‘cure rate’). For a select number of causes that do not fit well in the three state model (alive without disease, prevalent case of disease and death) of DisMod-MR 2.1 we use alternative modelling strategies.
6. Cross- validation of impairment levels (step 13): for a number of impairments in GBD terminology, such as anaemia, heart failure, hearing and vision loss, we first estimate the total levels of prevalence and incidence and then ensure that all sequelae of diseases that lead to this impairment add up to the total.
7. Analysis of the nature and external cause of injury is done separately (step 14).
8. Assignment of severity distributions for the main disabling conditions (step 15): in GBD terminology sequelae are the disabling consequences for which we make estimates. All sequelae are defined to be mutually exclusive and collectively exhaustive. Many diseases have sequelae with a gradation by severity such as mild, moderate and severe dementia. Often the epidemiological data on severity distribution is sparse. Therefore, we first model the epidemiology of all cases of disease and then apply a severity distribution from the sparser data.
9. Assignment of disability weights for health states (step 16): each sequela is matched with a health state or combination of health states for which we have a disability weight which quantifies the relative severity. Disability weights were derived from population and internet surveys of over 60,000 respondents answering pair-wise comparison question of random combinations of health states. Each pair of health states was described with brief lay descriptions highlighting the main symptoms and impairments. Respondents were asked to nominate the ‘healthier’ of each presented pair. Analytical methods exist to formalise the intuition that if the majority of respondents nominate one health state in a pair as the healthier these lie farther apart on a severity scale than pairs assigned similar proportions as the healthier. In order to anchor estimates on a 0-1 scale of severity, a subset of respondents was asked additional population health equivalence questions on a selection of health states. These questions ask for a choice of the greater amount of health produce by two health programs; one that prevented sudden death in 1,000 persons and another that prevented the onset of a GBD health state for the rest of 2,000, 5,000 or 10,000 persons’ lives.
10. Simulation of comorbidity (step 17): the last step of non-fatal estimation is a microsimulation (‘COMO”) to deal with comorbidity. For every age, sex, geography and year, 40,000 hypothetical persons are generated who have none, one or more of the GBD sequelae. In those with multiple sequelae their

combined level of disability is estimated multiplicatively. That means we assume the disability from having two health states is less than the sum of the corresponding disability weights. This avoids assigning disability greater than one to any individual which would indicate that person is worse off than being dead.

11. Estimation of healthy life expectancy (step 18): health life expectancy is estimated from the life tables generated in step 4 and the all-cause YLD rates from step 19b.
12. Computation of YLLs, YLDs, and DALYs from diseases and injuries with uncertainty (steps 19a-19c): YLLs (step 19a) are estimated as the product of counts of death by ages, sex, geography, year and cause and a normative life expectancy at the age of the death. The GBD standard life expectancy used as this norm is a compilation of the lowest observed mortality rates by age in all mortality data collections of populations greater than 5 million. The standard life table reflects a life expectancy at birth of 86.59 years. YLDs are the output from COMO (step 19b). DALYs are the simple addition of YLLs and YLDs (step 19c).
13. Risk factor estimation (steps 20-24): GBD 2016 also makes estimates for individual and combined risk factors. This involves estimation of risk factor exposure (step 20); the formulation of a minimum level of exposure to each risk that is associated with the least amount of health loss (step 21); derivation of relative risks of disease outcomes for each pair of a risk factor and a disease or injury for which there is judged to be sufficient evidence of a causal relationship (step 22); and the estimation of population attributable fractions of disease caused by each risk factor. For a few risk-outcome pairs it is hard to define exposure and a corresponding risk while directly observed proportions of disease are available, such as for the proportion of HIV/AIDS due to unsafe sex or injecting drug use (step 23). For combinations of risks we assess how much of the risk is mediated through other risks (step 24). For instance, all of the effect of high salt intake is mediated through elevated blood pressure and part of the risk of increased body-mass index is through elevated blood pressure, cholesterol or FPG.
14. Computation of YLLs, YLDs, and DALYs attributable to risk factors (steps 25a-25c): YLLs, YLDs and DALYs attributable to each risk factor are generated by multiplying population attributable fractions with disease estimates (steps 25a-c).

D. Diabetes morbidity estimation

The steps in the estimation of non-fatal diabetes burden or morbidity are shown in the following flowchart.

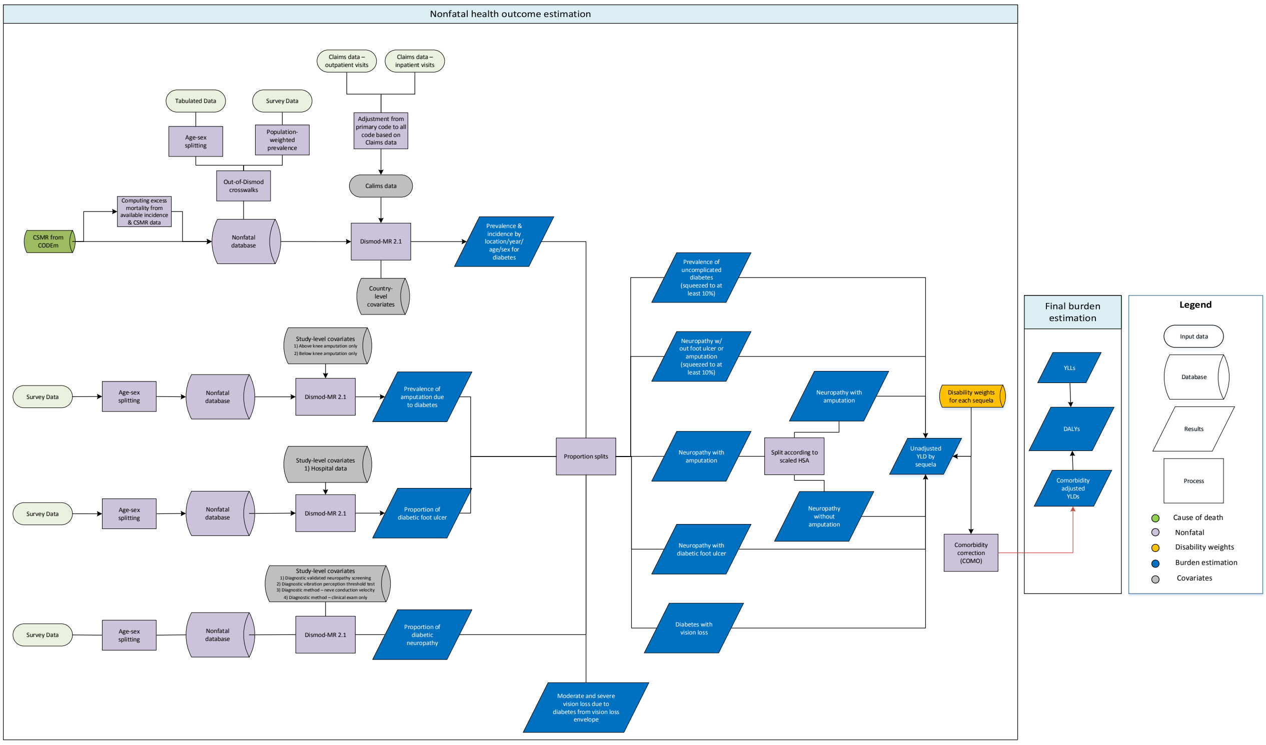

Data

To incorporate all available data related to population-representative estimates of diabetes, we accepted other measures of blood sugar such as haemoglobin A1c (HbA1c), oral glucose tolerance test, post prandial glucose (PPG) test to define diabetes and mean FPG in a population when data on diabetes was not available as data inputs.

The data inputs derived from estimates of diabetes in a representative population, estimates of mean FPG in a representative population, and individual-level data of FPG measured from surveys.

When a study reported both mean FPG and prevalence of diabetes, we used the prevalence of diabetes. Where possible, individual-level data from a cohort superseded any data described in a study. Individual-level data was collapsed and aggregated to produce estimates for each age group, sex, location, and year a survey is conducted.

To inform our estimates in data-sparse countries, we systematically tested a range of covariates and selected two covariates based on AIC and adjusted R^2. These included prevalence of obesity per location and lag-distributed income per capita (LDI).

Where possible, individual level data on diabetes estimates were extracted from survey microdata and these were collapsed across individuals and collapsed across demographic groupings to produce mean estimates in the standard GBD 5-year age-sex groups. If microdata were unavailable, information from survey reports or from literature were extracted along with any available measure of uncertainty including standard error, uncertainty intervals (UI), and sample size.

We perform several processing steps to the data in order to address sampling and measurement inconsistencies that will ensure that data are comparable across data sources and high FPG modelling efforts.

1. Small sample size: estimates in a sex and age group with a sample size <30 persons was considered a small sample size. In order to avoid small sample size problems that may bias estimates, data were collapsed into the next age group in the same study till the sample size reached at least 30 persons. The intent of collapsing the data is to preserve as much granularity between age groups as possible which determined whether the collapse occurred with a younger or older age group. If the entire study sample consisted of <30 persons and did not include a population-weight, the study was excluded from the modelling process. The estimates were re-calculated if case count and sample size were available or the population-weighted estimate was calculated when only sample size was available.

2. Time, age, and sex splitting:
   i. Time: Prior to modelling in DisMod, any study period that spanned more than 5 years was duplicated.
   ii. Age: Prior to modelling in DisMod, data provided in age groups wider than the GBD 5-year age groups were split using the global age pattern of diabetes mellitus from data that were in age groups less than 20-year age groups. Uncertainty was propagated by multiplying the standard error of the data performed by the square root of the number of splits performed.
   iii. Sex: Prior to modelling in DisMod, data that does not differentiate gender is split into male and female according to the global male to female ratio from data with sex-specific data. Uncertainty was propagated by multiplying the standard error of the data performed by the square root of the number of splits performed. Please see appendix pp 20 for description of uncertainty interval.

3. Mean FPG processing: For more details on how datapoints on mean FPG was processed, please refer to appendix pp 15-16 of this document.

4. Crosswalks
   i. Case-definition: we performed adjustments (crosswalks) to datapoints to standardise data to a reference definition: FPG >126 mg/dL (7 mmol/L) or on treatment.
      - Prevalence
        - Single-component: single component case definitions consisted of diabetes defined based on the level of only one biomarker (e.g., FPG, HbA1c).
          - FPG: we used an ensemble distribution to standardised the case definition of diabetes in surveys by estimating the prevalence of diabetes under different thresholds of FPG. We used individual-level measures of FPG in surveys of a representative population. This allowed us to capture the non-

systematic change in the proportion of population above different levels of FPG. We adjusted the datapoint by applying the ratio between FPG above 126 mg/dL and the case-definition used in the study. For more details on the approach used in the ensemble distribution, please see the GBD 2016 risk factors paper (Lancet 2017; 390: 1345–422).

- HbA1c: we assumed that HbA1c >6.5% was equivalent to FPG >126 mg/dL.

- Multi-component: multi-component case definition consisted of studies where more than 1 glucose test was used in the study to identify different segments of the population (e.g. FPG and PPG).
  - Multi-component that includes FPG >126 mg/dL: multi-component case definitions that consisted of FPG >126 mg/dL were assumed to be equivalent to the reference case definition FPG >126 mg/dL or treatment.
  - Multi-component that does not include FPG >126 mg/dL: data sources with case definitions that did not include FPG >126 mg/dL were excluded from the model.

- Non-prevalence measure

  Data from studies with non-prevalence measures (e.g., incidence, relative risk, excess mortality) were marked with the case definition and adjusted to the reference case definition within DisMod.

ii. Marketscan: data from MarketScan were included in the model and a study-level covariate was included in the model to adjust them. These datapoints were adjusted to the reference case definition within DisMod.

iii. Estimate prevalence of diabetes from mean FPG: we also used the ensemble distribution to estimate the prevalence of diabetes based on mean FPG in locations where data on prevalence of diabetes were not available. For more details on the approach used in the ensemble distribution, please see the GBD 2016 risk factors capstone paper (Lancet 2017; 390: 1345–422).

Modelling strategy

For GBD 2016, we estimated the overall prevalence of diabetes using DisMod MR- 2.1, a Bayesian metaregression. DisMod-MR produces estimates of the prevalence of diabetes for each age, sex, geographic location, and year. We also estimated amputation due to diabetes mellitus, diabetic neuropathy, and diabetic foot using DisMod. We then multiply all proportion draws from neuropathy/foot/amputation models by the parent diabetes model so that all estimates are in the same population- space.

Next, we squeeze (neuropathy + moderate vision loss + severe vision loss) to (90% of parent diabetes) prevalence if sum exceeds that 90%. This is to ensure that at least 10% of diabetes cases are uncomplicated for all draws. We then squeeze (amputation + foot ulcer) to (90% of neuropathy) prevalence if sum exceeds 90%. This is to ensure that at least 10% of diabetic neuropathy cases do not have foot ulcer or amputation for all draws. This treats foot ulcer and amputation as mutually exclusive categories by assuming a patient won't have both simultaneously.

From here, we calculate uncomplicated diabetes as the remainder of diabetes cases exclusive of neuropathy and vision loss. In addition, we estimate the prevalence of amputation due to diabetes is split into with and without treatment using scaled health system access (HSA) values. For diabetic amputation, we calculated a distribution of treated versus untreated amputation, defined as receiving a prosthetic or not. We first rescaled the IHME health system access estimates to be between 0 and 0.9, under the assumption that 10% of amputees will not receive a prosthetic, even in high income countries. We based this assumption on a retrospective study, which found that about 80% of patients following major lower extremity amputation were fitted with prostheses in the authors' institutions from 1978 to 1986 in the USA. We then performed a population- weighted average of this country- specific value to obtain a proxy for the proportion of amputees that receive a prosthetic by super region. Because these are rough estimates based on large assumptions, we applied confidence intervals of +/- 50% of the value to reflect our uncertainty.

The assumptions and covariates used for the modelling of diabetes mellitus and its sequelae follow.

Diabetes mellitus
We set values for the following: prior of 0 for remission for ages 0 to 14, prior of a maximum value of 0.01 for remission for ages 15 to 100, prior of a maximum value of 0.15 for excess mortality for all ages, prior of 0 for incidence for ages 0 to 1, and prior of a maximum value of 0.1 for incidence for ages 1 to 100.

Covariates	Parameter	Beta	Exponentiated beta
Sex	With-condition mortality rate	0.27 (-0.9 – 1.49)	1.31 (0.41 – 4.45)
LDI (I$ per capita)	Excess mortality rate	-0.24 (-0.25 – -0.22)	0.79 (0.78 – 0.80)
All MarketScan, year 2000	Prevalence	-0.48 (-0.53 – -0.43)	0.62 (0.59 – 0.65)
All MarketScan, year 2010	Prevalence	-0.17 (-0.21 – -0.11)	0.85 (0.81 – 0.90)
All MarketScan, year 2012	Prevalence	-0.15 (-0.2 – -0.091)	0.86 (0.82 – 0.91)
Obesity	Prevalence	2.76 (2.46 – 3.07)	15.79 (11.66 – 21.57)
Sex	Prevalence	0.17 (0.15 – 0.19)	1.18 (1.16 – 1.21)
Sex	Incidence	0.035 (-0.042 – 0.11)	1.04 (0.96 – 1.12)
Sex	Excess mortality rate	0.18 (0.15 – 0.20)	1.19 (1.16 – 1.23)
Sex	Cause-specific mortality rate	0.00030 (-0.0058 – 0.0059)	1.00 (0.99 – 1.01)

Our estimate of the age-standardised global prevalence of diabetes is slightly lower than the estimates reported previously by the NCD Risk Factor Collaboration (NCD-RisC) and International Diabetes Federation (IDF). IDF reported a prevalence for the year 2013 of 8.3% (7.2–11.3) at ages 20 to 80, compared to our estimate for 2016 of 6.0% (5.1–7.0) for the same age range and using the IDF method of age-standardisation (NCD-RisC: http://www.sciencedirect.com/science/article/pii/S0140673616006188
IDF: https://www.idf.org/component/attachments/attachments.html?id=1093&task=download.)
The NCD-RisC estimates of prevalence for ages over 18 for the year 2014 were 9.0% (7.2–11.1) in males and 7.9% (6.7–9.7) in females, compared to our 2016 estimates of 5.3% (4.5–6.2) and 4.9% (4.1–5.7), respectively. Several factors can explain the difference in estimates. We include a greater number of data sources but exclude surveys with self-reported diagnosis of diabetes unlike NCD-RisC. We also define the whole distribution of FPG and thus have a more accurate way of including surveys that report on FPG only in our diabetes disease model.

Amputation due to diabetes
We set values for the following: prior of 0 for incidence for ages 0 to 15, and prior of 0 for remission for all ages. We crosswalked the incidence of either above or below knee amputation only to the incidence of all amputations.

Covariates	Parameter	Beta	Exponentiated beta
Above knee amputation only	Incidence	-0.32 (-0.6 — -0.034)	0.72 (0.55 — 0.97)
Below knee amputation only	Incidence	-0.44 (-0.72 — -0.18)	0.64 (0.49 — 0.83)

Diabetic neuropathy
We set a value prior on the proportion of 0 from ages 0 to 1. We crosswalked data from studies using alternate diagnostic criteria using as reference studies which used the monofilament test as their diagnostic criteria.

Covariates	Parameter	Beta	Exponentiated beta
Diagnostic vibration perception threshold test	Proportion	-0.13 (-0.33 – 0.11)	0.88 (0.72 – 1.12)
Diagnostic method – nerve conduction velocity	Proportion	-0.25 (-0.5 – 0.029)	0.78 (0.61 – 1.03)
Diagnostic method – clinical exam only	Proportion	-0.044 (-0.27 – 0.21)	0.96 (0.76 – 1.24)
Diagnostic validated neuropathy scoring	Proportion	-0.021 (-0.23 – 0.20)	0.98 (0.79 – 1.23)

Diabetic foot ulcer
We set a value prior on the proportion of 0 from ages 0 to 10. We crosswalked data from studies investigating hospitalized patients only using as reference studies which captured all diabetic foot ulcers.

Covariates	Parameter	Beta	Exponentiated beta

Hospital data	Proportion	0.52 (0.13 – 0.87)	1.68 (1.14 – 2.38)

Disability weights

Severity splits and disability weights were determined for diabetes mellitus by the GBD disability weight survey assessment for diabetes mellitus. The table below illustrates the severity levels, lay descriptions, and associated disability weights:

**Severity level**	**Lay description**	**Disability weight (95% CI)**
Uncomplicated diabetes mellitus	Has a chronic disease that requires medication every day and causes some worry, but minimal interference with daily activities	0.049 (0.031–0.072)
Diabetic neuropathy	Has pain, tingling, and numbness in the arms, legs, hands, and feet. The person sometimes gets cramps and muscle weakness.	0.133 (0.089–0.187)
Diabetic neuropathy with diabetic foot	Has a sore on the foot that is swollen and causes some difficulty in walking.	a
Diabetic neuropathy with treated amputation	Has lost part of one leg, leaving pain and tingling in the stump. The person has an artificial leg that helps in moving around.	a
Diabetic neuropathy with untreated amputation	Has lost part of one leg, leaving pain and tingling in the stump. The person does not have an artificial leg, has frequent sores, and uses crutches.	a
Moderate vision loss due to diabetes mellitus	Has vision problems that make it difficult to recognize faces or objects across a room.	0.031 (0.019–0.049)
Severe vision loss due to diabetes mellitus	Has severe vision loss, which causes difficulty in daily activities, some emotional impact (for example worry), and some difficulty going outside the home without assistance.	0.184 (0.125–0.259)
Blindness due to diabetes mellitus	Is completely blind, which causes great difficulty in some daily activities, worry and anxiety, and great difficulty going outside the home without assistance.	0.187 (0.124–0.26)

[a] The disability weights are produced from a combination of two health states: neuropathy and diabetic foot/amputation.

E. Diabetes mortality estimation

The approach to cause of death estimation is shown in the following flowchart

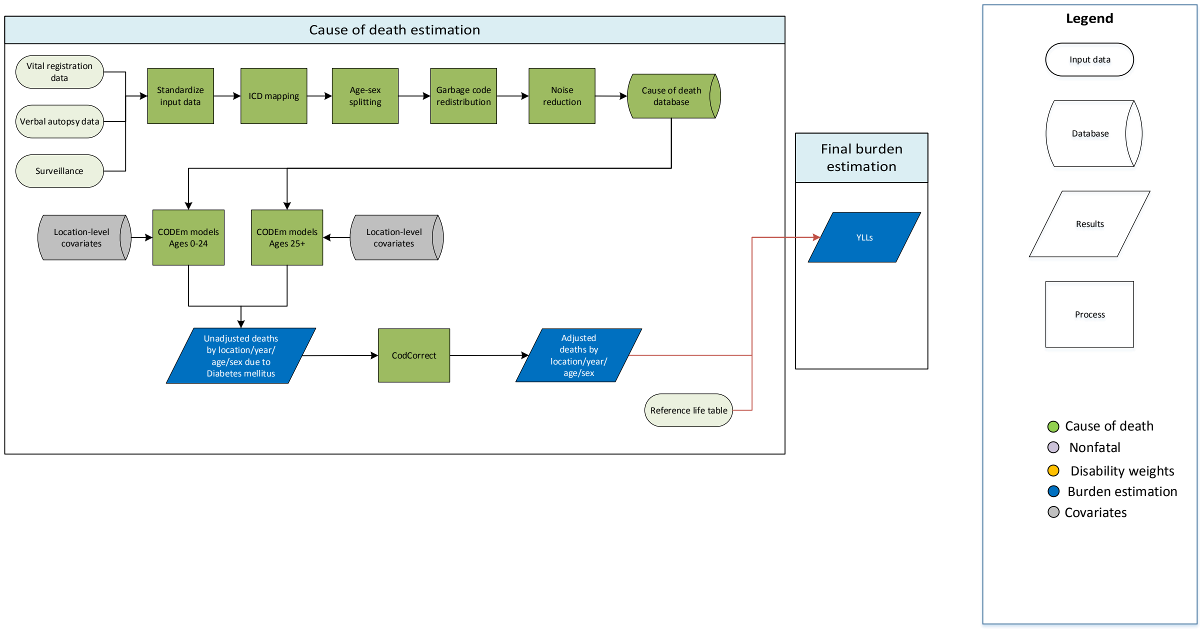

Data

The major data input to determine diabetes mortality in India was the Sample Registration System (SRS) cause of death data and some other studies. The SRS in India is operated by the Office of the Registrar General of India working under the Ministry of Home Affairs, Government of India. Cause of death data from SRS verbal autopsy covers 455,460 deaths from the rural and urban populations of every state of India from 2004 to 2013 in which physicians assigned the cause of death based on the information provided in the verbal autopsy interview of a person close to each deceased person. Using the 2001 census, 7597 geographic units, 4433 (58·4%) of which were rural, were sampled for the 2004–13 SRS to represent the population of each state and union territory of India, ultimately with a sample of 6·7 million people that was equivalent to 0·7% of India's population. The SRS cause of death data for 2004–06, 2007–09, and 2010–13 were provided for each state and union territory by the Office of the Registrar General of India for use in the state-level disease burden estimation. We used 2005, 2008, and 2012 as midpoint years for these three time periods. The inclusion of SRS 2004–13 data in this analysis offers a comprehensive picture of causes of death in India. In the absence of a fully functional vital registration system, verbal autopsy can provide reasonable population level cause of death distribution. (Lancet 2017; 390: 2437–2460).

Modelling strategy

Cause of death ensemble modelling (CODEm) is the framework used to model most cause- specific death rates in the GBD. It relies on four key components. First, all available data are identified and gathered to be used in the modelling process. Though the data may vary in quality, they all contain some signal of the true epidemiological process. Second, a diverse set of plausible models are developed to capture well-documented associations in the estimates. Using a wide variety of individual models to create an ensemble predictive model has been shown to outperform techniques using only a single model both in cause of death estimation and in more general prediction applications. Third, the out- of- sample predictive validity is assessed for all individual models, which are then ranked for use in the ensemble modelling stage. Finally, differently weighted combinations of individual models are evaluated to select the ensemble model with the highest out-of- sample predictive validity.

As many factors covary with a particular cause of death, a large range of plausible statistical models are developed for each cause. For the CODEm framework, four families of statistical models are developed using covariates. These are mixed effects linear models of the natural log of the death rate, mixed effects linear models of the logit of the cause fraction, spatiotemporal Gaussian process regression (ST-GPR) models of the log of the death rate, and ST-GPR of the logit of the cause fraction. All plausible relationships

between covariates and relevant cause are identified, and all possible permutations of selected covariates are tested in linear models where the logit cause fraction or log death rate is the response variable. Because we test all permutations of covariates, multicollinearity between covariates may produce implausible signs on coefficients or unstable coefficients. All models where the sign on the coefficient is in the direction expected based on the literature and where the coefficient is statistically significant at $p < 0.05$ are retained. We run covariate selection for both cause fractions and death rates and then create both mixed effects only and ST models for each set of covariates.

The performance of all component models and ensembles is evaluated using out-of-sample predictive validity tests. Thirty percent of the data are excluded from the initial model fits, and half of that (15% of total) is used to evaluate and rank component models and then build ensembles. Data are held out from the analysis using the pattern of missingness for each cause in the cause of death database. Out-of-sample predictive validity testing is repeated until stable model results have been obtained. The out-of-sample performance tests include the root mean squared error of the log of the cause-specific death rate, the direction of the trend in the prediction compared to the data, and the validity of the 95% uncertainty interval. For every model, we show the in-sample root mean squared error of the log death rates (RMSE) and the out-of-sample performance in the 15% of data not used in the model building process.

After component models are ranked on their out-of-sample predictive validity they are weighted based on their ranking and each component model contributes a portion to the final estimate. How much each submodel contributes is a function of its relative ranking as well as the value of psi chosen, which dictates that distribution of rankings.

Using the second half of the holdout data (15% of total), the differently weighted ensembles and different values of psi are tested using the same predictive validity metrics as the component models. For every model, we show the in-sample RMSE and the out-of-sample performance in the 15% of data not used in the model building process. The ensemble with the best average trend and RMSE is chosen as the final ensemble weighting scheme.

After a model weighting scheme has been chosen, each model contributes a number of draws proportional to its weight such that 1,000 draws are created. The mean of the draws is used as the final estimate for the CODEm process and 95% UI are created from the 0.025 and 0.975 quantiles of the draws. The final assessment of ensemble model performance is the validity of the UIs; ideally, the 95% UI for a model would capture 95% of the data out-of-sample. Higher coverage suggests that UIs are too large and lower than 95% suggest UIs are too narrow.

We used a slight variation on the standard CODEm approach to model deaths from diabetes mellitus. Since deaths in younger age groups are almost exclusively due to Type 1 diabetes while deaths in older ages are primarily due to Type 2, we used two models to estimate overall diabetes deaths. We reviewed the cause-fraction of deaths due to Type 1 and Type 2 diabetes at the global, super region, and regional level. We selected a conservative estimate of 25 years; one model is for deaths in 0-25 year olds and the second model is for deaths in 25+ year olds.

CODEm models estimate the individual cause-level mortality without taking into account the all-cause mortality. GBD uses the CodCorrect algorithm to ensure that all individual causes add up to the all-cause mortality. After generating underlying cause of death estimates and accompanying uncertainty, this algorithm combines these models into estimates that are consistent with the levels of all-cause mortality estimated for each age-sex-year-location group. Using 1000 draws from the posterior distribution of each cause and 1000 draws from the posterior distribution of the estimation of all-cause mortality, CoDCorrect rescales the sum of cause-specific estimates to equal the draws from the all cause distribution. Further details of CodCorrect algorithm can be found in the appendix to the GBD 2016 cause of death capstone paper (Lancet 2017; 390: 1151–210).

The following list of covariates were included in the models:

- Education years per-capita
- A composite score that approximates access to and quality of personal healthcare (Healthcare Access and Quality Index)
- Lag distributed GDP per capita in base 2010 international dollars
- Estimated national availability of animal fat expressed as kilocalories per capita
- Mean diabetes FPG (mmol/L) by age group

- Age-standardised prevalence of diabetes
- Age-standardised mean body-mass index for adults ages 20+ (separate by sex)
- Mean serum total cholesterol (mmol/L) for individuals above age 25
- Mean systolic blood pressure (mmHg) for individuals above age 25
- Estimated energy adjusted national availability of fruits expressed in grams per person per day
- Estimated energy adjusted national availability of vegetables expressed in grams per person per day
- Estimated energy adjusted national availability of whole grains expressed in grams per person per day
- Estimated national availability of dietary energy expressed in kilocalories per person per day

F. Estimation of major risk factors for diabetes

The approach used in GBD 2016 for comparative risk assessment to estimate population attributable fractions for risk factors is shown in the following flowchart.

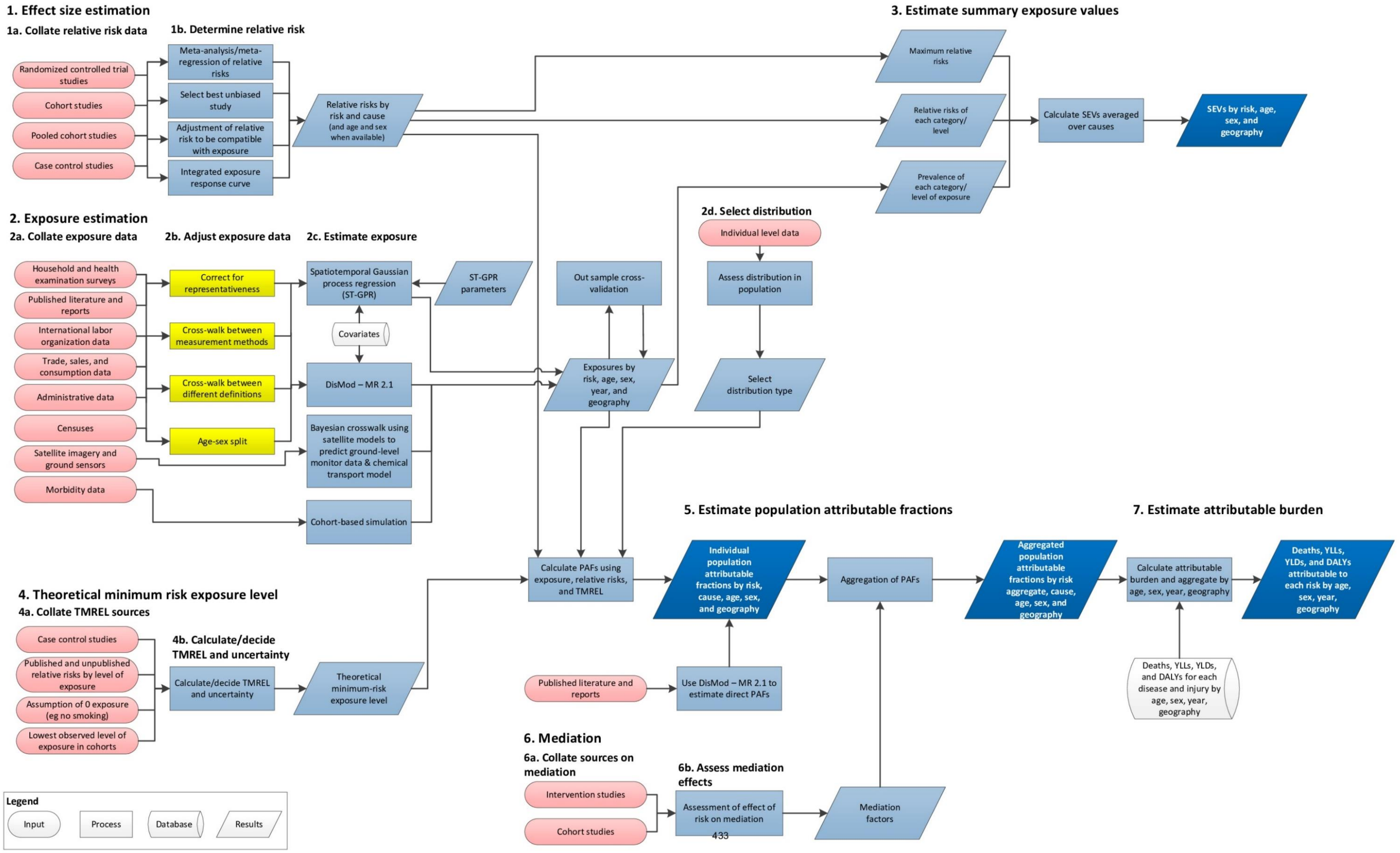

GBD is Global Burden of Disease. SEVs is summary exposure values. TMREL is theoretical minimum- risk exposure level. PAFs is population attributable fractions. YLLs is years of life lost. YLDs is years lived with disability. DALYs is disability-adjusted life- years. Ovals represent data inputs, rectangular boxes represent analytical steps, cylinders represent databases, and parallelograms represent intermediate and final results.

We describe details of two major risk factors related to diabetes, i.e. high FPG and high body-mass index. Description of other risk factors can be found in the GBD 2016 risk factor paper (Lancet 2017; 390: 1345–422).

F.1. High fasting plasma glucose

FPG level is used to define diabetes (FPG more than 126 mg/dL or 7 mmol/L), and FPG is also a risk factor for other disease conditions.

For the purpose of attributing disease burden to FPG, the theoretical minimum risk exposure level (TMREL) for FPG was estimated to range from 81 to 97 mg/dL or 4.5 to 5.4 mmol/L (mean 90 mg/dL or 5 mmol/L) as a risk of chronic kidney disease, ischaemic heart disease, stroke, and peripheral vascular disease. Above this FPG level, the risk was considered continuous. Based on the relative risks obtained from meta-analysis, FPG level more than 126 mg/dL (7 mmol/L) was considered as a categorical risk for tuberculosis, liver cancer, pancreatic cancer, ovarian cancer, colorectal cancer, bladder cancer, lung cancer, breast cancer, glaucoma, cataract, and Alzheimer’s disease and other dementias. This was calculated by taking the person-year weighted average of the levels of FPG that were associated with the lowest risk of mortality in the pooled analyses of prospective cohort studies. To include the uncertainty in the TMREL, we took a random draw from the uniform distribution of the interval between 4.5 mmol/L and 5.4 mmol/L each time the population attributable burden was calculated.

Morbidity and mortality directly caused by diabetes was considered directly attributable to FPG.

The steps in the estimation of disease burden attributable to high FPG are shown in the following flowchart.

High fasting plasma glucose

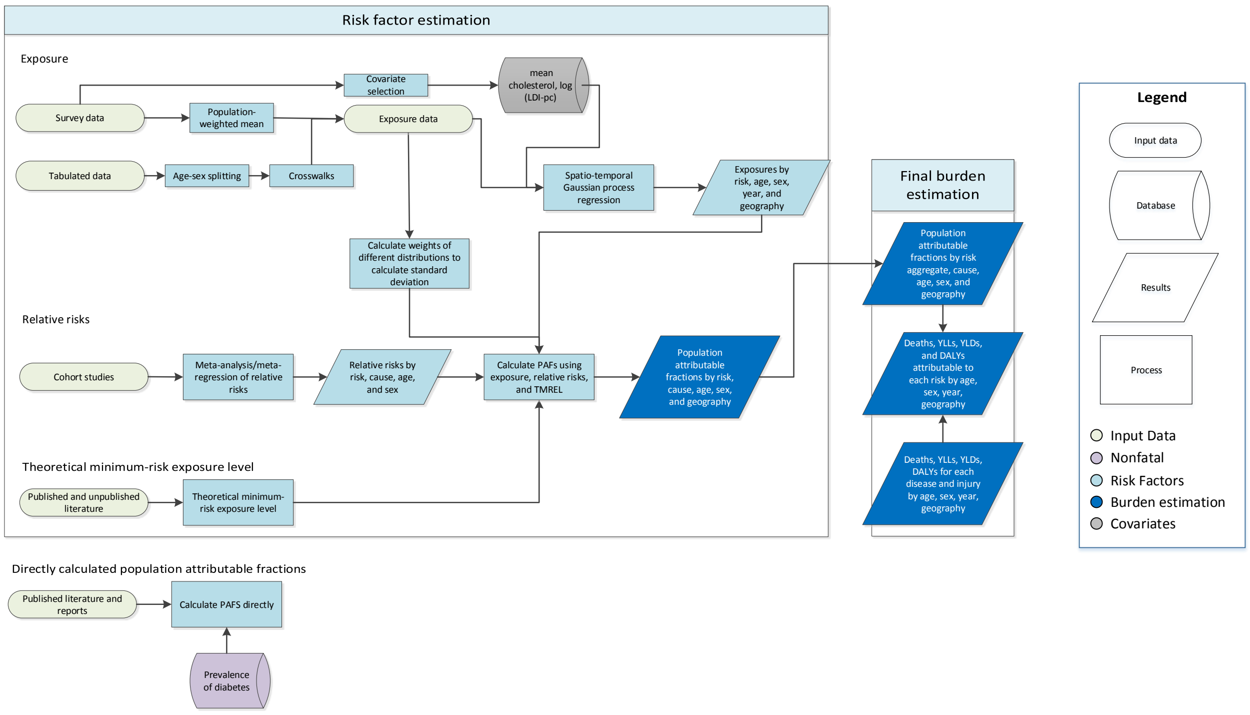

Data

The data inputs derived from estimates of mean FPG in a representative population, individual-level data of FPG measured from surveys, and estimates of diabetes prevalence in a representative population. Data sources that did not report mean FPG or prevalence of diabetes were excluded from analysis. When a study reported both mean FPG and prevalence of diabetes, we used the mean FPG for exposure estimates. Where possible, individual-level data superseded any data described in a study. Individual-level data was collapsed and aggregated to produce estimates for each age group, sex, location, and year a survey is conducted.

We perform several processing steps to the data in order to address sampling and measurement inconsistencies that will ensure the data are comparable.

Small sample size: estimates in a sex and age group with a sample size <30 persons was considered a small sample size. In order to avoid small sample size problems that may bias estimates, data were collapsed into the next age group in the same study till the sample size reached at least 30 persons. The intent of collapsing the data is to preserve as much granularity between age groups as possible which determined whether the collapse occurred with a younger or older age group. If the entire study sample consisted of <30 persons and did not include a population-weight, the study was excluded from the modelling process. The estimates were re-calculated if case count and sample size were available or the population-weighted estimate was calculated when only sample size was available.

Time, age, and sex splitting: for more details on how data points on mean FPG was processed, please refer to appendix pp 8 of this document.

Crosswalks: we predicted mean FPG from diabetes prevalence using an ensemble distribution. We characterized the distribution of FPG using individual-level data. For more details on the ensemble distribution, please see the GBD 2016 risk factors paper (Lancet 2017; 390: 1345–422). Before predicting mean FPG from prevalence of diabetes, we ensured that the prevalence of diabetes was based on the reference case definition: FPG > 126 mg/dL (7 mmol/L) or on treatment.

Modelling strategy

Exposure estimates were produced from 1980 to 2016 for each national and subnational location, sex, and for each 5-year age group starting from 25+. We used ST-GPR framework to model the mean FPG at the location-, year-, age-, sex- level.

FPG is frequently tested or reported in surveys aiming at assessing the prevalence of diabetes mellitus. In these surveys, the case definition of diabetes may include both a glucose test and questions about treatment for diabetes; people with positive history of diabetes treatment are generally excluded from the FPG test. Thus, the mean FPG in these surveys may not represent the mean FPG in the entire population. To address this limitation, using the data from the surveys reporting mean FPG in the entire population, we estimated a regression-based correction factor and adjusted the mean FPG to account for diabetics in the population. We also used an ensemble distribution to characterize the distribution of FPG in the population and developed an optimization function to estimate the standard deviation based on mean FPG and prevalence of diabetics.

To inform our estimates in data-sparse countries, we systematically tested a range of covariates and selected two covariates based on AIC and adjusted R2. These included prevalence of obesity and lag distributed income per capita (LDI).

Mean FPG was estimated using a mixed-effects linear regression, run separately by sex:

$$\log(FPG_{c,a,t}) = \beta_0 + \beta_1 \log(LDI)_{c,t} + \beta_2 Poverweight_{c,a,t} + \sum_{k=2}^{16} \beta_k I_{A[a]} + \alpha_s + \alpha_r + \alpha_c + \epsilon_{c,a,t}$$

where $\log(LDI)_{c,t}$ is the log of the lag-distributed income, $P\ overweight_{c,a,t}$ is the prevalence of overweight, $I_{A[a]}$ is an indicator variable for a fixed effect on a given 5-year age group, and α_s α_r α_c are random effects at the super-region, region, country, and subnational level, respectively.

The estimates were then propagated through the ST-GPR framework to obtain 1000 draws for each location, year, age, and sex.

We used Dismod-MR 2.1 to pool effect sizes from included studies and generate a dose-response curve for each of the outcomes associated with high FPG. The tool enabled us to incorporate random effects across studies and include data with different age ranges. RRs were used universally for all countries and the meta-regression only helped to pool the three major sources and produce RRs with uncertainty and covariance across ages taking into account the uncertainty of the data points.

F.2. High body-mass index

For the purpose of attributing disease burden to high body-mass index (BMI), the theoretical minimum risk exposure level for BMI in adults (ages 20+ years) was estimated to range between 20 to 25 kg/m^2 (mean 22.5 kg/m^2) based on the BMI level that was associated with the lowest risk of all-cause mortality in prospective cohort studies, and for children (age up to 19 years) was based on International Obesity Task Force (IOTF) cut-offs for normal weight. The risk-outcome pairs to attribute burden of specific conditions to high BMI were defined based on the strength of available evidence supporting a causal effect of BMI in meta-analysis. To include the uncertainty in the TMREL, we took a random draw from the uniform distribution of the interval between 20 and 25 kg/m^2 each time the population attributable burden was calculated.

The steps in the estimation of disease burden attributable to high BMI are shown in the following flowchart for adults and children.

Adult (Ages 20+) High Body-Mass Index: Data and Model Flow Chart

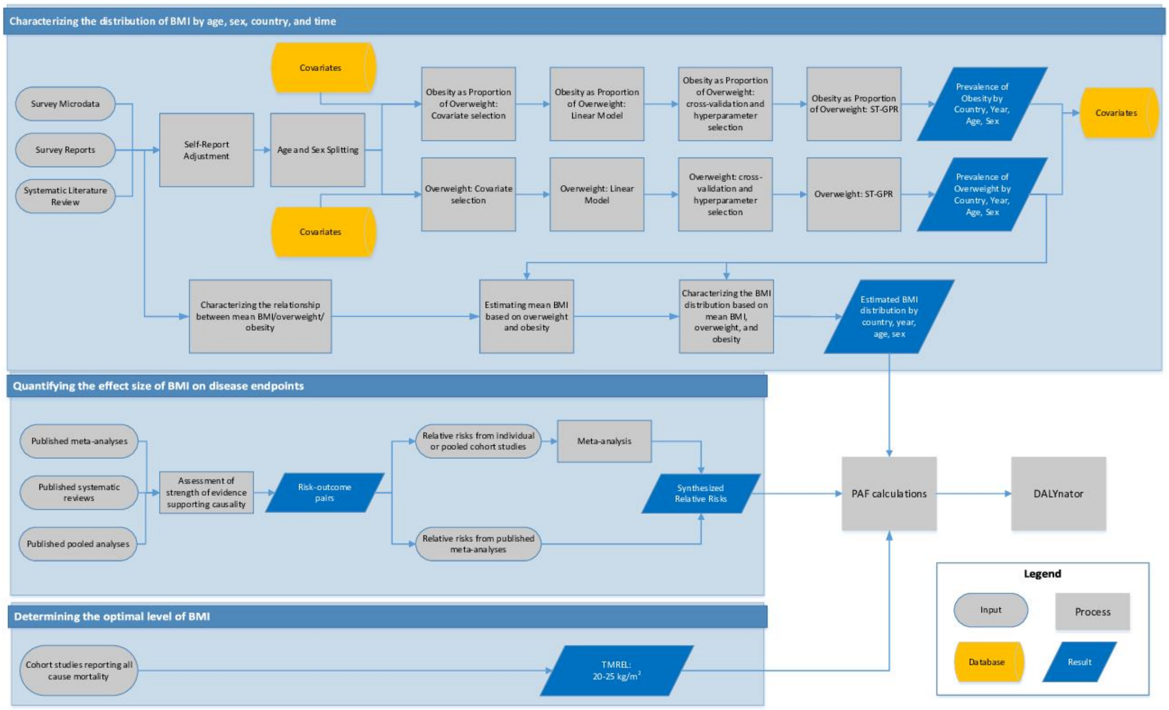

Childhood (Ages 2-19) High Body-Mass Index: Data and Model Flow Chart

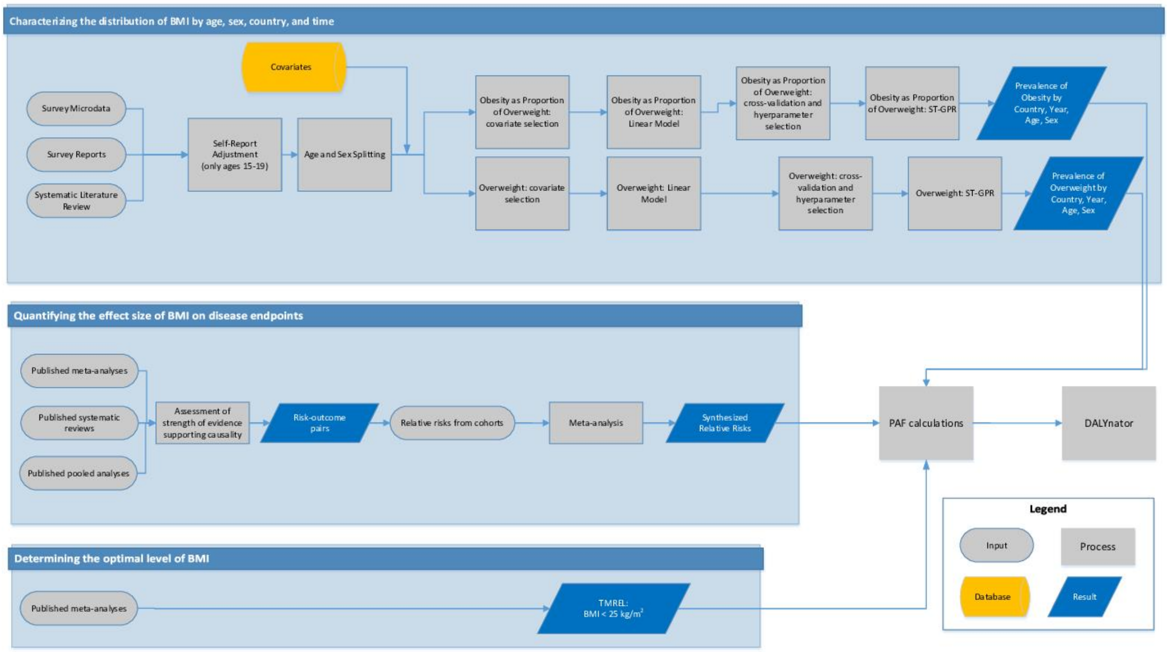

Data

We systematically searched Medline to identify studies providing nationally or subnationally representative estimates of overweight prevalence, obesity prevalence, or mean BMI. We included representative studies providing data on mean BMI or prevalence of overweight or obesity among adults or children. For adults, studies were included if they defined overweight as BMI≥25 kg/m^2 and obesity as BMI≥30 kg/m^2, or if estimates using those cut-offs could be back-calculated from reported categories. For children, studies were included if they used IOTF standards to define overweight and obesity thresholds. Studies were excluded if using non-random samples (e.g., case-control studies or convenience samples); conducted among specific subpopulations (e.g., pregnant women, racial or ethnic minorities, immigrants, or individuals with specific diseases); using alternative methods to assess adiposity (e.g., waist-circumference, skin-fold thickness, or hydrodensitometry); having sample sizes of less than 20 per age-sex group; or providing inadequate information on any of the inclusion criteria.

Where individual-level survey data were available, we computed mean BMI using weight and height and then used BMI to determine the prevalence of overweight and obesity. For individuals aged over 18 years, we considered them to be overweight if their BMI was greater than or equal to 25 kg/m^2, and obese if their BMI was greater than or equal to 30 kg/m^2. For individuals aged 2-18 years, we used monthly IOTF cut-offs to determine overweight and obese status when age in months was available. When only age in years was available, we used the cut-off for the 6 month of that year. Individuals who were obese were also considered to be overweight. We excluded studies using the World Health Organization (WHO) standards or country-specific cut-offs to define childhood overweight and obesity. At the individual-level, we considered BMI<10 kg/m^2 and BMI>70 kg/m^2 to be biologically implausible and excluded those observations.

The rationale for choosing to use the IOTF cut-offs over the WHO standards was that the IOTF cut-offs provide consistent child-specific standards for ages 2-18 derived surveys covering multiple countries. On the other hand, the WHO growth standards apply to children under 5 and the WHO growth reference applies to children ages 5-19. The WHO growth reference for children ages 5-19 was derived from United States data which is less representative than the multinational data used by IOTF. Additionally, the switch between references at age 5 can produce artificial discontinuities. Given that we estimate global childhood overweight and obesity for ages 2-19 (with ages 19 using standard adult cut-offs), the IOTF cut-offs were preferable. Additionally, we found that IOTF cut-offs were more commonly used in scientific literature covering childhood obesity.

From report and literature data, we extracted data on mean BMI, prevalence of overweight, and prevalence of obesity, measures of uncertainty for each, and sample size, by the most granular age and sex groups available. Additionally, we extracted the same study-level covariates as were extracted from microdata (measurement, urbanicity, and representativeness), as well as location and year.

We included both measured and self-reported data. Of the 72.6 million person-years of data globally, 18.8 million (26%) were self-reported. We tested for bias in self-report data compared to measured data, which is considered to be the gold-standard. There was no clear direction of bias for children ages 2-14, so for data for overweight prevalence, obesity prevalence, and mean BMI using the following nested hierarchical mixed-effects regression models, fit using restricted maximum likelihood separately by sex:

$$\text{logit}(\text{overweight})_{c,a,t} = \beta_0 + \beta_1 m + \sum_{k=2}^{19} \beta_k I_{A[a]} + \sum_{l=20}^{55} \beta_l\, I_{A[a]} I_{M[m]} + \alpha_s + \alpha_s m + \alpha_r + \alpha_r m + \alpha_c + \alpha_c m + \alpha_t + \alpha_t m + \epsilon_{c,a,t}$$

$$\text{logit}(\text{obesity})_{c,a,t} = \beta_0 + \beta_1 m + \sum_{k=2}^{19} \beta_k I_{A[a]} + \sum_{l=20}^{55} \beta_l\, I_{A[a]} I_{M[m]} + \alpha_s + \alpha_s m + \alpha_r + \alpha_r m + \alpha_c + \alpha_c m + \alpha_t + \alpha_t m + \epsilon_{c,a,t}$$

$$\log(\text{BMI})_{c,a,t} = \beta_0 + \beta_1 m + \sum_{k=2}^{19} \beta_k I_{A[a]} + \sum_{l=20}^{55} \beta_l\, I_{A[a]} I_{M[m]} + \alpha_s + \alpha_s m + \alpha_r + \alpha_r m + \alpha_c + \alpha_c m + \alpha_t + \alpha_t m + \epsilon_{c,a,t}$$

Where m is a fixed effect on measurement (binary, either measured (1) or self-report (0)), IA[a] is an indicator variable for specific age group A, IA[a]IM[m] is an interaction term between age and measurement, αs, αr, and αc are random effects at the super region, region, country, and subnational level respectively, and αt is a random effect by time-period (1980-1989, 1990-1999, 2000-2009, 2010-2016). Random effects at the country- or state-level and time-period level were used to fit the models, but were taken as noise and were not used in adjustment of self-reported data. We propagated the uncertainty in the self-report adjustment model by adding the variance of each of the regression coefficients used in adjustment to the data variance in delta-transformed space. After

adjustment, regressions confirmed that self-reported data was no longer significantly different from measured data.

Modelling strategy

After adjusting for self-report bias and splitting aggregated data into 5-year age-sex groups, we used ST-GPR to estimate the prevalence of overweight and obesity.

The linear model, which when added to the smoothed residuals forms the mean prior for GPR is as follows:

$$\mathrm{logit(overweight)}_{c,a,t} = \beta_0 + \beta_1 \mathrm{energy} c,t + \beta_2 \mathrm{SDI} c,t + \beta_3 \mathrm{vehicles} c,t + \beta_4 \mathrm{agriculture} + \sum_{k=5}^{22} \beta_k I_{A[a]} + \alpha s + \alpha r + \alpha c$$

$$\mathrm{logit(obesity/overweight)}_{c,a,t} = \beta_0 + \beta_1 \mathrm{energy} c,t + \beta_2 \mathrm{SDI} c,t + \beta_3 \mathrm{vehicles} c,t + \sum_{k=4}^{22} \beta_k I_{A[a]} + \alpha s + \alpha r + \alpha c$$

where energy is ten-year lag-distributed energy consumption per capita, Socio-demographic Index (SDI) is a composite index of development including lag-distributed income per capita, education, and fertility, vehicles is the number of two or four-wheel vehicles per capita, and agriculture is the proportion of the population working in agriculture. $I_{A[a]}$ is a dummy variable indicating specific age group A that the prevalence point captures, and α_s, α_r, and α_c are super region, region, country, and subnational random intercepts, respectively. Random effects were used in model fitting but were not used in prediction.

We tested all combinations of the following covariates to see which performed best in terms of in-sample AIC for the overweight linear model and the obesity as a proportion of overweight linear model: ten-year lag distributed energy per capita, proportion of the population living in urban areas, SDI, lag-distributed income per capita, educational attainment (years) per capita, proportion of the population working in agriculture, grams of sugar adjusted for energy per capita, grams of sugar not adjusted for energy per capita, and the number of two or four-wheeled vehicles per capita. We selected these candidate covariates based on theory as well as reviewing covariates used in other publications. The final linear model was selected based on: 1) if the direction of covariates matched what is expected from theory, 2) all the included covariates were significant, and 3) minimizing in-sample AIC. The covariate selection process was performed using the dredge package in R.

We used different space weights by data density category: locations with 0-4 years covered by data used a space weight of 0.7, locations with 5-9 years covered by data used a space weight of 0.9, locations with 10-19 years covered by data used a space weight of 0.95, and locations with more than 20 years covered by data used a space weight of 0.99. The other parameters were consistent across data-density levels: age weight = 1.2 for overweight and age weight = 1.4 for obesity, time weight = 1, and scale = 10. The GPR amplitude was calculated at the region level.

To estimate the mean BMI for adults in each country or state, age, sex, and time period 1980-2016, we first used the following nested hierarchical mixed-effects model, fit using restricted maximum likelihood on data from sources containing estimates of all three indicators (prevalence of overweight, prevalence of obesity, and mean BMI), in order to characterize the relationship between overweight, obesity, and mean BMI:

$$\log(\mathrm{BMI}_{c,a,s,t}) = \beta_0 + \beta_1 \mathrm{ow}_{c,a,s,t} + \beta_2 \mathrm{ob}_{c,a,s,t} + \beta_3 \mathrm{sex} + \sum_{k=4}^{20} \beta_k I_{A[a]} + \alpha_s(1 + \mathrm{ow}_{c,a,s,t} + \mathrm{ob}_{c,a,s,t}) + \alpha_r(1 + \mathrm{ow}_{c,a,s,t} + \mathrm{ob}_{c,a,s,t}) + \alpha_c(1 + \mathrm{ow}_{c,a,s,t} + \mathrm{ob}_{c,a,s,t}) + \epsilon_{c,a,s,t}$$

where $\mathrm{ow}_{c,a,s,t}$ is the prevalence of overweight in country c, age a, sex s, and year t, $\mathrm{ob}_{c,a,s,t}$ is the prevalence of obesity in country c, age a, sex s, and year t, sex is a fixed effect on sex, $I_{A[a]}$ is an indicator variable for age, and α_s, α_r, and α_c are random effects at the super region, region, country, and subnational, respectively. The model was run in Stata 13.

We applied 1,000 draws of the regression coefficients to the 1,000 draws of overweight prevalence and obesity prevalence produced through ST-GPR to estimate 1,000 draws of mean BMI for each country or state, year, age, and sex. This approach ensured that overweight prevalence, obesity prevalence, and mean BMI were correlated at the draw level and uncertainty was propagated.

We used the ensemble distribution approach in which we fit ensemble weights by source and sex, with source- and sex-specific weights averaged across all sources included to produce the final global weights. The ensemble weights were fit on measured microdata. The final ensemble weights were: exponential = 0.002, gamma =

0.028, inverse gamma = 0.085, log logistic = 0.187, gumbel = 0.220, inverse Weibull = 0.141, Weibull = 0.011, lognormal = 0.058, normal = 0.012, beta = 0.136, mirror gamma = 0.008, and mirror gumbel = 0.113.

One thousand draws of BMI distributions for each location, year, age group, and sex estimated were produced by fitting an ensemble distribution using 1,000 draws of estimated mean BMI, 1,000 draws of estimated standard deviation, and the ensemble weights. Estimated standard deviation was produced by optimizing a standard deviation to fit estimated overweight prevalence draws and estimated obesity prevalence draws.
We used Dismod-MR 2.1 to pool effect sizes from included studies and generate a dose-response curve for each of the outcomes associated with high body mass index. The tool enabled us to incorporate random effects across studies and include data with different age ranges. RRs were used universally for all countries and the meta-regression only helped to pool the three major sources and produce RRs with uncertainty and covariance across ages taking into account the uncertainty of the data points.

G. Uncertainty intervals

Point estimates for each quantity of interest were derived from the mean of the draws, while 95% uncertainty intervals were derived from the 2.5th and 97.5th percentiles of the 1000 draw level values. Uncertainty in the estimation is attributable to sample size variability within data sources, different availability of data by age, sex, year, or location, and cause specific model specifications. We determined UIs for components of cause-specific estimation based on 1000 draws from the posterior distribution of cause specific mortality by age, sex, and location for each year included in the GBD 2016 analysis. Similarly, for non-fatal estimates if there was a change in disease estimates between locations or over time that was in the same direction in more than 950 of the 1000 samples we report it as significant. With this approach, uncertainty could be quantified and propagated into the final quantities of interest.

2. GBD 2016 India data inputs for diabetes mortality, morbidity, risk factors, and covariates

Aarhus University, Addiction Switzerland Research Institute, Alcohol Research Group, Public Health Institute, Centre for Addiction and Mental Health, Centre for Alcohol Policy Research, Turning Point Alcohol and Drug Centre, Kettil Bruun Society for Social and Epidemiological Research on Alcohol, University of North Dakota. India - Karnataka Gender, Alcohol and Culture: An International Study (GENACIS) 2003.

Agrawal RP, Ola V, Bishnoi P, Gothwal S, Sirohi P, Agrawal R. Prevalence of micro and macrovascular complications and their risk factors in type-2 diabetes mellitus. J Assoc Physicians India. 2014; 62(6): 504–8.

Ali MK, Bhaskarapillai B, Shivashankar R, Mohan D, Fatmi ZA, Pradeepa R, Masood Kadir M, Mohan V, Tandon N, Narayan KM, Prabhakaran D, CARRS investigators. Socioeconomic status and cardiovascular risk in urban South Asia: The CARRS Study. Eur J Prev Cardiol. 2016; 23(4): 408-19.

Anjana RM, Deepa M, Pradeepa R, Mahanta J, Narain K, Das HK, et al. Prevalence of diabetes and prediabetes in 15 states of India: results from the ICMR–INDIAB population-based cross-sectional study. Lancet Diabetes Endocrinol. 2017; 5(8):585–96.

Anjana RM, Pradeepa R, Das AK, Deepa M, Bhansali A, Joshi SR, et al. Physical activity and inactivity patterns in India - results from the ICMR-INDIAB study (Phase-1) (ICMR-INDIAB-5). Int J Behav Nutr Phys Act. 2014 Feb 26; 11(1):26.

Anjana RM, Pradeepa R, Deepa M, Datta M, Sudha V, Unnikrishnan R, Bhansali A, Joshi SR, Joshi PP, Yajnik CS, Dhandhania VK, Nath LM, Das AK, Rao PV, Madhu SV, Shukla DK, Kaur T, Priya M, Nirmal E, Parvathi SJ, Subhashini S, Subashini R, Ali MK, Mohan V. Prevalence of diabetes and prediabetes (impaired fasting glucose and/or impaired glucose tolerance) in urban and rural India: phase I results of the Indian Council of Medical Research-INdia DIABetes (ICMR-INDIAB) study. Diabetologia. 2011; 54(12): 3022-7.

Ashok S, Ramu M, Deepa R, Mohan V. Prevalence of neuropathy in type 2 diabetic patients attending a diabetes centre in South India. J Assoc Physicians India. 2002; 546–50.

Balagopal P, Kamalamma N, Patel TG, Misra R. A community-based participatory diabetes prevention and management intervention in rural India using community health workers. Diabetes Educ. 2012; 38(6): 822-34.

Bansal D, Gudala K, Muthyala H, Esam HP, Nayakallu R, Bhansali A. Prevalence and risk factors of development of peripheral diabetic neuropathy in type 2 diabetes mellitus in a tertiary care setting. J Diabetes Investig. 2014; 5(6): 714–21.

Beegom R. Diet, central obesity and prevalence of hypertension in the urban population of South India. Int J Cardiol. 1995; 51(2): 183-91.

Bhagyalaxmi A, Atul T, Shikha J. Prevalence of risk factors of non-communicable diseases in a District of Gujarat, India. J Health Popul Nutr. 2013; 31(1): 78-85.

Bhansali A, Dhandania VK, Deepa M, Anjana RM, Joshi SR, Joshi PP, Madhu SV, Rao PV, Subashini R, Sudha V, Unnikrishnan R, Das AK, Shukla DK, Kaur T, Mohan V, Pradeepa R. Prevalence of and risk factors for hypertension in urban and rural India: the ICMR-INDIAB study. J Hum Hypertens. 2015; 29(3): 204–9.

Bharati DR, Pal R, Kar S, Rekha R, Yamuna TV, Basu M. Prevalence and determinants of diabetes mellitus in Puducherry, South India. J Pharm Bioallied Sci. 2011; 3(4): 513-8.

Bharati DR, Pal R, Rekha R, Yamuna TV. Evaluation of the burden of type 2 diabetes mellitus in population of Puducherry, South India. Diabetes Metab Syndr. 2011; 5(1): 12-6.

Bharati S, Pal M, Bhattacharya BN, Bharati P. Prevalence and causes of chronic energy deficiency and obesity in Indian women. Hum Biol. 2007; 79(4): 395-412.

Biswas M, Manna CK. Prevalence of hypertension and sociodemographic factors within the Scheduled Caste community of the District Nadia, West Bengal, India. High Blood Press Cardiovasc Prev. 2011; 18(4): 179–85.

Cardiological Society of India. India - Kerala Coronary Artery Disease Risk Factors Prevalence Study 2011. [Data shared for this analysis]

Centers for Disease Control and Prevention (CDC), World Health Organization (WHO). India - Kerala Global Youth Tobacco Survey 2004. Atlanta, United States: Centers for Disease Control and Prevention (CDC).

Central Board of Secondary Education, Department of School Education and Literacy, Ministry of Human Resource Development, Government of India, Centers for Disease Control and Prevention, World Health Organization (WHO). India Global School-Based Student Health Survey 2007. Geneva, Switzerland: WHO; 2007.

Centre for Chronic Disease Control, All India Institute of Medical Sciences, Madras Diabetes Research Foundation, Public Health Foundation of India, Emory University, Aga Khan University. Center for Cardio-Metabolic Risk Reduction in South Asia Surveillance Baseline Survey 2010-2011. [Data shared for this analysis]

Centre for Chronic Disease Control, All India Institute of Medical Sciences, Madras Diabetes Research Foundation, Public Health Foundation of India, Emory University, Aga Khan University. Center for Cardio-Metabolic Risk Reduction in South Asia Surveillance Follow-up Survey 2013. [Data shared for this analysis]

Centre for Chronic Disease Control, Indian Council of Medical Research. India Diet and Lifestyle Interventions for Hypertension Risk reduction through Anganwandi Workers and Accredited Social Health Activists Baseline Study Dataset 2013-2014. [Data shared for this analysis]

Centre for Chronic Disease Control. India Prevalence of Coronary Heart Disease and its Risk Factors in Residents of Urban and Rural Areas of NCR Survey Dataset 2010-2012. [Data shared for this analysis]

Centre for Chronic Disease Control. India Prevalence of Coronary Heart Disease and its Risk Factors in Residents of Urban and Rural Areas of NCR Survey Report 2010-2012. New Delhi, India: Centre for Chronic Disease Control.

Christian Medical College - Vellore, MRC Epidemiology Resource Center, University of Southampton. India - Vellore Birth Cohort Study 1998-2002. [Data shared for this analysis]

Christian Medical College - Vellore. India Prevalence of Risk Factors for Non-Communicable Diseases in Rural and Urban Tamil Nadu 2010-2012. [Data shared for this analysis]

Daniel CR, Prabhakaran D, Kapur K, Graubard BI, Devasenapathy N, Ramakrishnan L, George PS, Shetty H, Ferrucci LM, Yurgalevitch S, Chatterjee N, Reddy KS, Rastogi T, Gupta PC, Mathew A, Sinha R. A cross-sectional investigation of regional patterns of diet and cardio-metabolic risk in India. Open Nutr J. 2011; 10(1): 12.

Deedwania PC, Gupta R, Sharma KK, Achari V, Gupta B, Maheshwari A, Gupta A. High prevalence of metabolic syndrome among urban subjects in India: a multisite study. Diabetes Metab Syndr. 2014; 8(3): 156–61.
Deepa M, Anjana RM, Manjula D, Narayan KMV, Mohan V. Convergence of prevalence rates of diabetes and cardiometabolic risk factors in middle and low income groups in urban India: 10-year follow-up of the Chennai Urban Population Study. J Diabetes Sci Technol. 2011; 5(4): 918–27.
Deepa M, Grace M, Binukumar B, Pradeepa R, Roopa S, Khan HM, et al. High burden of prediabetes and diabetes in three large cities in South Asia: The Center for Cardio-metabolic Risk Reduction in South Asia (CARRS) Study. Diabetes Res Clin Pract. 2015 Nov; 110(2):172–82.
Department of Health and Family Welfare, Government of Punjab, Postgraduate Institute of Medical Education & Research Chandigarh, University of Michigan. India - Punjab Noncommunicable Disease Risk Factors Survey 2014-2015. [Data shared for this analysis]
Department of Women and Child Development, Ministry of Human Resource Development, Government of India, United Nations Children's Fund (UNICEF) - India Country Office. India Summary Report on the Multiple Indicator Cluster Survey 2000. New Delhi, India: UNICEF India Country Office; 2000.
Desai, Sonalde, Reeve Vanneman, National Council of Applied Economic Research, University of Michigan. India Human Development Survey 2005. Ann Arbor, Michigan: Inter-University Consortium for Political and Social Research.
Dyson PA, Anthony D, Fenton B, Matthews DR, Stevens DE, Community Interventions for Health Collaboration. High rates of child hypertension associated with obesity: a community survey in China, India and Mexico. Paediatr Int Child Health. 2014; 34(1): 43–9.
Euromonitor International. Euromonitor Passport - Cigarette Statistics 1999-2016. London, United Kingdom: Euromonitor International.
Euromonitor International. Euromonitor Passport - Fresh Foods Market Statistics 2011-2016. London, United Kingdom: Euromonitor International.
Euromonitor International. Euromonitor Passport - Nuts Market Statistics 2001-2016. London, United Kingdom: Euromonitor International.
Euromonitor International. Euromonitor Passport - Processed Meat and Seafood Market Statistics 2002-2016. London, United Kingdom: Euromonitor International.
Euromonitor International. Euromonitor Passport - Vegetables Market Statistics 2001-2016. London, United Kingdom: Euromonitor International.
Fall CHD, Sachdev HS, Osmond C, Lakshmy R, Biswas SD, Prabhakaran D, Tandon N, Ramji S, Reddy KS, Barker DJP, Bhargava SK. Adult metabolic syndrome and impaired glucose tolerance are associated with different patterns of BMI gain during infancy: Data from the New Delhi Birth Cohort. Diabetes Care. 2008; 31(12): 2349-56.
FAO Supply Utilization Accounts 1961-2013. Personal Correspondence with Dr. Josef Schmidhuber, 2016. [Data shared for this analysis]
Food and Agriculture Organization of the United Nations (FAO). FAOSTAT Commodity Balances - Crops Primary Equivalent. Rome, Italy: FAO.
Food and Agriculture Organization of the United Nations (FAO). FAOSTAT Food Balance Sheets, October 2014. Rome, Italy: FAO.
Gajalakshmi V, Peto R, Kanimozhi VC, Whitlock G, Veeramani D. Cohort Profile: the Chennai prospective study of mortality among 500,000 adults in Tamil Nadu, South India. Int J Epidemiol. 2007; 36(6): 1190-5.
Geetha L, Deepa M, Anjana RM, Mohan V. Prevalence and clinical profile of metabolic obesity and phenotypic obesity in Asian Indians. J Diabetes Sci Technol. 2011; 5(2): 439–46.
Ghosh A, Bala SK. Anthropometric, body composition, and blood pressure measures among rural elderly adults of Asian Indian origin: the Santiniketan aging study. J Nutr Gerontol Geriatr. 2011; 30(3): 305–13.
Gill HK, Yadav SB, Ramesh V, Bhatia E. A prospective study of prevalence and association of peripheral neuropathy in Indian patients with newly diagnosed type 2 diabetes mellitus. J Postgrad Med. 2014; 60(3): 270–5.
Global Burden of Disease Health Financing Collaborator Network, Institute for Health Metrics and Evaluation (IHME). Global Development Assistance for Health, Government, Prepaid Private, and Out-of-Pocket Health Spending 1995-2014. Seattle, United States: IHME; 2017.
Goswami AK, Gupta SK, Kalaivani M, Nongkynrih B, Pandav CS. Burden of Hypertension and Diabetes among Urban Population Aged ≥ 60 years in South Delhi: A Community Based Study. J Clin Diagn Res. 2016; 10(3): LC01-05.
Gupta A, Gupta R, Sarna M, Rastogi S, Gupta VP, Kothari K. Prevalence of diabetes, impaired fasting glucose and insulin resistance syndrome in an urban Indian population. Diabetes Res Clin Pract. 2003; 61(1): 69-76.
Gupta R, Deedwania PC, Achari V, Asirvatham AJ, Bhansali A, Gupta A, Gupta B, Gupta S, Jali MV, Mahanta TG, Maheshwari A, Saboo B, Singh J. India Heart Watch Study 2005-2009. [Data shared for this analysis]
Gupta R, Gupta VP, Sarna M, Prakash H, Rastogi S, Gupta KD. Serial epidemiological surveys in an urban Indian population demonstrate increasing coronary risk factors among the lower socioeconomic strata. J Assoc Physicians India. 2003 May; 51:470–7.
Gupta R, Lodha S, Sharma KK, Sharma SK, Gupta S, Asirvatham AJ, et al. Evaluation of statin prescriptions in type 2 diabetes: India Heart Watch-2. BMJ Open Diabetes Res Care. 2016;4(1): e000275.
Gupta R, Misra A, Vikram NK, Kondal D, Gupta SS, Agrawal A, Pandey RM. Younger age of escalation of cardiovascular risk factors in Asian Indian subjects. BMC Cardiovasc Disord. 2009; 9:28.
Gupta R, Pandey RM, Misra A, Agrawal A, Misra P, Dey S, Rao S, Menon VU, Kamalamma N, Vasantha Devi KP, Revathi K, Vikram NK, Sharma V, Guptha S. High prevalence and low awareness, treatment and control of hypertension in Asian Indian women. J Hum Hypertens. 2012; 26(10): 585–93.
Gupta R, Sarna M, Thanvi J, Rastogi P, Kaul V, Gupta VP. High prevalence of multiple coronary risk factors in Punjabi Bhatia community: Jaipur Heart Watch-3. Indian Heart J. 2004; 56(6): 646-52.
Gupta R, Sharma AK, Gupta VP, Bhatnagar S, Rastogi S, Deedwania PC. Increased variance in blood pressure distribution and changing hypertension prevalence in an urban Indian population. J Hum Hypertens. 2003; 17(8): 535-40.
Gupta R, Sharma KK, Gupta BK, Gupta A, Saboo B, Maheshwari A, et al. Geographic epidemiology of cardiometabolic risk factors in middle class urban residents in India: cross-sectional study. J Glob Health. 2015 Jun;5(1):010411.
Gupta R. India - Jaipur Heart Watch Study Data on Blood Pressure, Cholesterol, BMI, and Fasting Blood Glucose 1993-2001. [Data shared for this analysis]

Healis-Sekhsaria Institute for Public Health, Madhya Pradesh Voluntary Health Association, Cancer Foundation of India, School of Preventive Oncology, University of Waterloo. International Tobacco Control Policy Evaluation Project: India Tobacco Control Survey 2010-2011. Navi Mumbai, India and Waterloo, Canada: Healis-Sekhsaria Institute for Public Health and University of Waterloo.

Huffman MD, Prabhakaran D, Osmond C, Fall CHD, Tandon N, Lakshmy R, Ramji S, Khalil A, Gera T, Prabhakaran P, Biswas SKD, Reddy KS, Bhargava SK, Sachdev HS, New Delhi Birth Cohort. Incidence of cardiovascular risk factors in an Indian urban cohort results from the New Delhi birth cohort. J Am Coll Cardiol. 2011; 57(17): 1765-74.

Indian Council of Medical Research (ICMR), Madras Diabetes Research Foundation. Indian Council of Medical Research India Diabetes Study (ICMR-INDIAB) - North East 2012-2015. [Data shared for this analysis]

Indian Council of Medical Research (ICMR), Madras Diabetes Research Foundation. Indian Council of Medical Research India Diabetes Study (ICMR-INDIAB) 2008-2010. [Data shared for this analysis]

Indian Council of Medical Research (ICMR), Madras Diabetes Research Foundation. Indian Council of Medical Research India Diabetes Study (ICMR-INDIAB) 2012-2013. [Data shared for this analysis]

Indian Council of Medical Research (ICMR). India Study on Causes of Death by Verbal Autopsy 2003. New Delhi, India: ICMR. [Data shared for this analysis]

Indian Council of Medical Research, World Health Organization. India STEPS Noncommunicable Disease Risk Factors Survey 2003-2005. [Data shared for this analysis]

Indian Society of Nephrology. Indian Society for Nephrology Chronic Kidney Disease Registry 2008-2013. [Data shared for this analysis]

Institute of Health Systems, World Health Organization (WHO). WHO Multi-Country Survey Study Report on Health and Health System Responsiveness, Andhra Pradesh 2000-2001.

International Institute for Population Sciences (IIPS), Ministry of Health and Family Welfare, Government of India, Harvard T. H. Chan School of Public Health, RAND Corporation, Monash University, University of California Los Angeles, Indian Academy of Geriatrics, National AIDS Research Institute, Columbia University. Longitudinal Aging Study in India, Pilot Data 2010. Mumbai, India: IIPS.

International Institute for Population Sciences (IIPS), Ministry of Health and Family Welfare, Government of India, Macro International. India National Family Health Survey Data (NFHS-1) 1992-1993. Mumbai, India: IIPS.

International Institute for Population Sciences (IIPS), Ministry of Health and Family Welfare, Government of India, Macro International. India National Family Health Survey (NFHS-1) 1992-1993: National Report. Mumbai, India: IIPS.

International Institute for Population Sciences (IIPS), Ministry of Health and Family Welfare, Government of India, ORC Macro. India National Family Health Survey Data (NFHS-2) 1998-1999. Mumbai, India: IIPS.

International Institute for Population Sciences (IIPS), Ministry of Health and Family Welfare, Government of India, ORC Macro. India National Family Health Survey (NFHS-2) 1998-1999: National and State Reports. Mumbai, India: IIPS.

International Institute for Population Sciences (IIPS), Ministry of Health and Family Welfare, Government of India, Macro International. India National Family Health Survey Data (NFHS-3) 2005-2006. Mumbai, India: IIPS.

International Institute for Population Sciences (IIPS), Ministry of Health and Family Welfare, Government of India, Macro International. India National Family Health Survey (NFHS-3) 2005-2006: National and State Reports. Mumbai, India: IIPS.

International Institute for Population Sciences (IIPS), Ministry of Health and Family Welfare, Government of India, ICF. India National Family Health Survey (NFHS-4) 2015-2016: National and State-level Factsheets. Mumbai, India: IIPS.

International Institute for Population Sciences (IIPS), Ministry of Health and Family Welfare, Government of India. India District Level Household Survey Data (DLHS-1) 1998-1999. Mumbai, India: IIPS.

International Institute for Population Sciences (IIPS), Ministry of Health and Family Welfare, Government of India. India District Level Household Survey (DLHS-1) 1998-1999: National Report. Mumbai, India: IIPS.

International Institute for Population Sciences (IIPS), Ministry of Health and Family Welfare, Government of India. India District Level Household Survey Data (DLHS-2) 2002-2004. Mumbai, India: IIPS.

International Institute for Population Sciences (IIPS), Ministry of Health and Family Welfare, Government of India. India District Level Household Survey (DLHS-2) 2002-2004: National and State Reports. Mumbai, India: IIPS.

International Institute for Population Sciences (IIPS), Ministry of Health and Family Welfare, Government of India. India District Level Household and Facility Survey Data (DLHS-3) 2007-2008. Mumbai, India: IIPS.

International Institute for Population Sciences (IIPS), Ministry of Health and Family Welfare, Government of India. India District Level Household and Facility Survey (DLHS-3) 2007-2008: National and State Reports. Mumbai, India: IIPS.

International Institute for Population Sciences, Ministry of Health and Family Welfare, Government of India. India District Level Household and Facility Survey Data (DLHS-4) 2012-2013. Mumbai, India: IIPS.

International Institute for Population Sciences (IIPS), Ministry of Health and Family Welfare, Government of India. India District Level Household and Facility Survey (DLHS-4) 2012-2013: State Reports. Mumbai, India: IIPS.

International Institute for Population Sciences, Population Council, Ministry of Health and Family Welfare, Government of India. Youth in India: Situation and Needs Study 2006–2007. Mumbai, India: IIPS.

International Institute for Population Sciences, World Health Organization (WHO). India WHO Study on Global Ageing and Adult Health 2007-2008.

International Institute for Population Sciences, World Health Organization (WHO). India WHO Study on Global Ageing and Adult Health 2014-2015.

International Institute for Population Sciences, World Health Organization. India World Health Survey 2003.

Isharwal S, Arya S, Misra A, Wasir JS, Pandey RM, Rastogi K, Vikram NK, Luthra K, Sharma R. Dietary nutrients and insulin resistance in urban Asian Indian adolescents and young adults. Ann Nutr Metab. 2008; 52(2): 145–51.

Jayaprakash P, Bhansali S, Bhansali A, Dutta P, Anantharaman R. Magnitude of foot problems in diabetes in the developing world: a study of 1044 patients. Diabet Med. 2009; 26(9): 939-42.

Kamble P, Deshmukh PR, Garg N. Metabolic syndrome in adult population of rural Wardha, central India. Indian J Med Res. 2010; 132: 701–5.

Kaur S, Sachdev HPS, Dwivedi SN, Lakshmy R, Kapil U. Prevalence of overweight and obesity amongst school children in Delhi, India. Asia Pac J Clin Nutr. 2008; 17(4): 592-6.
KEM Hospital Research Center. India - Pimpale Cardiovascular Risk Factors Study Blood Pressure, Cholesterol, BMI, Blood Glucose, and Diabetes Incidence Measurements 1994-1999. [Data shared for this analysis]
Krishnan MN, Zachariah G, Venugopal K, Mohanan PP, Harikrishnan S, Sanjay G, Jeyaseelan L, Thankappan KR. Prevalence of coronary artery disease and its risk factors in Kerala, South India: a community-based cross-sectional study. BMC Cardiovasc Disord. 2013; 16: 12.
Kumar P, Krishna P, Reddy SC, Gurappa M, Aravind SR, Munichoodappa C. Incidence of type 1 diabetes mellitus and associated complications among children and young adults: results from Karnataka Diabetes Registry 1995-2008. J Indian Med Assoc. 2008; 106(11): 708–11.
Kusuma Y, Das P. Hypertension in Orissa, India: a cross-sectional study among some tribal, rural and urban populations. Public Health. 2008; 122(10): 1120-3.
Kusuma Y, Gupta S, Pandav C. Migration and hypertension: a cross-sectional study among neo-migrants and settled-migrants in Delhi, India. Asia Pac J Public Health. 2009; 21(4): 497-507.
Kusuma YS, Babu BV, Naidu JM. Blood pressure levels among cross-cultural populations of Visakhapatnam district, Andhra Pradesh, India. Ann Hum Biol. 2002; 29(5): 502-12.
Kutty VR, Soman CR, Joseph A, Pisharody R, Vijayakumar K. Type 2 diabetes in southern Kerala: variation in prevalence among geographic divisions within a region. Natl Med J India. 2000; 13(6): 287-92.
Madras Diabetes Research Foundation & M. V. Diabetes Specialities Centre. India - Chennai Urban Population Study Blood Glucose, Cholesterol, BMI, and Diabetes Incidence Measurements, 1996-2006. [Data shared for this analysis]
Madras Diabetes Research Foundation & M. V. Diabetes Specialities Centre. India - Chennai Urban Rural Epidemiology Study Blood Glucose, Cholesterol, BMI, and Diabetes Incidence Measurements, 2001-2013. [Data shared for this analysis]
Mahanta TG, Joshi R, Mahanta BN, Xavier D. Prevalence of modifiable cardiovascular risk factors among tea garden and general population in Dibrugarh, Assam, India. J Epidemiol Glob Health. 2013; 3(3): 147–56.
Malhotra P, Kumari S, Kumar R, Jain S, Sharma BK. Prevalence and determinants of hypertension in an un-industrialised rural population of North India. J Hum Hypertens. 1999; 13(7): 467-72.
Midha T, Idris MZ, Saran RK, Srivastav AK, Singh SK. Prevalence and determinants of hypertension in the urban and rural population of a north Indian district. East Afr J Public Health. 2009; 6(3): 268–73.
Ministry of Health and Family Welfare, Government of India, International Institute for Population Sciences, Centers for Disease Control and Prevention (CDC), Johns Hopkins Bloomberg School of Public Health, Research Triangle Institute, Inc., World Health Organization. India Global Adult Tobacco Survey 2009-2010. Atlanta, United States: CDC.
Ministry of Health and Family Welfare, Government of India, United Nations Children's Fund. India Coverage Evaluation Survey 2005.
Ministry of Health and Family Welfare, Government of India, United Nations Children's Fund. India Coverage Evaluation Survey 2007.
Ministry of Health and Family Welfare, Government of India, United Nations Children's Fund. India Coverage Evaluation Survey Data 2009-2010. [Data shared for this analysis]
Ministry of Health and Family Welfare, Government of India, World Health Organization - Regional Office for South-East Asia and Country Office for India, Centers for Disease Control and Prevention. India-Tripura Global Youth Tobacco Survey 2000. New Delhi, India: Ministry of Health and Family Welfare.
Ministry of Health and Family Welfare, Government of India, World Health Organization - Regional Office for South-East Asia and Country Office for India, Centers for Disease Control and Prevention. India-West Bengal Global Youth Tobacco Survey 2000. New Delhi, India: Ministry of Health and Family Welfare.
Ministry of Health and Family Welfare, Government of India, World Health Organization - Regional Office for South-East Asia and Country Office for India, Centers for Disease Control and Prevention. India-Arunachal Pradesh Global Youth Tobacco Survey 2000. New Delhi, India: Ministry of Health and Family Welfare.
Ministry of Health and Family Welfare, Government of India, World Health Organization - Regional Office for South-East Asia and Country Office for India, Centers for Disease Control and Prevention. India-Assam Global Youth Tobacco Survey 2000. New Delhi, India: Ministry of Health and Family Welfare.
Ministry of Health and Family Welfare, Government of India, World Health Organization - Regional Office for South-East Asia and Country Office for India, Centers for Disease Control and Prevention. India-Bihar Global Youth Tobacco Survey 2000. New Delhi, India: Ministry of Health and Family Welfare.
Ministry of Health and Family Welfare, Government of India, World Health Organization - Regional Office for South-East Asia and Country Office for India, Centers for Disease Control and Prevention. India-Goa Global Youth Tobacco Survey 2000. New Delhi, India: Ministry of Health and Family Welfare.
Ministry of Health and Family Welfare, Government of India, World Health Organization - Regional Office for South-East Asia and Country Office for India, Centers for Disease Control and Prevention. India-Maharashtra Global Youth Tobacco Survey 2000. New Delhi, India: Ministry of Health and Family Welfare.
Ministry of Health and Family Welfare, Government of India, World Health Organization - Regional Office for South-East Asia and Country Office for India, Centers for Disease Control and Prevention. India-Manipur Global Youth Tobacco Survey 2000. New Delhi, India: Ministry of Health and Family Welfare.
Ministry of Health and Family Welfare, Government of India, World Health Organization - Regional Office for South-East Asia and Country Office for India, Centers for Disease Control and Prevention. India-Meghalaya Global Youth Tobacco Survey 2000. New Delhi, India: Ministry of Health and Family Welfare.
Ministry of Health and Family Welfare, Government of India, World Health Organization - Regional Office for South-East Asia and Country Office for India, Centers for Disease Control and Prevention. India-Mizoram Global Youth Tobacco Survey 2000. New Delhi, India: Ministry of Health and Family Welfare.
Ministry of Health and Family Welfare, Government of India, World Health Organization - Regional Office for South-East Asia and Country Office for India, Centers for Disease Control and Prevention. India-Nagaland Global Youth Tobacco Survey 2000. New Delhi, India: Ministry of Health and Family Welfare.

Ministry of Health and Family Welfare, Government of India, World Health Organization - Regional Office for South-East Asia and Country Office for India, Centers for Disease Control and Prevention. India-Sikkim Global Youth Tobacco Survey 2000. New Delhi, India: Ministry of Health and Family Welfare.
Ministry of Health and Family Welfare, Government of India, World Health Organization - Regional Office for South-East Asia and Country Office for India, Centers for Disease Control and Prevention. India-Tamil Nadu Global Youth Tobacco Survey 2000. New Delhi, India: Ministry of Health and Family Welfare.
Ministry of Health and Family Welfare, Government of India, World Health Organization - Regional Office for South-East Asia and Country Office for India, Centers for Disease Control and Prevention. India-Uttar Pradesh Global Youth Tobacco Survey 2002. New Delhi, India: Ministry of Health and Family Welfare.
Ministry of Health and Family Welfare, Government of India, World Health Organization - Regional Office for South-East Asia and Country Office for India, Centres for Disease Control and Prevention. India-Uttaranchal Global Youth Tobacco Survey 2002. New Delhi, India: Ministry of Health and Family Welfare.
Ministry of Health and Family Welfare, Government of India, World Health Organization - Regional Office for South-East Asia and Country Office for India, Centers for Disease Control and Prevention. India-Karnataka Global Youth Tobacco Survey 2003. New Delhi, India: Ministry of Health and Family Welfare.
Ministry of Health and Family Welfare, Government of India, World Health Organization - Regional Office for South-East Asia and Country Office for India, Centers for Disease Control and Prevention. India-Rajasthan Global Youth Tobacco Survey 2003. New Delhi, India: Ministry of Health and Family Welfare.
Ministry of Health and Family Welfare, Government of India, World Health Organization - Regional Office for South-East Asia and Country Office for India, Centers for Disease Control and Prevention. India-Gujarat Global Youth Tobacco Survey 2004. New Delhi, India: Ministry of Health and Family Welfare.
Ministry of Health and Family Welfare, Government of India, World Health Organization - Regional Office for South-East Asia and Country Office for India, Centers for Disease Control and Prevention. India-Gujarat with Ahmedabad Global Youth Tobacco Survey 2004. New Delhi, India: Ministry of Health and Family Welfare.
Ministry of Health and Family Welfare, Government of India, World Health Organization - Regional Office for South-East Asia and Country Office for India, Centers for Disease Control and Prevention. India-Haryana Global Youth Tobacco Survey 2004. New Delhi, India: Ministry of Health and Family Welfare.
Ministry of Health and Family Welfare, Government of India, World Health Organization - Regional Office for South-East Asia and Country Office for India, Centers for Disease Control and Prevention. India-Jammu and Kashmir Global Youth Tobacco Survey 2004. New Delhi, India: Ministry of Health and Family Welfare.
Ministry of Health and Family Welfare, Government of India, World Health Organization - Regional Office for South-East Asia and Country Office for India, Centers for Disease Control and Prevention. India-Madhya Pradesh Global Youth Tobacco Survey 2004. New Delhi, India: Ministry of Health and Family Welfare.
Ministry of Health and Family Welfare, Government of India, World Health Organization - Regional Office for South-East Asia and Country Office for India, Centers for Disease Control and Prevention. India-Punjab Global Youth Tobacco Survey 2004. New Delhi, India: Ministry of Health and Family Welfare.
Ministry of Health and Family Welfare, Government of India, World Health Organization - Regional Office for South-East Asia and Country Office for India, Centers for Disease Control and Prevention. India Global Youth Tobacco Survey 2006. New Delhi, India: Ministry of Health and Family Welfare.
Ministry of Health and Family Welfare, Government of India, World Health Organization - Regional Office for South-East Asia and Country Office for India, Centers for Disease Control and Prevention. India Global Youth Tobacco Survey 2009. New Delhi, India: Ministry of Health and Family Welfare.
Ministry of Statistics and Programme Implementation, Government of India. Consumer Expenditure Survey, National Sample Survey Round 38, January-December 1983. New Delhi, India: Ministry of Statistics and Programme Implementation.
Ministry of Statistics and Programme Implementation, Government of India. Employment and Unemployment Survey, National Sample Survey Round 38, January-December 1983. New Delhi, India: Ministry of Statistics and Programme Implementation.
Ministry of Statistics and Programme Implementation, Government of India. Survey on Participation in Education, National Sample Survey Round 42, July 1986- June 1987. New Delhi, India: Ministry of Statistics and Programme Implementation.
Ministry of Statistics and Programme Implementation, Government of India. Consumer Expenditure Survey, National Sample Survey Round 43, July 1987-June 1988. New Delhi, India: Ministry of Statistics and Programme Implementation.
Ministry of Statistics and Programme Implementation, Government of India. Employment and Unemployment Survey, National Sample Survey Round 43, July 1987- June 1988. New Delhi, India: Ministry of Statistics and Programme Implementation.
Ministry of Statistics and Programme Implementation, Government of India. Survey on Literacy and Culture, National Sample Survey Round 47, July - December 1991. New Delhi, India: Ministry of Statistics and Programme Implementation.
Ministry of Statistics and Programme Implementation, Government of India. Consumer Expenditure Survey, National Sample Survey Round 49, January-June 1993. New Delhi, India: Ministry of Statistics and Programme Implementation.
Ministry of Statistics and Programme Implementation, Government of India. Consumer Expenditure Survey, National Sample Survey Round 50, July 1993-June 1994. New Delhi, India: Ministry of Statistics and Programme Implementation.
Ministry of Statistics and Programme Implementation, Government of India. Employment and Unemployment Survey, National Sample Survey Round 50, July 1993- June 1994. New Delhi, India: Ministry of Statistics and Programme Implementation.
Ministry of Statistics and Programme Implementation, Government of India. Survey on Healthcare, National Sample Survey Round 52, July 1995-June 1996. New Delhi, India: Ministry of Statistics and Programme Implementation.
Ministry of Statistics and Programme Implementation, Government of India. Employment and Unemployment Survey, India National Sample Survey Round 55, July 1999-June 2000. New Delhi, India: Ministry of Statistics and Programme Implementation.
Ministry of Statistics and Programme Implementation, Government of India. Consumer Expenditure Survey, National Sample Survey Round 60, January-June 2004. New Delhi, India: Ministry of Statistics and Programme Implementation.
Ministry of Statistics and Programme Implementation, Government of India. Employment and Unemployment Survey, National Sample Survey Round 61, July 2004- June 2005. New Delhi, India: Ministry of Statistics and Programme Implementation.

Ministry of Statistics and Programme Implementation, Government of India. Consumer Expenditure Survey, National Sample Survey Round 64, July 2007- June 2008. New Delhi, India: Ministry of Statistics and Programme Implementation.
Ministry of Statistics and Programme Implementation, Government of India. Social Consumption Survey on Health and Education, National Sample Survey Round 71, January 2014 - June 2014. New Delhi, India: Ministry of Statistics and Programme Implementation.
Ministry of Statistics and Programme Implementation, Government of India. Household expenditure on services and durable goods survey, National Sample Survey Round 72, July 2014 - June 2015. New Delhi, India: Ministry of Statistics and Programme Implementation.
Ministry of Statistics and Programme Implementation, Government of India. India State Series: Gross State Domestic Product at Factor Cost by Industry of Origin Tables 1980-81 to 1993-94. New Delhi, India: Ministry of Statistics and Programme Implementation.
Ministry of Statistics and Programme Implementation, Government of India. India State Series: Gross State Domestic Product at Factor Cost by Industry of Origin Tables 1993-94 to 2004-05. New Delhi, India: Ministry of Statistics and Programme Implementation.
Ministry of Statistics and Programme Implementation, Government of India. India State Series: Gross State Domestic Product at Factor Cost by Industry of Origin Tables 2004-05 to 2013-14. New Delhi, India: Ministry of Statistics and Programme Implementation.
Mohan V, Deepa M, Anjana RM, Lanthorn H, Deepa R. Incidence of diabetes and pre-diabetes in a selected urban south Indian population (CUPS - 19). J Assoc Physicians India. 2008; 56: 152-7.
Mohan V, Deepa M, Farooq S, Datta M, Deepa R. Prevalence, awareness and control of hypertension in Chennai--The Chennai Urban Rural Epidemiology Study (CURES-52). J Assoc Physicians India. 2007; 55: 326-32.
Mohan V, Mathur P, Deepa R, Deepa M, Shukla DK, Menon GR, Anand K, Desai NG, Joshi PP, Mahanta J, Thankappan KR, Shah B. Urban rural differences in prevalence of self-reported diabetes in India--the WHO-ICMR Indian NCD risk factor surveillance. Diabetes Res Clin Pract. 2008; 80(1): 159-68.
Mohan V, Vijayaprabha R, Rema M. Vascular complications in long-term south Indian NIDDM of over 25 years' duration. Diabetes Res Clin Pract. 1996; 31(1-3): 133–40.
Muninarayana C, Balachandra G, Hiremath SG, Iyengar K, Anil NS. Prevalence and awareness regarding diabetes mellitus in rural Tamaka, Kolar. Int J Diabetes Dev Ctries. 2010; 30(1): 18-21.
National Institute of Medical Statistics, Indian Council of Medical Research (ICMR), Integrated Disease Surveillance Programme. Non-Communicable Disease Risk Factors Survey Data 2007-2008. New Delhi, India: ICMR. [Data shared for this analysis]
National Institute of Medical Statistics, Indian Council of Medical Research (ICMR), Integrated Disease Surveillance Programme. Non-Communicable Disease Risk Factors Survey Report 2007-2008. New Delhi, India: ICMR.
National Nutrition Monitoring Bureau, National Institute of Nutrition (NIN), Indian Council of Medical Research. India Rural First Repeat Survey of Diet and Nutritional Status Data 1988-1990. [Data shared for this analysis]
National Nutrition Monitoring Bureau, National Institute of Nutrition (NIN), Indian Council of Medical Research. India Rural First Repeat Survey of Diet and Nutritional Status Report 1988-1990. Hyderabad, India: NIN.
National Nutrition Monitoring Bureau, National Institute of Nutrition (NIN), Indian Council of Medical Research. India Survey of Diet and Nutritional Status Data 1990-1992. [Data shared for this analysis]
National Nutrition Monitoring Bureau, National Institute of Nutrition (NIN), Indian Council of Medical Research. India Survey of Diet and Nutritional Status Report 1990-1992. Hyderabad, India: NIN.
National Nutrition Monitoring Bureau, National Institute of Nutrition, Indian Council of Medical Research. India National Nutrition Monitoring Bureau Eight States Pooled Data 1991-1992. [Data shared for this analysis]
National Nutrition Monitoring Bureau, National Institute of Nutrition (NIN), Indian Council of Medical Research. India Urban Slums Survey of Diet and Nutritional Status Data 1993-1994. [Data shared for this analysis]
National Nutrition Monitoring Bureau, National Institute of Nutrition (NIN), Indian Council of Medical Research. India Urban Slums Survey of Diet and Nutritional Status Report 1993-1994. Hyderabad, India: NIN.
National Nutrition Monitoring Bureau, National Institute of Nutrition (NIN), Indian Council of Medical Research. India Rural Survey of Diet and Nutritional Status Data 1994-1995. [Data shared for this analysis]
National Nutrition Monitoring Bureau, National Institute of Nutrition (NIN), Indian Council of Medical Research. India Rural Survey of Diet and Nutritional Status Report 1994-1995. Hyderabad, India: NIN.
National Nutrition Monitoring Bureau, National Institute of Nutrition (NIN), Indian Council of Medical Research. India Rural Second Repeat Survey of Diet and Nutritional Status Data 1996-1997. [Data shared for this analysis]
National Nutrition Monitoring Bureau, National Institute of Nutrition (NIN), Indian Council of Medical Research. India Rural Second Repeat Survey of Diet and Nutritional Status Report 1996-1997. Hyderabad, India: NIN.
National Nutrition Monitoring Bureau, National Institute of Nutrition (NIN), Indian Council of Medical Research. India Tribal First Repeat Survey of Diet and Nutritional Status Data 1998-1999. [Data shared for this analysis]
National Nutrition Monitoring Bureau, National Institute of Nutrition (NIN), Indian Council of Medical Research. India Tribal First Repeat Survey of Diet and Nutritional Status Report 1998-1999. Hyderabad, India: NIN.
National Nutrition Monitoring Bureau, National Institute of Nutrition (NIN), Indian Council of Medical Research. India Rural Survey of Diet and Nutritional Status Data 2000-2001. [Data shared for this analysis]
National Nutrition Monitoring Bureau, National Institute of Nutrition (NIN), Indian Council of Medical Research. India Rural Survey of Diet and Nutritional Status Report 2000-2001. Hyderabad, India: NIN.
National Nutrition Monitoring Bureau, National Institute of Nutrition (NIN), Indian Council of Medical Research. India Rural Survey of Diet and Nutritional Status Data 2004-2006. [Data shared for this analysis]
National Nutrition Monitoring Bureau, National Institute of Nutrition (NIN), Indian Council of Medical Research. India Rural Survey of Diet and Nutritional Status Report 2004-2006. Hyderabad, India: NIN.
National Nutrition Monitoring Bureau, National Institute of Nutrition (NIN), Indian Council of Medical Research. India Tribal Second Repeat Survey of Diet and Nutritional Status Data 2007-2008. [Data shared for this analysis]
National Nutrition Monitoring Bureau, National Institute of Nutrition (NIN), Indian Council of Medical Research. India Tribal Second Repeat Survey of Diet and Nutritional Status Report 2007-2008. Hyderabad, India: NIN.

National Nutrition Monitoring Bureau, National Institute of Nutrition (NIN), Indian Council of Medical Research. India Rural Third Repeat Survey of Diet and Nutritional Status Data 2011-2012. [Data shared for this analysis]
National Nutrition Monitoring Bureau, National Institute of Nutrition (NIN), Indian Council of Medical Research. India Rural Third Repeat Survey of Diet and Nutritional Status Report 2011-2012. Hyderabad, India: NIN.
Nazir A, Papita R, Anbalagan VP, Anjana RM, Deepa M, Mohan V. Prevalence of diabetes in Asian Indians based on glycated hemoglobin and fasting and 2-H post-load (75-g) plasma glucose (CURES-120). Diabetes Technol Ther. 2012; 14(8): 665–8.
Norboo T, Stobdan T, Tsering N, Angchuk N, Tsering P, Ahmed I, Chorol T, Kumar Sharma V, Reddy P, Singh SB, Kimura Y, Sakamoto R, Fukutomi E, Ishikawa M, Suwa K, Kosaka Y, Nose M, Yamaguchi T, Tsukihara T, Matsubayashi K, Otsuka K, Okumiya K. Prevalence of hypertension at high altitude: cross-sectional survey in Ladakh, Northern India 2007-2011. BMJ Open. 2015; 5(4): e007026.
Office of the Registrar General & Census Commissioner, Centre for Global Health Research - University of Toronto. India SRS Special Fertility and Mortality Survey 1998. New Delhi, India: Office of the Registrar General & Census Commissioner; 2005.
Office of the Registrar General & Census Commissioner, Ministry of Home Affairs, Government of India. India Annual Health Survey Data 2010-2011. New Delhi, India: Office of the Registrar General & Census Commissioner.
Office of the Registrar General & Census Commissioner, Ministry of Home Affairs, Government of India. India Annual Health Survey Report 2010-2011. New Delhi, India: Office of the Registrar General & Census Commissioner.
Office of the Registrar General & Census Commissioner, Ministry of Home Affairs, Government of India. India Annual Health Survey Data 2010-2013. New Delhi, India: Office of the Registrar General & Census Commissioner.
Office of the Registrar General & Census Commissioner, Ministry of Home Affairs, Government of India. India Annual Health Survey Report 2010-2013. New Delhi, India: Office of the Registrar General & Census Commissioner.
Office of the Registrar General & Census Commissioner, Ministry of Home Affairs, Government of India. India Annual Health Survey Data 2011-2012. New Delhi, India: Office of the Registrar General & Census Commissioner.
Office of the Registrar General & Census Commissioner, Ministry of Home Affairs, Government of India. India Annual Health Survey Report 2011-2012. New Delhi, India: Office of the Registrar General & Census Commissioner.
Office of the Registrar General & Census Commissioner, Ministry of Home Affairs, Government of India. India Annual Health Survey Data 2012-2013. New Delhi, India: Office of the Registrar General & Census Commissioner.
Office of the Registrar General & Census Commissioner, Ministry of Home Affairs, Government of India. India Annual Health Survey Report 2012-2013. New Delhi, India: Office of the Registrar General & Census Commissioner.
Office of the Registrar General & Census Commissioner, Ministry of Home Affairs, Government of India. India Census 1981. New Delhi, India: Office of the Registrar General & Census Commissioner.
Office of the Registrar General & Census Commissioner, Ministry of Home Affairs, Government of India. India Population and Housing Census 2001. New Delhi, India: Office of the Registrar General & Census Commissioner.
Office of the Registrar General & Census Commissioner, Ministry of Home Affairs, Government of India. India Sample Registration System Baseline Survey 2014. New Delhi, India: Office of the Registrar General & Census Commissioner.
Office of the Registrar General & Census Commissioner, Ministry of Home Affairs, Government of India. India SRS Statistical Report 1999. New Delhi, India: Office of the Registrar General & Census Commissioner.
Office of the Registrar General & Census Commissioner, Ministry of Home Affairs, Government of India. India SRS Statistical Report 2000. New Delhi, India: Office of the Registrar General & Census Commissioner.
Office of the Registrar General & Census Commissioner, Ministry of Home Affairs, Government of India. India SRS Statistical Report 2001. New Delhi, India: Office of the Registrar General & Census Commissioner; 2005.
Office of the Registrar General & Census Commissioner, Ministry of Home Affairs, Government of India. India SRS Statistical Report 2002. New Delhi, India: Office of the Registrar General & Census Commissioner; 2005.
Office of the Registrar General & Census Commissioner, Ministry of Home Affairs, Government of India. India SRS Statistical Report 2003. New Delhi, India: Office of the Registrar General and Census Commissioner; 2005.
Office of the Registrar General & Census Commissioner, Ministry of Home Affairs, Government of India. India SRS Statistical Report 2004. New Delhi, India: Office of the Registrar General and Census Commissioner; 2006.
Office of the Registrar General & Census Commissioner, Ministry of Home Affairs, Government of India. India SRS Statistical Report 2005. New Delhi, India: Office of the Registrar General and Census Commissioner; 2006.
Office of the Registrar General & Census Commissioner, Ministry of Home Affairs, Government of India. India SRS Statistical Report 2006. New Delhi, India: Office of the Registrar General and Census Commissioner; 2008.
Office of the Registrar General & Census Commissioner, Ministry of Home Affairs, Government of India. India SRS Statistical Report 2007. New Delhi, India: Office of the Registrar General and Census Commissioner; 2008.
Office of the Registrar General & Census Commissioner, Ministry of Home Affairs, Government of India. India SRS Statistical Report 2008. New Delhi, India: Office of the Registrar General and Census Commissioner; 2010.
Office of the Registrar General & Census Commissioner, Ministry of Home Affairs, Government of India. India SRS Statistical Report 2009. New Delhi, India: Office of the Registrar General and Census Commissioner.
Office of the Registrar General & Census Commissioner, Ministry of Home Affairs, Government of India. India SRS Statistical Report 2010. New Delhi, India: Office of the Registrar General and Census Commissioner; 2012.
Office of the Registrar General & Census Commissioner, Ministry of Home Affairs, Government of India. India SRS Statistical Report 2011. New Delhi, India: Office of the Registrar General and Census Commissioner; 2013.
Office of the Registrar General & Census Commissioner, Ministry of Home Affairs, Government of India. India SRS Statistical Report 2012. New Delhi, India: Office of the Registrar General and Census Commissioner; 2013.
Office of the Registrar General & Census Commissioner, Ministry of Home Affairs, Government of India. India SRS Statistical Report 2013. New Delhi, India: Office of the Registrar General and Census Commissioner; 2014.
Office of the Registrar General & Census Commissioner, Ministry of Home Affairs, Government of India. India SRS Statistical Report 2014. New Delhi, India: Office of the Registrar General and Census Commissioner.

Office of the Registrar General & Census Commissioner, Ministry of Home Affairs, Government of India. India SRS Verbal Autopsy 2004-2006. New Delhi, India: Office of the Registrar General and Census Commissioner. [Data shared for this analysis]
Office of the Registrar General & Census Commissioner, Ministry of Home Affairs, Government of India. India SRS Verbal Autopsy Report 2004-2006. New Delhi, India: Office of the Registrar General and Census Commissioner.
Office of the Registrar General & Census Commissioner, Ministry of Home Affairs, Government of India. India SRS Verbal Autopsy 2007-2009. New Delhi, India: Office of the Registrar General and Census Commissioner. [Data shared for this analysis]
Office of the Registrar General & Census Commissioner, Ministry of Home Affairs, Government of India. India SRS Verbal Autopsy Report 2007-2009. New Delhi, India: Office of the Registrar General and Census Commissioner.
Office of the Registrar General & Census Commissioner, Ministry of Home Affairs, Government of India. India SRS Verbal Autopsy 2010-2013. New Delhi, India: Office of the Registrar General and Census Commissioner. [Data shared for this analysis]
Office of the Registrar General & Census Commissioner, Ministry of Home Affairs, Government of India. India SRS Verbal Autopsy Report 2010-2013. New Delhi, India: Office of the Registrar General and Census Commissioner.
Office of the Registrar General and Census Commissioner, Ministry of Health and Family Welfare, Government of India, National Institute of Health and Family Welfare, Nutrition Foundation of India, National Institute of Nutrition, Indian Council of Medical Research. India Clinical, Anthropometric and Bio-chemical (CAB) Survey Data 2014 [Biomarker Component of Annual Health Survey]. New Delhi, India: Office of the Registrar General and Census Commissioner.
Oommen AM, Abraham VJ, George K, Jose VJ. Prevalence of coronary heart disease in rural and urban Vellore: A repeat cross-sectional survey. Indian Heart J. 2016; 68(4): 473-9.
Patandin S, Bots ML, Abel R, Valkenburg HA. Impaired glucose tolerance and diabetes mellitus in a rural population in south India. Diabetes Res Clin Pract. 1994; 24(1): 47-53.
Pemminati S, Prabha Adhikari MR, Pathak R, Pai MRSM. Prevalence of metabolic syndrome (METS) using IDF 2005 guidelines in a semi urban south Indian (Boloor Diabetes Study) population of Mangalore. J Assoc Physicians India. 2010; 58: 674–7.
Pradeepa R, Rema M, Vignesh J, Deepa M, Deepa R, Mohan V. Prevalence and risk factors for diabetic neuropathy in an urban south Indian population: the Chennai Urban Rural Epidemiology Study (CURES-55). Diabet Med. 2008; 25(4): 407–12.
Prasad DS, Kabir Z, Dash AK, Das BC. Prevalence and risk factors for diabetes and impaired glucose tolerance in Asian Indians: a community survey from urban eastern India. Diabetes Metab Syndr. 2012; 6(2): 96-101.
Public Health Foundation of India. India Cause of Death Estimation Study in Bihar 2011-2014. New Delhi, India: Public Health Foundation of India. [Data shared for this analysis]
Radhakrishnan S, Balamurugan S. Prevalence of diabetes and hypertension among geriatric population in a rural community of Tamilnadu. Indian J Med Sci. 2013; 67(5-6): 130-8.
Rajput R, Rajput M, Singh J, Bairwa M. Prevalence of diabetes mellitus among the adult population in rural blocks of Haryana, India: a community-based study. Metab Syndr Relat Disord. 2012; 10(6): 443-6.
Ramachandran A, Mary S, Yamuna A, Murugesan N, Snehalatha C. High prevalence of diabetes and cardiovascular risk factors associated with urbanization in India. Diabetes Care. 2008; 31(5): 893-8.
Ramachandran A, Snehalatha C, Abdul Khader OM, Joseph TA, Viswanathan M. Prevalence of childhood diabetes in an urban population in south India. Diabetes Res Clin Pract. 1992; 17(3): 227-31.
Ramachandran A, Snehalatha C, Kapur A, Vijay V, Mohan V, Das AK, Rao PV, Yajnik CS, Prasanna Kumar KM, Nair JD. High prevalence of diabetes and impaired glucose tolerance in India: National Urban Diabetes Survey. Diabetologia. 2001; 44(9): 1094-101.
Ramachandran A, Snehalatha C, Latha E, Manoharan M, Vijay V. Impacts of urbanisation on the lifestyle and on the prevalence of diabetes in native Asian Indian population. Diabetes Res Clin Pract. 1999; 44(3): 207-13.
Ramachandran A, Snehalatha C, Satyavani K, Latha E, Sasikala R, Vijay V. Prevalence of vascular complications and their risk factors in type 2 diabetes. J Assoc Physicians India. 1999; 47(12): 1152–6.
Raman Kutty V, Joseph A, Soman CR. High prevalence of type 2 diabetes in an urban settlement in Kerala, India. Ethn Health. 1999; 4(4): 231-9.
Rani PK, Raman R, Gella L, Kulothungan V, Sharma T. Prevalence of Visual Impairment and Associated Risk Factors in Subjects with Type II Diabetes Mellitus: Sankara Nethralaya Diabetic Retinopathy Epidemiology and Molecular Genetics Study (SN-DREAMS, Report 16). Middle East Afr J Ophthalmol. 2012; 19(1): 129-34.
Rani PK, Raman R, Rachapalli SR, Pal SS, Kulothungan V, Sharma T. Prevalence and risk factors for severity of diabetic neuropathy in type 2 diabetes mellitus. Indian J Med Sci. 2010; 64(2): 51–7.
Rao CR, Kamath VG, Shetty A, Kamath A. A study on the prevalence of type 2 diabetes in coastal Karnataka. Int J Diabetes Dev Ctries. 2010; 30(2): 80-5.
Rapid Assessment of Avoidable Blindness (RAAB) Repository, Health Information Services. India Rapid Assessment of Avoidable Blindness Repository 1995-2016. Available from: http://raabdata.info/repository/
Ravikumar P, Bhansali A, Ravikiran M, Bhansali S, Walia R, Shanmugasundar G, Thakur JS, Kumar Bhadada S, Dutta P. Prevalence and risk factors of diabetes in a community-based study in North India: the Chandigarh Urban Diabetes Study (CUDS). Diabetes Metab. 2011; 37(3): 216-21.
Reddy KK, Rao AP, Reddy TP. Socioeconomic status and the prevalence of coronary heart disease risk factors. Asia Pac J Clin Nutr. 2002; 11(2): 98-103.
Ritchie GE, Kengne AP, Joshi R, Chow C, Neal B, Patel A, Zoungas S. Comparison of near-patient capillary glucose measurement and a risk assessment questionnaire in screening for type 2 diabetes in a high-risk population in rural India. Diabetes Care. 2011; 34(1): 44–9.
Sachdev HPS, Osmond C, Fall CHD, Lakshmy R, Ramji S, Dey Biswas SK, Prabhakaran D, Tandon N, Reddy KS, Barker DJP, Bhargava SK. Predicting adult metabolic syndrome from childhood body mass index: follow-up of the New Delhi birth cohort. Arch Dis Child. 2009; 94(10): 768-74.
Sadikot SM, Nigam A, Das S, Bajaj S, Zargar AH, Prasannakumar KM, Sosale A, Munichoodappa C, Seshiah V, Singh SK, Jamal A, Sai K, Sadasivrao Y, Murthy SS, Hazra DK, Jain S, Mukherjee S, Bandyopadhay S, Sinha NK, Mishra R, Dora M, Jena B, Patra P, Goenka K. The burden of diabetes and impaired glucose tolerance in India using the WHO 1999 criteria: prevalence of diabetes in India study (PODIS). Diabetes Res Clin Pract. 2004; 66(3): 301-7.

Samuel P, Antonisamy B, Raghupathy P, Richard J, Fall CHD. Socio-economic status and cardiovascular risk factors in rural and urban areas of Vellore, Tamilnadu, South India. Int J Epidemiol. 2012; 41(5): 1315-27.
Sangath, Columbia University, Alcohol Research Group, Public Health Institute, University of California San Francisco, London School of Hygiene and Tropical Medicine. Soryacher Asar Ani Hacher Amcho Shodh (SAAHAS) Goa Alcohol Use Study 2004-2008. Goa, India: Sangath.
Sauvaget C, Ramadas K, Thomas G, Thara S, Sankaranarayanan R. Prognosis criteria of casual systolic and diastolic blood pressure values in a prospective study in India. J Epidemiol Community Health. 2010; 64(4): 366–72.
Shah A, Afzal M. Prevalence of diabetes and hypertension and association with various risk factors among different Muslim populations of Manipur, India. J Diabetes Metab Disord. 2013; 12(1): 52.
Shah C, Sheth NR, Solanki B, Shah N. To assess the prevalence of impaired glucose tolerance and impaired fasting glucose in Western Indian population. J Assoc Physicians India. 2013; 61(3): 179–84.
Shrivastava SR, Ghorpade AG. High prevalence of type 2 diabetes mellitus and its risk factors among the rural population of Pondicherry, South India. J Res Health Sci. 2014; 14(4): 258-63.
Shukla V, Karoli R, Chandra A. A study of newly diagnosed type 2 diabetes mellitus patients from rural areas. J Assoc Physicians India. 2014; 62(8): 682–4.
Singh AK, Mani K, Krishnan A, Aggarwal P, Gupta SK. Prevalence, awareness, treatment and control of diabetes among elderly persons in an urban slum of Delhi. Indian J Community Med. 2012; 37(4): 236-9.
Singh RB, Bajaj S, Niaz MA, Rastogi SS, Moshiri M. Prevalence of type 2 diabetes mellitus and risk of hypertension and coronary artery disease in rural and urban population with low rates of obesity. Int J Cardiol. 1998; 66(1): 65-72.
Singh RB, Fedacko J, Pella D, Macejova Z, Ghosh S, de Amit K, Begom R, Tumbis ZA, Haque M, Vajpeyee SK, de Meester F, Sergey C, Agarwalo R, Muthusamy VV, Five City Study Group, Gupta AK. Prevalence and risk factors for prehypertension and hypertension in five Indian cities. Acta Cardiol. 2011; 66(1): 29-37.
Sinharay K, Paul UK, Bhattacharyya AK, Pal SK. Prevalence of diabetic foot ulcers in newly diagnosed diabetes mellitus patients. J Indian Med Assoc. 2012; 110(9): 608-11.
Sosale A, Prasanna Kumar KM, Sadikot SM, Nigam A, Bajaj S, Zargar AH, Singh SK. Chronic complications in newly diagnosed patients with Type 2 diabetes mellitus in India. Indian J Endocrinol Metab. 2014; 18(3): 355–60.
Subramanian SV, Smith GD. Patterns, distribution, and determinants of under- and overnutrition: a population-based study of women in India. Am J Clin Nutr. 2006; 84(3): 633-40.
The INTERSALT Co-operative Research Group. Appendix tables. Centre-specific results by age and sex. J Hum Hypertens 1989; 3(5):331-407.
Tobacco Sales Data, Personal Correspondence with Christopher Tan, 1997-2012. [Data shared for this analysis]
Tyrovolas S, Koyanagi A, Garin N, Olaya B, Ayuso-Mateos JL, Miret M, Chatterji S, Tobiasz-Adamczyk B, Koskinen S, Leonardi M, Haro JM. Determinants of the components of arterial pressure among older adults--the role of anthropometric and clinical factors: a multi-continent study. Atherosclerosis. 2015; 238(2): 240–9.
U.S. Department of Agriculture (USDA). USDA Global Tobacco Database 1960-2005. Washington DC, United States: USDA.
Vaz NC, Ferreira AM, Kulkarni MS, Vaz FS. Prevalence of diabetes mellitus in a rural population of Goa, India. Natl Med J India. 2011; 24(1): 16-8.
Vijayakumar G, Arun R, Kutty VR. High prevalence of type 2 diabetes mellitus and other metabolic disorders in rural Central Kerala. J Assoc Physicians India. 2009; 57: 563-7.
Viswanathan V, Kumpatla S. Pattern and causes of amputation in diabetic patients--a multicentric study from India. J Assoc Physicians India. 2011; 59:148–51.
Viswanathan V, Thomas N, Tandon N, Asirvatham A, Rajasekar S, Ramachandran A, Senthilvasan K, Murugan VS, Muthulakshmi. Profile of diabetic foot complications and its associated complications – a multicentric study from India. J Assoc Physicians India. 2005; 53: 933-6.
Walia R, Bhansali A, Ravikiran M, Ravikumar P, Bhadada SK, Shanmugasundar G, Dutta P, Sachdeva N. High prevalence of cardiovascular risk factors in Asian Indians: a community survey - Chandigarh Urban Diabetes Study (CUDS). Indian J Med Res. 2014; 139(2): 252–9.
World Health Organization. Study on Global Ageing and Adult Health (SAGE) Pilot Study 2005 Data from the Data Archive of Social Research on Aging. Los Altos, United States: Sociometrics Corporation. Available from: http://home.socio.com/age2728.php
Zaman FA, Borang A. Prevalence of diabetes mellitus amongst rural hilly population of North Eastern India and its relationship with associated risk factors and related co-morbidities. J Nat Sci Biol Med. 2014; 5(2): 383–8.
Zaman FA, Pal R, Zaman GS, Swati IA, Kayyum A. Glucose indices, frank and undetected diabetes in relation to hypertension and anthropometry in a South Indian rural population. Indian J Public Health. 2011; 55(1): 34–7.
Zargar AH, Khan AK, Masoodi SR, Laway BA, Wani AI, Bashir MI, Dar FA. Prevalence of type 2 diabetes mellitus and impaired glucose tolerance in the Kashmir Valley of the Indian subcontinent. Diabetes Res Clin Pract. 2000; 47(2): 135-46.

3. Number of persons with diabetes in the states of India, 2016

States of India (population in 2016)	Number of persons with diabetes (millions)	95% uncertainty interval
**India (1,316 million)**	**64.99**	**58.68 to 71.12**
**Low ETL (626 million)**	24.75	22.16 to 27.43
Bihar	3.41	3.02 to 3.81
Jharkhand	1.27	1.12 to 1.44
Uttar Pradesh	8.36	7.45 to 9.38
Rajasthan	2.58	2.3 to 2.87
Meghalaya	0.09	0.08 to 0.10
Assam	1.45	1.30 to 1.60
Chhattisgarh	1.27	1.12 to 1.42
Madhya Pradesh	4.26	3.88 to 4.66
Odisha	2.07	1.84 to 2.31
**Lower-middle ETL (92 million)**	4.18	3.80 to 4.58
Arunachal Pradesh	0.05	0.05 to 0.06
Mizoram	0.04	0.04 to 0.05
Nagaland	0.08	0.07 to 0.09
Uttarakhand	0.52	0.47 to 0.58
Gujarat	3.05	2.77 to 3.33
Tripura	0.27	0.25 to 0.30
Sikkim	0.03	0.02 to 0.03
Manipur	0.14	0.12 to 0.16
**Higher-middle ETL (446 million)**	23.09	20.81 to 25.34
Haryana	1.35	1.21 to 1.50
Delhi	1.48	1.36 to 1.59
Telangana	1.93	1.74 to 2.13
Andhra Pradesh	2.73	2.46 to 3.02
Jammu and Kashmir	0.49	0.44 to 0.55
Karnataka	4.14	3.79 to 4.52
West Bengal	4.39	3.89 to 4.92
Maharashtra	6.29	5.63 to 6.97
Union territories other than Delhi	0.29	0.26 to 0.31
**High ETL (152 million)**	12.97	11.96 to 13.97
Himachal Pradesh	0.30	0.26 to 0.33
Punjab	2.02	1.84 to 2.2
Tamil Nadu	7.17	6.63 to 7.69
Goa	0.10	0.09 to 0.12
Kerala	3.37	3.07 to 3.67

ETL is epidemiological transition level.

4. Prevalence of diabetes in adults 20 years of age or more in the states of India, 1990 and 2016

Crude prevalence (95% uncertainty interval)				Age-standardised percent change (95% uncertainty interval)	
**States of India***	**1990**	**States of India***	**2016**	**States of India***	**1990 to 2016**
Kerala	9.7 (8.9 to 10.4)	Tamil Nadu	13.1 (12.1 to 14.0)	Nagaland	55.1 (47.1 to 64.3)
Tamil Nadu	8.3 (7.6 to 9.2)	Kerala	12.3 (11.2 to 13.4)	Sikkim	52.5 (44.6 to 60.4)
Delhi	8.2 (7.5 to 9.0)	Delhi	10.8 (10.0 to 11.6)	Chhattisgarh	51.6 (44.1 to 60.7)
Union territories other than Delhi	7.5 (6.7 to 8.2)	Punjab	9.6 (8.8 to 10.5)	Goa	50.2 (43.4 to 57.4)
Punjab	7.2 (6.5 to 7.9)	Goa	9.2 (8.1 to 10.3)	Jammu and Kashmir	49.7 (43.1 to 58.8)
Karnataka	7.0 (6.3 to 7.7)	Karnataka	9.0 (8.3 to 9.9)	Uttarakhand	48.4 (40.3 to 56.3)
Tripura	6.1 (5.5 to 6.7)	Madhya Pradesh	8.7 (7.9 to 9.5)	Uttar Pradesh	45.4 (38.0 to 53.8)
Madhya Pradesh	6.0 (5.5 to 6.6)	Tripura	8.6 (7.8 to 9.4)	Odisha	45.3 (36.7 to 52.9)
Maharashtra	5.9 (5.3 to 6.5)	Union territories other than Delhi	8.5 (7.7 to 9.4)	Arunachal Pradesh	43.4 (36.0 to 51.4)
Telangana	5.7 (5.1 to 6.3)	Uttarakhand	7.7 (6.8 to 8.5)	Himachal Pradesh	43.1 (35.6 to 50.8)
Manipur	5.4 (4.8 to 6.2)	Andhra Pradesh	7.6 (6.8 to 8.4)	Haryana	41.9 (34.9 to 49.6)
Andhra Pradesh	5.4 (4.8 to 6.0)	Telangana	7.5 (6.7 to 8.3)	Tamil Nadu	41.3 (33.7 to 49.9)
Assam	5.1 (4.5 to 5.7)	Maharashtra	7.4 (6.6 to 8.2)	West Bengal	39.8 (33.8 to 46.3)
Jharkhand	5.1 (4.5 to 5.8)	Haryana	7.2 (6.4 to 8.0)	Madhya Pradesh	36.2 (31.0 to 41.0)
Goa	5.1 (4.5 to 5.7)	Chhattisgarh	7.2 (6.4 to 8.1)	Tripura	34.6 (28.6 to 40.3)
Uttarakhand	4.9 (4.3 to 5.5)	Gujarat	6.8 (6.2 to 7.5)	Gujarat	31.6 (24.6 to 39.4)
Haryana	4.8 (4.3 to 5.4)	Odisha	6.8 (6.1 to 7.6)	Rajasthan	31.5 (25.3 to 37.4)
Gujarat	4.8 (4.2 to 5.4)	Uttar Pradesh	6.7 (5.9 to 7.5)	Meghalaya	29.2 (22.2 to 35.3)
Chhattisgarh	4.6 (4.0 to 5.1)	Manipur	6.6 (5.9 to 7.5)	Andhra Pradesh	28.5 (22.6 to 34.8)
Uttar Pradesh	4.5 (3.9 to 5.0)	Sikkim	6.6 (5.8 to 7.4)	Punjab	23.3 (17.8 to 29.4)
Bihar	4.3 (3.8 to 4.9)	Assam	6.5 (5.9 to 7.2)	Bihar	21.9 (14.4 to 28.8)
Odisha	4.3 (3.8 to 4.8)	Jharkhand	6.4 (5.6 to 7.3)	Mizoram	21.7 (15.6 to 28.1)
Mizoram	4.2 (3.7 to 4.7)	Jammu and Kashmir	6.3 (5.5 to 7.1)	Telangana	21.7 (16.1 to 27.4)
Sikkim	4.2 (3.7 to 4.7)	West Bengal	6.2 (5.5 to 6.9)	Delhi	20.3 (14.7 to 26.4)
Meghalaya	4.1 (3.6 to 4.6)	Arunachal Pradesh	5.8 (5.2 to 6.5)	Jharkhand	19.7 (13.5 to 26.1)
Rajasthan	4.1 (3.6 to 4.6)	Himachal Pradesh	5.8 (5.1 to 6.5)	Karnataka	19.3 (14.2 to 24.8)
Arunachal Pradesh	4.0 (3.5 to 4.5)	Rajasthan	5.7 (5.1 to 6.3)	Assam	17.4 (10.0 to 25.7)
Jammu and Kashmir	4.0 (3.5 to 4.5)	Bihar	5.6 (4.9 to 6.2)	Maharashtra	16.4 (11.3 to 21.0)
West Bengal	3.9 (3.5 to 4.4)	Mizoram	5.5 (5.0 to 6.2)	Manipur	15.6 (8.0 to 22.8)
Himachal Pradesh	3.8 (3.3 to 4.2)	Nagaland	5.4 (4.8 to 6.0)	Union territories other than Delhi	9.8 (5.1 to 14.8)
Nagaland	3.5 (3.1 to 4.0)	Meghalaya	5.3 (4.8 to 5.8)	Kerala	5.4 (1.4 to 9.6)

*The states are listed in decreasing order of each estimate.

5. Age-sex-specific prevalence of diabetes in India, 1990 and 2016

Age groups (years)	Prevalence (95% uncertainty interval)			
	Men		Women	
	1990	2016	1990	2016
**Under 5**	0.0 (0.0 to 0.0)	0.0 (0.0 to 0.0)	0.0 (0.0 to 0.0)	0.0 (0.0 to 0.0)
**5 to 9**	0.05 (0.03 to 0.07)	0.06 (0.03 to 0.08)	0.06 (0.04 to 0.09)	0.06 (0.04 to 0.09)
**10 to 14**	0.3 (0.2 to 0.4)	0.3 (0.2 to 0.5)	0.4 (0.3 to 0.4)	0.4 (0.3 to 0.5)
**15 to 19**	0.8 (0.6 to 1.0)	1.0 (0.7 to 1.2)	0.8 (0.7 to 1.0)	0.9 (0.7 to 1.2)
**20 to 24**	1.5 (1.2 to 1.8)	1.8 (1.4 to 2.2)	1.4 (1.1 to 1.7)	1.5 (1.2 to 1.9)
**25 to 29**	2.3 (1.9 to 2.8)	2.7 (2.2 to 3.3)	2.0 (1.6 to 2.4)	2.2 (1.8 to 2.7)
**30 to 34**	3.4 (2.8 to 4.0)	4.0 (3.3 to 4.8)	2.8 (2.3 to 3.3)	3.0 (2.5 to 3.6)
**35 to 39**	5.1 (4.3 to 5.9)	5.9 (5.0 to 6.9)	4.0 (3.4 to 4.7)	4.5 (3.8 to 5.2)
**40 to 44**	7.0 (5.9 to 8.2)	8.4 (7.1 to 9.8)	5.5 (4.6 to 6.5)	6.4 (5.4 to 7.7)
**45 to 49**	8.8 (7.5 to 10.2)	11.1 (9.6 to 12.7)	7.2 (6.2 to 8.4)	8.9 (7.6 to 10.3)
**50 to 54**	10.1 (8.7 to 11.5)	13.6 (11.8 to 15.4)	8.9 (7.6 to 10.2)	11.6 (9.9 to 13.2)
**55 to 59**	11.6 (10.2 to 13.1)	16.1 (14.3 to 18.1)	10.4 (9.1 to 11.7)	13.5 (11.9 to 15.2)
**60 to 64**	12.5 (11.1 to 14.0)	17.7 (15.8 to 19.8)	11.1 (9.9 to 12.5)	14.7 (13.1 to 16.6)
**65 to 69**	13.3 (11.9 to 14.9)	18.8 (16.7 to 21.1)	11.9 (10.5 to 13.4)	15.4 (13.6 to 17.4)
**70 to 74**	13.3 (11.8 to 14.8)	19.3 (17.2 to 21.7)	11.8 (10.5 to 13.4)	15.8 (13.9 to 17.8)
**75 to 79**	13.6 (12.2 to 15.2)	19.9 (17.8 to 22.4)	12.4 (11.1 to 13.9)	16.4 (14.6 to 18.4)
**80+**	12.5 (11.1 to 14.1)	19.0 (16.9 to 21.5)	11.9 (10.5 to 13.4)	16.1 (14.1 to 18.2)

6. Percent of total deaths and DALYs due to diabetes in the states of India grouped by epidemiological transition level, 2016

State group	Percent of total deaths (95% uncertainty interval)			Percent of total DALYs (95% uncertainty interval)		
	Both	Men	Women	Both	Men	Women
**Low ETL**	2.4 (2.1 to 2.5)	2.4 (2.2 to 2.6)	2.3 (2.0 to 2.6)	1.7 (1.5 to 1.9)	1.8 (1.7 to 2.0)	1.5 (1.4 to 1.7)
**Lower-middle ETL**	3.1 (2.9 to 3.3)	2.6 (2.4 to 2.8)	3.7 (3.3 to 4.0)	2.2 (2.0 to 2.4)	2.1 (1.9 to 2.3)	2.3 (2.1 to 2.5)
**Higher-middle ETL**	3.3 (3.1 to 3.4)	2.9 (2.7 to 3.0)	3.8 (3.3 to 4.1)	2.4 (2.2 to 2.7)	2.4 (2.2 to 2.7)	2.5 (2.3 to 2.7)
**High ETL**	5.9 (5.4 to 6.2)	5.0 (4.6 to 5.4)	6.9 (6.1 to 7.6)	4.3 (4.0 to 4.7)	4.0 (3.7 to 4.4)	4.7 (4.3 to 5.1)
**India**	3.1 (2.9 to 3.3)	2.9 (2.7 to 3.0)	3.4 (3.0 to 3.7)	2.2 (2.1 to 2.4)	2.3 (2.1 to 2.5)	2.2 (2.0 to 2.4)

DALY is disability-adjusted life-year. ETL is epidemiological transition level.

7. Change in DALY rate of diabetes in the states of India, 1990 to 2016

States of India*	Crude DALY rate per 100,000 (95% uncertainty interval)			Age-standardised percent change 1990 to 2016 (95% uncertainty interval)
	1990	2016	Percent change 1990 to 2016	
Nagaland	234 (191 to 289)	440 (360 to 536)	87.8 (68.3 to 111.7)	75.1 (54.8 to 101.2)
Chhattisgarh	351 (291 to 424)	776 (649 to 917)	120.8 (98.7 to 145.1)	65.0 (47.5 to 83.8)
Haryana	394 (329 to 467)	817 (695 to 960)	107.2 (86.3 to 130.4)	61.8 (43.4 to 81.1)
Uttar Pradesh	362 (307 to 432)	651 (552 to 766)	79.6 (62.7 to 99.8)	61.2 (44.6 to 79.4)
Madhya Pradesh	388 (314 to 478)	741 (615 to 892)	91.0 (75.8 to 108.3)	60.8 (47.0 to 75.9)
Arunachal Pradesh	310 (255 to 371)	527 (431 to 627)	69.9 (49.1 to 93.2)	55.5 (34.1 to 79.5)
Jammu and Kashmir	295 (244 to 360)	586 (480 to 701)	98.6 (80.3 to 120.4)	55.0 (39.1 to 72.4)
Tripura	390 (311 to 484)	734 (589 to 894)	87.8 (69.3 to 109.5)	51.8 (35.1 to 70.4)
Odisha	364 (301 to 434)	730 (605 to 863)	100.3 (80.4 to 122.6)	50.5 (34.8 to 67.6)
Tamil Nadu	778 (660 to 923)	1,628 (1,385 to 1,915)	109.3 (89.5 to 134.0)	48.9 (34.4 to 67.1)
Meghalaya	284 (230 to 344)	468 (391 to 557)	64.7 (47.2 to 85.7)	46.1 (27.8 to 66.0)
Punjab	652 (547 to 771)	1,314 (1,119 to 1,529)	101.5 (78.5 to 126.5)	42.9 (25.8 to 61.0)
Uttarakhand	384 (322 to 453)	795 (666 to 933)	107.0 (84.9 to 130.0)	42.9 (26.3 to 59.5)
Gujarat	387 (320 to 462)	729 (619 to 866)	88.5 (70.7 to 107.2)	42.2 (27.6 to 57.2)
Goa	475 (396 to 567)	1,090 (910 to 1,294)	129.4 (103.4 to 162.9)	41.3 (24.2 to 63.1)
West Bengal	300 (250 to 364)	600 (495 to 731)	99.9 (83.7 to 118.1)	41.2 (28.2 to 54.5)
Bihar	379 (319 to 447)	611 (519 to 710)	61.0 (42.4 to 83.5)	40.0 (22.3 to 60.4)
Karnataka	632 (532 to 753)	1,202 (1,017 to 1,409)	90.3 (71.3 to 112.8)	39.6 (24.2 to 56.3)
Sikkim	323 (265 to 386)	549 (441 to 663)	70.0 (50.4 to 90.3)	37.0 (19.2 to 55.9)
Himachal Pradesh	272 (220 to 335)	512 (412 to 632)	88.3 (69.3 to 107.5)	33.9 (19.0 to 48.1)
Rajasthan	269 (218 to 332)	443 (364 to 543)	64.7 (52.8 to 78.3)	33.6 (23.2 to 45.4)
Manipur	561 (461 to 669)	947 (801 to 1,109)	68.9 (45.4 to 94.0)	29.6 (9.8 to 49.9)
Assam	457 (389 to 537)	787 (670 to 917)	72.3 (53.4 to 92.4)	29.5 (14.7 to 46.0)
Andhra Pradesh	473 (393 to 569)	841 (698 to 1,006)	77.6 (58.6 to 102.2)	28.2 (12.9 to 46.7)
Telangana	424 (347 to 513)	733 (601 to 886)	72.7 (53.1 to 96.9)	23.9 (7.9 to 41.4)
Mizoram	309 (251 to 378)	513 (421 to 623)	66.1 (47.2 to 87.6)	20.1 (4.7 to 37.1)
Maharashtra	477 (400 to 571)	788 (659 to 939)	65.1 (49.6 to 81.4)	17.9 (5.5 to 29.9)
Jharkhand	435 (361 to 522)	632 (524 to 761)	45.2 (30.3 to 62.5)	17.3 (4.3 to 31.5)
Kerala	600 (466 to 750)	1,094 (883 to 1,347)	82.4 (70.3 to 95.9)	16.8 (8.5 to 25.5)
Union Territories other than Delhi	537 (435 to 654)	784 (635 to 957)	45.9 (30.9 to 63.8)	15.2 (2.2 to 30.5)
Delhi	575 (460 to 700)	869 (697 to 1,072)	51.0 (36.9 to 65.1)	12.6 (-0.4 to 25.0)

*The states are listed in descending order of age-standardised percent change. DALY is disability-adjusted life-year.

8. Change in DALY number and percent change in rates for the leading 30 causes in India, 1990 to 2016

Leading causes 1990	Leading causes 2016	Mean % change number of DALYs 1990–2016	Mean % change all-age DALY rate 1990–2016	Mean % change age-standardised DALY rate 1990–2016
1 Diarrhoeal diseases	1 Ischaemic heart disease	104·1% (90·1 to 118·8)	33·9% (24·7 to 43·6)	2·2% (–4·8 to 9·7)
2 Lower respiratory infections	2 COPD	36·3% (21·1 to 56·8)	–10·5% (–20·5 to 2·9)	–35·9% (–42·7 to –26·1)
3 Neonatal preterm birth	3 Diarrhoeal diseases	–67·7% (–73·8 to –58·8)	–78·8% (–82·8 to –73·0)	–71·3% (–75·9 to –65·1)
4 Tuberculosis	4 Lower respiratory infections	–61·5% (–67·3 to –53·8)	–74·7% (–78·6 to –69·7)	–59·1% (–64·9 to –51·2)
5 Measles	5 Cerebrovascular disease	52·9% (40·4 to 66·7)	0·4% (–7·9 to 9·4)	–25·7% (–32·0 to –18·8)
6 Ischaemic heart disease	6 Iron-deficiency anaemia	41·8% (39·9 to 43·8)	–6·9% (–8·2 to –5·6)	0·1% (–0·8 to 1·0)
7 Other neonatal	7 Neonatal preterm birth	–46·3% (–55·4 to –37·1)	–64·8% (–70·7 to –58·7)	–40·4% (–50·1 to –30·5)
8 COPD	8 Tuberculosis	–44·5% (–50·1 to –39·1)	–63·5% (–67·3 to –60·0)	–69·2% (–73·0 to –66·2)
9 Neonatal encephalopathy	9 Sense organ diseases	85·3% (83·0 to 87·8)	21·7% (20·1 to 23·3)	–4·4% (–5·3 to –3·5)
10 Iron-deficiency anaemia	10 Road injuries	65·1% (53·4 to 76·6)	8·3% (0·7 to 15·9)	3·9% (–2·9 to 10·6)
11 Congenital defects	11 Self-harm	29·8% (15·2 to 52·4)	–14·8% (–24·4 to 0·1)	–19·5% (–28·2 to –5·7)
12 Cerebrovascular disease	12 Low back and neck pain	66·1% (62·0 to 69·8)	9·0% (6·3 to 11·4)	–11·6% (–12·8 to –10·3)
13 Tetanus	13 Diabetes	174·2% (161·4 to 187·1)	80·0% (71·6 to 88·5)	39·6% (32·1 to 46·7)
14 Self-harm	14 Other neonatal	–49·7% (–60·5 to –36·3)	–67·0% (–74·0 to –58·2)	–41·5% (–54·0 to –25·8)
15 Intestinal infections	15 Migraine	69·1% (67·0 to 71·2)	11·0% (9·6 to 12·3)	–0·7% (–1·6 to 0·1)
16 Road injuries	16 Skin diseases	55·0% (50·3 to 59·8)	1·7% (–1·4 to 4·9)	5·3% (2·1 to 8·6)
17 Sense organ diseases	17 Falls	41·3% (17·4 to 59·5)	–7·2% (–23·0 to 4·7)	–12·6% (–25·1 to –4·2)
18 Meningitis	18 Congenital defects	–20·9% (–47·6 to 11·5)	–48·1% (–65·6 to –26·8)	–20·3% (–46·8 to 10·1)
19 Asthma	19 Other musculoskeletal	79·7% (75·4 to 84·4)	18·0% (15·1 to 21·0)	–1·3% (–2·9 to 0·3)
20 Low back and neck pain	20 Chronic kidney disease	71·0% (55·8 to 87·9)	12·2% (2·3 to 23·3)	–8·3% (–16·4 to 0·4)
21 Falls	21 Depressive disorders	65·1% (60·6 to 69·6)	8·4% (5·4 to 11·3)	–7·9% (–9·8 to –5·9)
22 Protein-energy malnutrition	22 Neonatal encephalopathy	–56·1% (–65·0 to –45·3)	–71·2% (–77·1 to –64·1)	–49·2% (–59·5 to –37·1)
23 Skin diseases	23 Asthma	–15·1% (–31·8 to 1·3)	–44·3% (–55·2 to –33·5)	–53·6% (–64·1 to –44·0)
24 Migraine	24 Intestinal infections	–37·1% (–48·5 to –26·5)	–58·7% (–66·2 to –51·8)	–49·5% (–58·0 to –42·1)
25 Malaria	25 HIV/AIDS	1004·6% (921·3 to 1090·1)	625·0% (570·3 to 681·2)	568·5% (517·2 to 620·8)
26 Drowning	26 Anxiety disorders	61·9% (57·2 to 66·7)	6·2% (3·2 to 9·4)	–3·6% (–6·1 to –0·9)
27 Neonatal haemolytic	27 Meningitis	–46·7% (–59·8 to –12·5	–65·0% (–73·6 to –42·6)	–54·4% (–65·2 to –26·6)
28 Neonatal sepsis	28 Rheumatic heart disease	2·5% (–14·6 to 18·8)	–32·7% (–43·9 to –22·0)	–39·8% (–50·3 to –28·7)
29 Depressive disorders	29 Protein-energy malnutrition	–42·3% (–55·2 to –26·7)	–62·1% (–70·6 to –51·9)	–40·3% (–53·4 to –24·3)
30 Chronic kidney disease	30 Drowning	–36·0% (–47·0 to –15·2)	–58·0% (–65·2 to –44·4)	–48·2% (–56·3 to –33·9)
32 Other musculoskeletal	33 Malaria			
35 Diabetes	38 Neonatal sepsis			
37 Rheumatic heart disease	59 Measles			
41 Anxiety disorders	66 Neonatal haemolytic			
102 HIV/AIDS	109 Tetanus			

Communicable, maternal, neonatal, and nutritional diseases
Non-communicable diseases
Injuries

Causes are connected by lines between time periods. Three measures of change are shown: percent change in the number of DALYs, percent change in all-age DALY rate and percent change in age-standardised DALY rate.
DALY is disability-adjusted life-year. COPD is chronic obstructive pulmonary disease.

Source: India State-Level Disease Burden Initiative Collaborators. Nations within a nation: variations in epidemiological transition across the states of India, 1990–2016 in the Global Burden of Disease Study. *Lancet* 2017; **390**: 2437–60.

9. Percent contribution of major risk factors to diabetes DALYs in the states of India grouped by epidemiological transition level, 2016

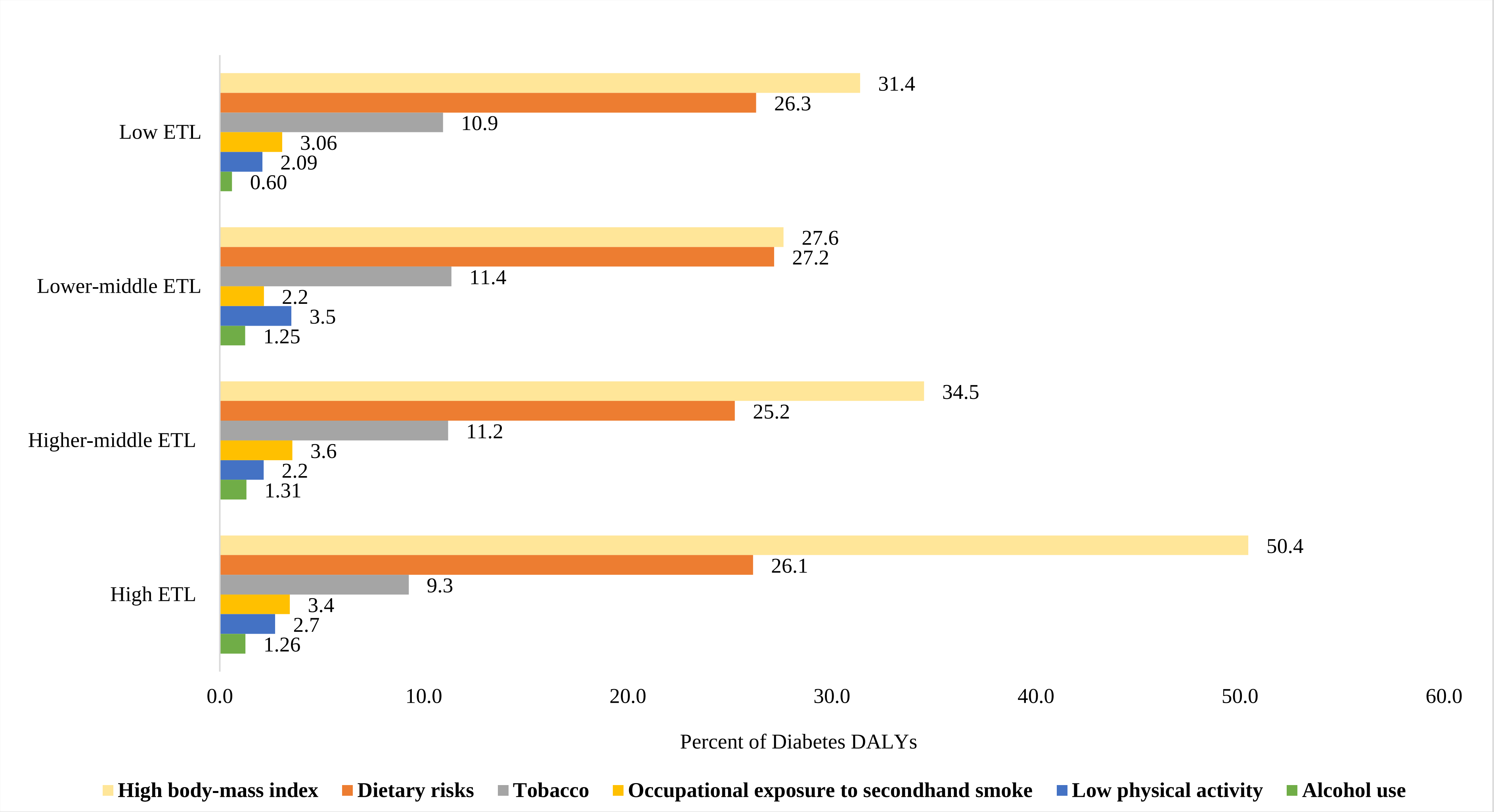

The cumulative impact of risk factors is not the simple addition of their individual contributions as the risk factors overlap, and also because the population attributable fractions from components can add up to more than their sum even if they are independent. DALY is disability-adjusted life-year. ETL is epidemiological transition level.

10. Prevalence of overweight in adults 20 years of age or more in the states of India, 1990 and 2016

States of India	Sex	Crude prevalence per 100 (95% uncertainty interval)			Age-standardised percent change 1990 to 2016 (95% uncertainty interval)
		1990	2016	Percent change 1990 to 2016	
**India**	Both sexes	9.0 (8.7 to 9.3)	20.4 (19.9 to 20.8)	126.9 (117.8 to 136.8)	119.0 (110.8 to 128.5)
	Men	8.5 (8.1 to 9.0)	19.6 (18.9 to 20.3)	130.2 (116.2 to 145.5)	122.3 (109.6 to 136.7)
	Women	9.5 (9.1 to 9.9)	21.2 (20.5 to 21.8)	123.6 (112.0 to 136.1)	115.6 (104.5 to 126.5)
**Low ETL**	Both sexes	8.7 (8.2 to 9.2)	16.9 (16.2 to 17.8)	94.9 (80.8 to 110.7)	89.2 (76.8 to 103.7)
	Men	9.1 (8.3 to 10.0)	17.2 (16.0 to 18.5)	87.9 (67.6 to 113.1)	82.9 (63.9 to 105.4)
	Women	8.2 (7.6 to 8.9)	16.7 (15.7 to 17.7)	103.3 (83.6 to 124.1)	96.2 (78.0 to 115.6)
Bihar	Both sexes	6.8 (5.8 to 8.0)	10.8 (9.2 to 12.5)	58.2 (25.5 to 97.8)	54.8 (25.3 to 89.3)
	Men	6.4 (5.0 to 8.2)	10.6 (8.5 to 13.2)	66.0 (21.7 to 127.6)	63.7 (21.7 to 120.1)
	Women	7.2 (5.8 to 8.9)	10.9 (8.8 to 13.4)	50.9 (10.7 to 104.3)	47.0 (10.3 to 96.5)
Jharkhand	Both sexes	6.7 (5.7 to 7.8)	13.4 (11.6 to 15.4)	100.4 (61.8 to 149.7)	94.0 (60.0 to 137.5)
	Men	6.5 (5.1 to 8.1)	12.5 (10.3 to 15.4)	92.4 (43.2 to 166.0)	88.4 (41.6 to 156.7)
	Women	6.9 (5.5 to 8.4)	14.3 (11.9 to 17.2)	108.9 (55.0 to 177.5)	99.7 (49.9 to 160.8)
Uttar Pradesh	Both sexes	10.9 (9.8 to 12.1)	19.7 (17.9 to 21.8)	80.7 (55.2 to 108.6)	76.7 (52.2 to 102.0)
	Men	11.5 (9.6 to 13.4)	20.1 (17.3 to 23.3)	74.9 (41.5 to 120.6)	71.5 (37.7 to 114.3)
	Women	10.2 (8.8 to 11.7)	19.2 (16.8 to 21.6)	88.1 (54.2 to 125.9)	82.8 (50.6 to 118.5)
Rajasthan	Both sexes	9.2 (8.0 to 10.7)	20.0 (17.7 to 22.6)	117.3 (78.7 to 162.6)	114.4 (81.3 to 153.6)
	Men	10.4 (8.3 to 13.0)	21.4 (17.8 to 25.6)	104.8 (55.6 to 174.0)	105.4 (59.9 to 166.2)
	Women	7.9 (6.7 to 9.3)	18.6 (15.8 to 21.3)	135.6 (88.4 to 191.8)	125.7 (82.6 to 174.9)
Meghalaya	Both sexes	6.0 (5.0 to 7.1)	13.5 (12.1 to 14.9)	125.4 (85.4 to 176.6)	118.9 (84.5 to 161.4)
	Men	6.3 (4.8 to 8.0)	12.9 (10.9 to 15.1)	105.8 (54.4 to 177.1)	107.5 (60.2 to 174.1)
	Women	5.7 (4.5 to 7.1)	14.1 (12.3 to 16.1)	148.1 (89.1 to 221.1)	129.2 (80.2 to 191.7)
Assam	Both sexes	10.9 (9.2 to 12.7)	23.7 (21.0 to 26.8)	118.6 (78.7 to 170.3)	107.8 (72.5 to 148.8)
	Men	10.9 (8.5 to 13.7)	22.2 (18.3 to 26.5)	103.9 (51.8 to 172.2)	97.4 (53.2 to 155.2)
	Women	10.9 (8.7 to 13.5)	25.4 (21.6 to 29.7)	134.2 (78.7 to 202.2)	116.5 (69.9 to 171.2)
Chhattisgarh	Both sexes	7.1 (6.1 to 8.1)	16.7 (14.9 to 18.7)	136.2 (96.6 to 185.4)	133.2 (96.0 to 179.8)
	Men	7.2 (5.8 to 8.8)	17.4 (14.6 to 20.7)	143.4 (87.4 to 220.2)	142.1 (88.6 to 209.7)
	Women	7.0 (5.6 to 8.6)	15.9 (13.5 to 18.6)	128.7 (78.6 to 191.8)	125.0 (78.6 to 188.4)
Madhya Pradesh	Both sexes	6.8 (5.9 to 7.7)	13.6 (12.2 to 15.1)	101.3 (72.1 to 138.6)	96.0 (68.8 to 130.3)
	Men	7.6 (6.2 to 9.3)	14.0 (11.9 to 16.2)	82.7 (43.2 to 133.3)	78.1 (42.6 to 124.4)
	Women	5.8 (4.8 to 7.0)	13.2 (11.2 to 15.3)	126.9 (80.1 to 183.1)	121.4 (76.3 to 175.4)
Odisha	Both sexes	6.8 (5.8 to 8.0)	16.9 (14.9 to 19.0)	146.5 (101.2 to 201.5)	135.1 (93.3 to 187.5)
	Men	7.1 (5.6 to 9.0)	16.8 (14.1 to 19.7)	136.3 (78.9 to 218.8)	125.3 (72.9 to 197.5)
	Women	6.6 (5.3 to 8.1)	16.9 (14.4 to 19.7)	158.2 (99.7 to 239.4)	146.3 (93.4 to 219.8)
**Lower-middle ETL**	Both sexes	7.0 (6.4 to 7.7)	16.9 (15.8 to 18.0)	140.5 (115.6 to 170.0)	133.2 (110.9 to 158.8)
	Men	5.7 (5.0 to 6.5)	14.2 (13.0 to 15.4)	148.8 (113.8 to 190.4)	140.9 (110.5 to175.3)
	Women	8.4 (7.3 to 9.6)	19.7 (18.0 to 21.4)	134.5 (97.0 to 174.7)	127.8 (94.3 to 164.7)
Arunachal Pradesh	Both sexes	8.1 (6.7 to 9.6)	18.9 (17.4 to 20.5)	134.0 (92.4 to 191.1)	126.4 (89.9 to 173.5)
	Men	8.2 (6.4 to 10.5)	18.4 (16.4 to 20.6)	123.7 (71.1 to 199.9)	118.9 (71.2 to 185.5)
	Women	7.9 (6.1 to 10.1)	19.4 (17.3 to 21.7)	147.0 (89.5 to 227.3)	135.0 (82.0 to 204.7)
Mizoram	Both sexes	8.4 (7.2 to 9.7)	17.6 (16.2 to 19.3)	109.7 (77.5 to 148.1)	100.8 (72.7 to 133.1)
	Men	9.2 (7.5 to 11.3)	17.6 (15.6 to 19.8)	90.9 (50.5 to 144.1)	78.1 (43.5 to 121.3)
	Women	7.5 (5.9 to 9.1)	17.7 (15.7 to 19.9)	137.2 (89.2 to 202.6)	134.0 (88.6 to 190.7)
Nagaland	Both sexes	6.5 (5.4 to 7.7)	13.0 (11.8 to 14.3)	99.6 (64.0 to 145.3)	95.2 (62.4 to 137.6)
	Men	6.2 (4.8 to 8.0)	13.0 (11.4 to 14.9)	108.4 (59.4 to 181.6)	105.5 (62.0 to 172.3)
	Women	6.8 (5.3 to 8.8)	13.0 (11.4 to 14.9)	90.0 (40.4 to 157.6)	84.4 (40.9 to 143.6)
Uttarakhand	Both sexes	11.3 (9.9 to 12.8)	32.6 (29.8 to 35.5)	187.7 (147.3 to 239.1)	179.1 (141.4 to 224.3)
	Men	11.0 (9.0 to 13.0)	30.4 (27.1 to 34.1)	176.7 (124.3 to 244.8)	170.3 (121.7 to 233.6)
	Women	11.7 (9.7 to 14.0)	34.8 (30.8 to 39.2)	197.9 (142.8 to 274.1)	186.5 (133.7 to 258.7)
Gujarat	Both sexes	6.3 (5.4 to 7.1)	14.5 (13.2 to 15.9)	131.8 (97.2 to 175.9)	125.9 (95.0 to 163.9)
	Men	4.7 (3.8 to 5.6)	11.6 (10.1 to 13.1)	148.9 (98.3 to 220.9)	141.9 (95.9 to 201.3)
	Women	8.0 (6.6 to 9.6)	17.7 (15.5 to 20.0)	121.6 (75.3 to 176.5)	116.3 (73.8 to 165.9)
Tripura	Both sexes	6.4 (5.3 to 7.6)	14.4 (13.1 to 15.9)	125.3 (83.8 to 179.1)	119.0 (80.9 to 165.1)
	Men	6.6 (5.1 to 8.4)	13.5 (11.6 to 15.7)	103.8 (55.5 to 174.3)	100.5 (55.2 to 164.7)
	Women	6.1 (4.8 to 7.8)	15.4 (13.6 to 17.4)	150.3 (91.7 to 230.6)	140.3 (88.7 to 212.7)
Sikkim	Both sexes	10.4 (8.6 to 12.2)	32.3 (30.1 to 34.8)	210.5 (160.5 to 276.2)	198.1 (155.9 to 254.4)
	Men	10.0 (7.8 to 12.5)	28.7 (25.6 to 31.9)	187.8 (122.6 to 276.6)	181.5 (122.6 to 256.1)
	Women	11.0 (8.7 to 13.5)	36.8 (33.9 to 40.1)	235.8 (168.1 to 330.1)	216.5 (159.7 to 297.9)
Manipur	Both sexes	8.9 (7.6 to 20.4)	17.4 (16.1 to 18.8)	94.9 (64.7 to 131.5)	87.2 (60.3 to 120.4)
	Men	7.4 (5.8 to 9.5)	15.2 (13.5 to 17.0)	104.6 (55.4 to 166.8)	97.8 (55.3 to 153.0)
	Women	10.5 (8.6 to 12.8)	19.7 (17.8 to 21.7)	87.2 (51.8 to 135.6)	79.7 (48.3 to 123.4)

States of India	Sex	Crude prevalence per 100 (95% uncertainty interval)			Age-standardised percent change 1990 to 2016 (95% uncertainty interval)
		1990	2016	Percent change 1990 to 2016	
**Higher-middle ETL**	Both sexes	8.2 (7.7 to 8.6)	20.9 (20.1 to 21.6)	156.0 (139.4 to 174.5)	149.2 (134.0 to 166.4)
	Men	7.4 (6.8 to 8.0)	20.0 (19.0 to 21.0)	171.3 (145.3 to 198.1)	163.6 (137.4 to 194.3)
	Women	9.0 (8.3 to 9.7)	21.8 (20.8 to 22.8)	142.2 (122.6 to 166.0)	136.2 (117.6 to 158.0)
Haryana	Both sexes	12.4 (10.8 to 13.9)	26.4 (24.8 to 28.2)	113.5 (87.4 to 147.1)	104.7 (80.8 to 134.8)
	Men	10.7 (8.8 to 12.8)	24.9 (22.6 to 27.4)	132.4 (88.4 to 188.5)	124.9 (86.4 to 174.4)
	Women	14.3 (12.0 to 16.7)	28.1 (25.8 to 30.5)	97.0 (64.9 to 138.3)	87.7 (58.7 to 124.3)
Delhi	Both sexes	17.2 (14.8 to 19.9)	31.8 (28.6 to 35.3)	85.5 (55.4 to 123.1)	75.7 (51.4 to 105.6)
	Men	14.0 (11.0 to 17.3)	31.3 (26.3 to 36.0)	122.9 (71.1 to 198.2)	112.4 (70.0 to 172.3)
	Women	21.1 (17.3 to 25.2)	32.4 (27.9 to 37.1)	53.6 (22.3 to 96.3)	45.9 (19.8 to 81.0)
Telangana	Both sexes	17.8 (15.3 to 20.6)	24.3 (22.7 to 25.8)	36.1 (16.1 to 59.4)	32.0 (14.2 to 53.9)
	Men	17.7 (14.5 to 21.7)	24.6 (22.4 to 26.9)	38.9 (12.0 to 72.0)	35.7 (11.1 to 66.6)
	Women	17.9 (14.7 to 22.0)	23.9 (21.7 to 26.1)	33.4 (6.7 to 66.2)	28.4 (3.8 to 57.4)
Andhra Pradesh	Both sexes	6.4 (5.6 to 7.4)	26.4 (24.4 to 28.3)	310.9 (251.0 to 382.1)	313.3 (257.3 to 380.3)
	Men	5.6 (4.6 to 6.7)	25.6 (22.8 to 28.6)	360.9 (270.8 to 477.3)	363.8 (279.4 to 468.4)
	Women	7.3 (6.0 to 8.9)	27.2 (24.4 to 29.8)	272.0 (199.6 to 366.7)	274.0 (204.3 to 362.0)
Jammu and Kashmir	Both sexes	12.5 (10.8 to 14.4)	27.5 (24.8 to 30.3)	120.7 (86.7 to 160.7)	116.2 (86.5 to 151.3)
	Men	13.1 (10.8 to 15.9)	29.7 (25.4 to 34.2)	126.0 (77.0 to 182.6)	122.1 (79.4 to 175.5)
	Women	11.6 (9.5 to 14.1)	24.9 (21.3 to 28.5)	114.1 (67.1 to 171.5)	109.0 (67.5 to 160.7)
Karnataka	Both sexes	5.0 (4.4 to 5.8)	12.9 (11.6 to 14.3)	155.3 (114.2 to 202.9)	151.2 (112.2 to 193.3)
	Men	3.4 (2.8 to 4.1)	9.6 (8.3 to 11.0)	178.2 (123.2 to 257.3)	168.5 (118.1 to 236.9)
	Women	6.7 (5.5 to 8.2)	16.2 (14.0 to 18.7)	141.7 (88.8 to 207.4)	139.9 (88.6 to 201.3)
West Bengal	Both sexes	4.6 (4.0 to 5.3)	15.4 (13.9 to 16.8)	236.2 (183.3 to 295.5)	217.6 (168.4 to 272.0)
	Men	3.4 (2.8 to 4.1)	13.9 (12.2 to 15.9)	307.7 220.5 to 416.7)	269.3 (196.6 to 360.1)
	Women	5.9 (4.8 to 7.2)	16.9 (14.9 to 19.0)	186.8 (128.2 to 259.3)	177.3 (123.1 to 243.1)
Maharashtra	Both sexes	8.2 (7.2 to 9.4)	22.3 (20.4 to 24.1)	170.4 (130.3 to 220.9)	168.5 (132.3 to 216.0)
	Men	8.4 (6.9 to 10.1)	22.3 (19.6 to 25.0)	166.4 (115.3 to 237.2)	165.1 (118.8 to 230.7)
	Women	8.1 (6.6 to 9.8)	22.2 (19.8 to 24.7)	174.9 (120.8 to 248.9)	171.9 (119.6 to 239.0)
Union territories other than Delhi	Both sexes	13.5 (11.6 to 15.5)	35.8 (32.4 to 39.5)	165.5 (122.2 to 218.2)	156.5 (118.8 to 202.9)
	Men	12.7 (10.1 to 15.7)	36.3 (31.0 to 41.8)	185.3 (119.4 to 274.2)	177.2 (117.9 to 252.7)
	Women	14.4 (11.7 to 17.2)	35.2 (30.3 to 40.6)	145.1 (92.4 to 206.6)	136.4 (89.4 to 191.7)
**High ETL**	Both sexes	12.7 (11.8 to 13.6)	32.2 (31.1 to 33.4)	153.8 (134.0 to 175.8)	140.4 (123.1 to 160.3)
	Men	10.8 (9.7 to 11.9)	29.9 (28.4 to 31.6)	177.9 (148.7 to 211.9)	163.7 (137.4 to 194.3)
	Women	14.7 (13.3 to 16.1)	34.5 (32.8 to 36.3)	135.2 (97.0 to 174.7)	122.8 (101.1 to 148.4)
Himachal Pradesh	Both sexes	9.0 (7.6 to 10.7)	22.9 (20.7 to 25.0)	155.0 (109.2 to 206.3)	143.7 (101.1 to 194.2)
	Men	8.5 (6.6 to 10.8)	22.9 (19.8 to 26.2)	168.8 (104.7 to 255.8)	159.9 (100.3 to 240.5)
	Women	9.4 (7.4 to 12.1)	22.8 (20.0 to 25.6)	142.4 (82.9 to 219.4)	129.1 (73.8 to 199.3)
Punjab	Both sexes	18.7 (16.5 to 21.2)	37.6 (36.0 to 39.4)	100.8 (76.8 to 131.5)	92.7 (70.4 to 118.8)
	Men	15.7 (13.2 to 18.6)	35.2 (32.8 to 37.7)	124.1 (89.0 to 172.8)	115.2 (82.4 to 156.8)
	Women	22.1 (18.8 to 25.9)	40.3 (37.9 to 42.7)	81.8 (54.0 to 117.9)	74.6 (49.0 to 108.0)
Tamil Nadu	Both sexes	11.7 (10.2 to 13.1)	31.0 (29.0 to 33.1)	166.1 (76.8 to 131.5)	150.6 (119.9 to 186.3)
	Men	9.8 (8.2 to 11.6)	28.3 (25.5 to 31.2)	189.3 (136.7 to 256.1)	171.9 (123.7 to 228.2)
	Women	13.6 (11.4 to 16.2)	33.7 (30.7 to 36.8)	148.3 (104.9 to 203.8)	134.4 (95.7 to 186.0)
Goa	Both sexes	15.9 (14.0 to 18.2)	36.7 (34.4 to 39.0)	130.0 (99.7 to 167.2)	113.7 (87.1 to 146.6)
	Men	16.1 (13.1 to 19.3)	36.1 (32.9 to 39.3)	124.6 (81.1 to 182.2)	109.7 (71.9 to 159.0)
	Women	15.8 (13.4 to 18.7)	37.3 (34.1 to 40.5)	135.6 (95.6 to 183.6)	118.6 (82.4 to 161.2)
Kerala	Both sexes	11.3 (10.0 to 12.7)	32.1 (30.2 to 33.9)	184.3 (149.7 to 224.7)	169.2 (136.8 to 205.4)
	Men	9.4 (7.8 to 11.3)	30.1 (27.4 to 32.8)	218.8 (157.4 to 294.5)	205.7 (150.3 to 272.1)
	Women	13.0 (11.2 to 15.2)	33.9 (31.4 to 36.5)	159.8 (120.7 to 207.9)	144.7 (108.2 to 191.0)

ETL is epidemiological transition level.
